# A Novel Educational Strategy Targeting Health Care Workers in Underserved Communities in Central America to Integrate HIV into Primary Medical Care

**DOI:** 10.1371/journal.pone.0046426

**Published:** 2012-10-24

**Authors:** Tamara Flys, Rosalba González, Omar Sued, Juana Suarez Conejero, Edgar Kestler, Nestor Sosa, Jane McKenzie-White, Irma Irene Monzón, Carmen-Rosa Torres, Kathleen Page

**Affiliations:** 1 Johns Hopkins School of Medicine, Baltimore, Maryland, United States of America; 2 Gorgas Memorial Institute, Panama City, Panama; 3 Pan American Health Organization, Washington, D.C., United States of America; 4 Pan American Health Organization, Consultant, Washington, D.C., United States of America; 5 Centro de Investigación Epidemiológica en Salud Sexual y Reproductiva (CIESAR), Guatemala; 6 U.S. Department of Health and Human Services (HHS), Washington, D.C., United States of America; University of Ottawa, Canada

## Abstract

**Background:**

Current educational strategies to integrate HIV care into primary medical care in Central America have traditionally targeted managers or higher-level officials, rather than local health care workers (HCWs). We developed a complementary online and on-site interactive training program to reach local HCWs at the primary care level in underserved communities.

**Methods:**

The training program targeted physicians, nurses, and community HCWs with limited access to traditional onsite training in Panama, Nicaragua, Dominican Republic, and Guatemala. The curriculum focused on principles of HIV care and health systems using a tutor-supported blended educational approach of an 8-week online component, a weeklong on-site problem-solving workshop, and individualized project-based interventions.

**Results:**

Of 258 initially active participants, 225 (225/258 = 87.2%) successfully completed the online component and the top 200 were invited to the on-site workshop. Of those, 170 (170/200 = 85%) attended the on-site workshop. In total, 142 completed all three components, including the project phase. Quantitative and qualitative evaluation instruments included knowledge assessments, reflexive essays, and acceptability surveys. The mean pre and post-essay scores demonstrating understanding of social determinants, health system organization, and integration of HIV services were 70% and 87.5%, respectively, with an increase in knowledge of 17.2% (p<0.001). The mean pre- and post-test scores evaluating clinical knowledge were 70.9% and 90.3%, respectively, with an increase in knowledge of 19.4% (p<0.001). A survey of Likert scale and open-ended questions demonstrated overwhelming participant satisfaction with course content, structure, and effectiveness in improving their HIV-related knowledge and skills.

**Conclusion:**

This innovative curriculum utilized technology to target HCWs with limited access to educational resources. Participants benefited from technical skills acquired through the process, and could continue working within their underserved communities while participating in the online component and then implement interventions that successfully converted theoretical knowledge to action to improve integration of HIV care into primary care.

## Introduction

Central America, which consists of Belize, Costa Rica, El Salvador, Guatemala, Honduras, Nicaragua, and Panama, has been particularly affected by the rapidly spreading HIV epidemic in Latin America. Four of the countries with the highest prevalence rates in Latin America are in Central America and 200,000 people live with HIV/AIDS in the region [1]. Despite PAHO recommendations for universal HIV screening of pregnant women, only 52% of pregnant women were HIV-tested in Latin America and the Caribbean [2], [3] and only 36% of pregnant women diagnosed with HIV received antiretroviral therapy for the prevention of HIV mother-to-child transmission (PMTCT) in 2007 [3]. Lack of integration of HIV care into primary medical care in Central America leads to lost opportunities for HIV diagnosis and treatment, especially for rural or marginalized populations that may not have easy access to specialized HIV care at referral centers. This affects quality and access to care and long term prognosis of HIV-infected individuals.

Although guidelines are developed and discussed between national authorities and HIV experts, many training activities target doctors at specialized care clinics or hospitals [4], whereas training programs designed to facilitate the implementation of these recommendations by primary health care workers (HCWs) at the community level are limited. Many training programs fail to bridge the theoretical approach (e.g., National and International Guidelines and Protocols) to the realities on the ground, however this is becoming the focus of newly developed and implemented programs in other regions of the world [5]. In Central America, current strategies have traditionally targeted managers or higher-level officials, rather than local HCWs who have the majority of direct contact with patients. Furthermore, no training has been done on topics such as integration of health care, gender, and human rights, together with clinical tools to prevent transmission, all of which have a significant impact on final outcomes. An effective training program should train local HCWs on how to implement the guidelines and to empower them to identify barriers so that they may overcome them.

In order to develop an effective training program targeted directly to local HCWs in Central America, a government/academic partnership was formed between the Johns Hopkins Center for Clinical Global Health Education, Gorgas Memorial Institute in Panama, and the Pan American Health Organization (PAHO), with an evaluation component by the Epidemiological Research Center in Sexual and Reproductive Health in Guatemala (CIESAR by its name in Spanish) and funded by the US Department of Health and Human Services. Through this collaborative effort, we developed a novel 3-pronged blended educational strategy contextualizing theory into practical skills through an online distance learning component, followed by an on-site problem-solving workshop, and the development of intervention-action projects. The goal of this curriculum was to provide participants with the knowledge, skills, and attitudes necessary to improve the delivery of health services by integrating high-quality basic HIV and related infectious disease care with primary care. Our strategy was novel as it offered tutor-supported complementary online and on-site interactive components that translated knowledge into action and allowed us to specifically reach local HCWs in underserved communities with limited access to educational resources. In addition, the 8 week online component prepared students for the on-site training workshop by reinforcing fundamental concepts while avoiding prolonged absences from the workforce.

## Methods

### Ethics Statement

The Ministries of Health of each of the four participating countries (Guatemala, Nicaragua, Dominican Republic, and Panama) were approached. The baseline evaluation and the planned training program components and processes were described to the local authorities and a formal written approval was obtained to conduct the program with participants from their countries. Additionally, the Ministries of Health provided formal written statements that approval by their national bioethics committees was not needed, as this was an educational activity and the evaluation activities posed no risk to participants. The Johns Hopkins Medicine Institutional Review Boards approved the analysis of the data from the training program (NA_00075375), which qualified for exemption as not human subjects research. The analysis used program evaluation activities to examine the overall effectiveness, feasibility, and acceptability of the educational program, and posed no risk to participants. Written consent was obtained for all surveys in the baseline evaluation, but was not required for the anonymously analyzed data from the course evaluation activities.

### Needs Assessment and Baseline Evaluation

A general needs assessment consisting of interviews with regional leaders, content experts, and community stakeholders, and a review of local and regional data from country reports [6]–[9] led to the development of the curriculum and the selection of participants from underserved communities. Although there was a focus on underserved communities that had some common factors, establishing a homogeneous definition for these communities was not possible. Included in the assessment were reports addressing gaps in human resources for the health sector in the Americas and the main objectives of the region that needed to be addressed, including more specific needs related to the prevention of HIV mother-to-child transmission (PMTCT) [2], [10], [11].

CIESAR carried out a baseline evaluation of HCWs, administrators, and patients in the selected regions. This evaluation set out to determine Knowledge, Attitudes and Practices (KAP) regarding the integration of HIV and other infectious diseases into primary health care. Three different questionnaires were directed at i) physicians, nurses and health educators, ii) directors of selected health services facilities, and iii) health service users (outpatients) in the targeted areas. The questionnaires were validated and adjusted in each participating country with changes to adapt for local language.

### Setting and Participants

Following the results of the needs assessment, the training program was deployed in Guatemala, Nicaragua, the host country of Panama, and the Dominican Republic in the Caribbean. At the time of the development of the program, other countries were considered based on HIV prevalence but were ruled out due to either political instability at the time (Honduras) or language (Belize). The focus was on HCWs (doctors, nurses, community health promoters, psychologists, health administrators, etc.) who work directly in primary care in underserved areas with limited access to health services and limited access to traditional onsite training. Participants were nominated by the national health authorities of their countries following specific selection criteria: 1) operational staff located in the area selected, 2) permanent contract staff, 3) staff enthusiasm and commitment to participate, 4) potential to assist in replication of course, and 5) basic computer knowledge.

### Goals and Objectives

The main goal of the training program entitled “Integration of HIV and other prevalent infectious disease care into primary medical care” was to strengthen the knowledge and skills of health workers in Latin America and the Caribbean to integrate HIV and other infectious disease care into primary care, with a special emphasis in PMTCT. A competency-based curriculum was developed aimed at not only improving knowledge, but also modifying attitudes and developing abilities that could be measured as outcomes of the course [12]. The defined competencies included: i) to integrate tools to facilitate the promotion, prevention, diagnosis, and treatment of HIV, particularly in PMTCT, congenital syphilis, and other associated infectious diseases; ii) to manage clinical tools to improve service delivery and adherence of persons with HIV and other prevalent infectious diseases; and iii) to develop proposals for sustainable interventions, including training, to improve the efficiency of care.

### Educational Strategies

**Figure 1:**
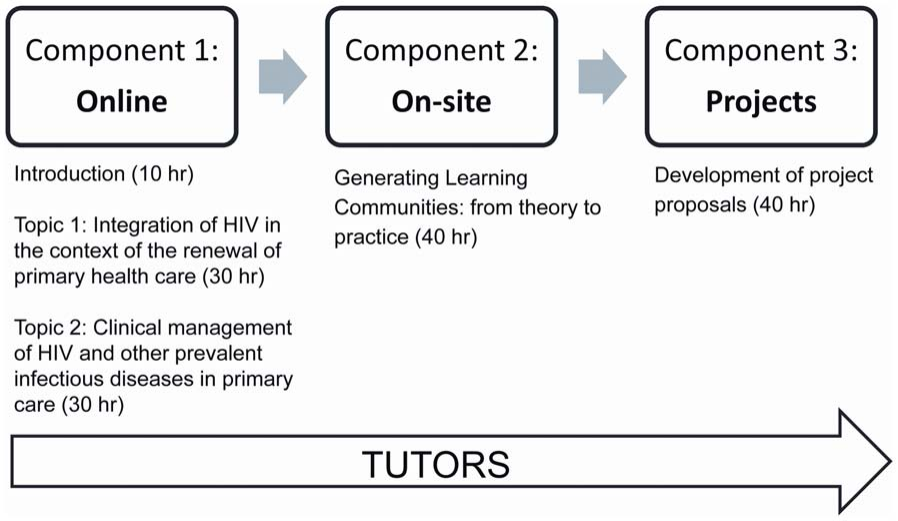
Educational Strategy. The curriculum focused on principles of HIV care and health systems using a tutor-supported blended educational approach of an 8-week online component, a weeklong on-site problem-solving workshop, and individualized project-based interventions.

The competency-based curriculum for this training program focused on principles of HIV care and health systems using a novel blended educational strategy consisting of 150 hours of activities grouped in 3 components: an 8-week online component (Component 1), a weeklong on-site problem-solving workshop (Component 2), and individualized project-based interventions (Component 3) (Figure 1). The online component allowed HCWs to continue working in their underserved communities while in training. The concepts covered in the online component would prepare participants to fully participate in problem-solving workshops during the on-site component. The on-site workshops reinforced and contextualized to their local setting the concepts that were covered in the online component, and prepared participants for the development of project proposals. The final phase consisted of developing proposals for intervention-action projects that could be implemented in their local communities or clinics to improve integration of HIV services. Throughout all three components, trained tutors evaluated and mentored participants by assisting with technological issues, answering questions on assignments, and providing guidance on proposal development (Figure 1).

### Implementation

#### Tutor selection and training

Twenty-six potential tutors working at academic institutions were nominated by national health authorities in each country to attend a five day workshop in Panama where they were trained in competency-based education. The workshop focused on educational methodologies, an overview of program topics, and operation of the program, while allowing evaluation and selection of the final 24 tutors for the program. The selected tutors were given a manual outlining their roles in the training program (Supporting Information S2), and were further trained during the on-site component to support the final project component. In total, the tutors received 65 hours of training. The selected tutors were a mix of doctors, nurses, and public health experts with extensive experience in education as professors or tutors in universities or administrators of academic programs, and several had experience in virtual education.

#### Component 1: online

The training program began with an 8 week online component consisting of 21 recorded lectures on *Topic 1: Integration of HIV in the context of the renewal of primary health care* focusing on general concepts of health politics, rights, and systems in the region and 22 lectures on *Topic 2: Clinical management of HIV and other prevalent infectious diseases in primary care* (Figure 1 and Supporting Information S3). The online component was run on a free, open-source learning management system, Moodle, on the Virtual Campus of Public Health organized by PAHO (www.campusvirtualsp.org). The online component included weekly evaluations, assignments, and forums where participants could ask questions and interact with other participants. Lectures provided by multiple international experts in HIV and health care systems were prerecorded using Mediasite multimedia capturing software by Sonic Foundry and available on-demand for participants on the program website. In addition to the recordings, transcriptions of the lectures and handouts of the presentation slides were also available from the program site. To ensure access to the program materials regardless of internet connectivity, CD-ROMS with lecture material were delivered to all participants. In order to obtain baseline information on the participants who enrolled in the online component, a brief registration survey was conducted using SurveyMonkey [13].

#### Component 2: on-site

The second component was an on-site problem-solving workshop in Panama. The 200 top-scoring participants from the online component were invited to participate in the on-site component. Participants spent five 8-hour days in workshops tailored to reinforce and apply concepts they had learned during the online component. These workshops included clinical cases, role playing and group work to outline the relationship between HCW and patient, the effective transfer of information, and changes in provider attitude that can reflect in patient care and potentially lead to changes in patient conduct (Figure 1 and Supporting Information S3). Throughout the workshops, participants focused on developing ideas for feasible intervention-action projects applicable to their local settings that would be developed further in the final component.

#### Component 3: project proposals

The final phase of this program was the Project component in which participants had two months to develop individualized proposals for a local intervention-action project that could contribute to integration of HIV services. Participants received format and content guidelines, and were required to include a detailed timeline and budget for their project. Participants defended their proposals with 10 minute presentations in national workshops in front of their peers, and academic and government representatives.

### Evaluation and Feedback

Quantitative and qualitative evaluation instruments were used to evaluate student progress during the online component. Participants wrote reflexive essays at the beginning and end of the online component which qualitatively measured understanding of social determinants, health care system organization, and integration of HIV services. Additionally, participants had to write weekly small essays during Topic 1 (*Integration of HIV*) to identify barriers to care, identify and analyze the organization of the provision of care in her/his health center, and to propose changes to improve effective care. Clinical knowledge in the online component was tested using multiple-choice pre and post-tests for each module in Topic 2 (*Clinical Management of HIV*). Two surveys were administered to obtain participant feedback: the first evaluated the online component exclusively, and the second evaluated the entire program at the completion of all three components. The feedback surveys used Likert scale from 1 to 5 (1 = strongly agree to 5 = strongly disagree), multiple choice, and open-ended questions to evaluate overall program satisfaction, acceptability of the online component, and perceived benefit from the training program. These surveys were completed using Survey Monkey [13] and are available as Supporting Information S4 and S5.

### Data Collection and Analysis

Data from the four countries for the baseline evaluation was entered into and analyzed with the EPI INFO software DOS version 6.04d [14]. The scores for essays and pre- and post-tests were managed and descriptive statistics performed using Microsoft Excel (2007). Mean percentages and paired t-tests were performed to determine if there was an increase in knowledge following lectures given in Topic 2 of the online component (pre- and post-tests) and after completing the online component (pre- and post-reflexive essays).

## Results

### Needs Assessment and Baseline Evaluation

A large need was identified in HIV testing, particularly among pregnant women with only 52% of pregnant women being tested for HIV in the Latin America and Carribean (LAC) region, and even lower rates in the selected countries (Dominican Republic, 42%; Nicaragua, 20%; and Guatemala, 10%) [2], [3]. Additionally, only 36% of pregnant women in LAC who were diagnosed with HIV received antiretroviral prophylaxis for PMTCT in 2007 [2], [3].

For the baseline evaluation, CIESAR completed a total of 1,735 interviews which were carried out across the four countries (750 with health care providers, 44 with directors of health facilities, and 941 with health service users).The results of the three questionnaires in the baseline evaluation pointed out discrepancies in the perception of integration of HIV services. Results indicated that 33/44 (75%) of directors from all four countries reported that their health facility offered HIV tests for the general at risk population, whereas only 427/750 (57%) of health providers reported offering the test and 541/941 (57%) of outpatients reported having been tested. When looking at individual countries, Nicaragua showed the largest discrepancy with 105/113 (93%) of providers reporting that they offer HIV tests, but only 104/190 (55%) of outpatients reporting they have been tested. While 95% and 89% of directors and health service providers, respectively, affirmed that HIV testing was routinely offered during prenatal care in their health facilities, only 423/650 (65%) of the women reporting they were or had been pregnant stated they had been tested for HIV. This difference was statistically significant in each of the countries except for Panama.

### Process Indicators

Of the 269 participants who were initially enrolled by the Ministries of Health, 258 became active participants by completing one or more of the initial activities (e.g., creating a profile, completing the registration survey). As the online component progressed, thirty-three participants were dropped due to inactivity, resulting in 225 (87.2%) participants that completed the online component. Only 2 of the 225 participants who completed the online component did not receive the required score (70%) to pass this component. Due to logistical restraints, only the top-scoring 200 participants were invited to the onsite workshop and 30 of those could not attend due to lack of government support from one of the countries. The training program concluded with the presentation of 152 project proposals in the 4 countries, some (10) from participants who were not able to attend the onsite workshop. In total, 142 participants completed all three components.

### Characteristics of Course Participants

At the initiation of the online component, 240/258 (93%) of the initially active participants completed an initial registration survey (Table 1). The majority of our participants were female, over 25 years old, and working in the public sector. Approximately half of the participants were medical doctors in primary care (general medicine, family medicine, pediatrics, and OB/GYN). Non-physician participants included nurses, clinic administrators, community health promoters, and social workers. Over half the participants had been in their profession more than ten years. Most participants worked in mid-size clinical settings (100–1000 patients per month), but about one fourth worked in small clinics (<100 patients per month) and one fourth in large hospital settings with >1000 patients per month. A larger proportion of participants from Panama reported working in large hospitals, whereas the participants in Nicaragua worked mainly in small clinics and participants from the Dominican Republic worked in mid-sizes clinics. The majority of participants in Guatemala were split between small clinics and very large hospitals. The majority of participants reported modest experience seeing patients with HIV or pregnant women. Most participants had a computer with internet access at home or at work, but 12.7% only had access at local internet cafés. Two-thirds of our participants had daily access to internet, a quarter only a few times a week, and approximately 10% weekly or less (Table 1).

**Table 1:** Participant Characteristics.

CHARACTERISTICS	RESPONSE	%
**Participant Demographics**		
**Gender (n = 227)** a	Male	26.0
	Female	**74.0**
**Age (n = 239)**	18–25	3.3
	26–35	32.6
	36–45	**33.1**
	46+	30.9
**Participant Profession Characteristics**		
**Profession (n = 240)** b	MD	**54.2**
	Registered nurse	26.7
	Clinic administrator	8.3
	Other^c^	16.2
**Specialty (n = 210)** b	General medicine	**54.3**
	Family medicine	9.0
	Infectious disease	8.1
	OB/GYN	6.7
	Pediatrics	4.3
	Other	25.7
**Years in profession (n = 237)**	≤5 yrs	25.3
	6–10 yrs	19.8
	11–15 yrs	15.6
	>15 yrs	**39.2**
**Industry of practice (n = 239)** b	Public	**98.3**
	Social Security	2.5
	Private	11.7
**Patient Demographics**		
**# of patients seen per month at clinic/hospital where participant works (n = 181)**	0–100	23.2
	101–1000	**53.6**
	>1000	23.2
**% of participant’s patients that are HIV+ (n = 170)**	0	18.8
	1–20	**68.2**
	>20	13
**% of participant’s patients that are pregnant women (n = 175)**	0	22.9
	1–20	**53.1**
	>20	19.5
**Participant Access to Technology**		
**Location where participant can access a computer with internet connection (n = 237)** b	Home	**75.1**
	Work	46.4
	Internet café	29.1
	Other^d^	6.0
**Frequency of access to Internet (n = 227)**	At least once a day	**62.6**
	A few times a week	25.1
	Once a week or less	12.3

a Some questions were skipped by participants. The total number of participants who answered a question is displayed next to the characteristic.

b Participant could select more than one option.

c Includes social workers, health promoters, dentists/dental surgeons, psychologists, lab technicians, pharmacists, a nutritionist, health economist, professor of primary education, data entry specialist for the early warning system of HIV/TB, and participants with degrees in nursing, public health, and science.

d Includes cell phone, home of friend or family, and husband’s work place, library.

### Evaluations

#### Reflexive essays

All available reflexive essay pairs (pre- and post-online component, n = 209) from the online component were evaluated by tutors using a predetermined checklist of criteria qualitatively measuring understanding of social determinants, health care system organization, and integration of HIV services. The mean pre-essay score was 70.3% (95% CI 68.0, 72.6) and the mean post-essay score was 87.5% (95% CI 86.3, 88.7). The mean difference between scores for paired pre- and post-essays was 17.2% (95% CI 15.4–19.0, p<0.001).

#### Multiple-choice pre- and post-tests

Scores on the pre- and post-tests from Topic 2 (*Clinical Management of HIV*) evaluating clinical knowledge were analyzed for the 225 participants who completed the online component. The mean pre-test score was 70.9% (95% CI 68.7, 73.1), and the mean post-test score was 90.3% (95% CI 88.9, 91.7). The mean difference between scores for the paired pre- and post-test knowledge assessments was 19.4% (95% CI 17.4–21.4, p<0.001).

#### Participant feedback: online component

Of the 225 participants who completed the online component, 223 (99%) completed a feedback survey evaluating content, structure, tools, tutors, and evaluations of the online component. The participants highly rated structure of the online component including clarity of objectives and appropriate number of lectures (1.36), educational tools and materials including recordings of lectures, handouts, transcripts, and CD-ROMS (1.3), and utility of content for integrating HIV into primary care (1.1).

Ratings for tutors were consistently agreeable with an average score of 1.28. Participants highly rated the tutors stating that they facilitated the general process (1.21) and the technical aspects (1.25) of the online component, and the overall process of learning (1.25). They also reported that the number of participants per tutor was adequate (1.59) and that the tutors responded to questions in a timely manner and with correct information (1.22). Overall, participants believed that their tutor was fundamental in completing the online component (1.22).

#### Participant feedback: overall program

While the majority of participants agreed that the online component prepared them for the onsite component, only one fifth (19%) believed that the online component alone would have been sufficient to acquire the knowledge to integrate HIV services. Most participants (85%) agreed that the on-site component and project development improved their ability to apply the theoretical knowledge acquired in the online component to their practice. Participants highly rated the online component (1.16), on-site component (1.15), and the development of project proposals (1.43).

Overwhelmingly, the two most important factors that caused difficulties in completing the program were time commitment in fulfilling their normal responsibilities in the clinics while also completing the online component and availability/reliability of the internet connection. However, participants reported substantial improvement in computer literacy; self-reported ability to perform tasks in a computer with ease (excellent skill) doubled during the course of the training program (Table 2). In open-ended questions, participants mentioned that they benefitted from interaction with participants from different countries during the onsite workshop. They also believed that participation in this training program led to the formation of transnational learning communities that could continue the push to integrate HIV services.

**Table 2:** Computer skills obtained.

COMPUTER SKILL	NUMBER (%) WITH “EXCELLENT” SKILL BEFORE THE COURSE^a^	NUMBER (%) WITH “EXCELLENT” SKILL AFTER THE COURSE	CHI SQUARE P-VALUE
**Opening files**	37 (28)	81 (61.4)	<0.001
**Email**	42 (31.8)	85 (65.9)	<0.001
**Opening attachments**	32 (24.2)	79 (59.8)	<0.001
**Connecting to internet**	39 (30.0)	85 (64.9)	<0.001
**Navigating the internet (internet searches)**	36 (27.1)	78 (59.5)	<0.001
**Download/upload files**	31 (23.8)	77 (59.2)	<0.001

a Participants rated their computer skills before and after the course. The number of participants who rated their skills as “Excellent” is shown here (scale: “Excellent”, “Very good”, “Good”, “Regular”, “Deficient”).

### Project Proposals

In total, 152 participant-developed project proposals from the four countries were presented: 131 in three countries in national workshops and 21 to course coordinators and academic professionals through web-based conferencing. Projects included baseline knowledge surveys and needs assessments (clinic, community, etc.,), capacity building of health workers, community education programs, reviews of clinic procedures and regulatory compliance, and evaluation of existing programs. Topics included preventing HIV mother-to-child transmission, sexual and reproductive health, family planning, stigma, testing and counseling, and adherence. Although there was a difference in complexity of the proposed projects from different countries, they were all small and focused in scope with an average budget of $8500, providing realistic approaches to health officials for achieving integration. Following the project presentations in the national workshops, the USAID put forth a proposal to fund one of the projects from Panama and the PAHO office in the Dominican Republic has set aside $12,000 to fund several projects in their country. Additionally, reports from a few participants in Guatemala stated that several projects had been implemented or were in the process of being implemented at the time of this publication.

## Discussion

Internet-based education has become increasingly popular for medical education [15]–[17]. However, few programs target community HCWs at the primary level of care, the main players in resource-limited settings, and none, that we have found, use this novel tutor-supported 3-pronged blended educational strategy which offered complementary online and on-site interactive components based on competencies[18]–[20]. Despite a demanding workload and with the help of in-country tutors, our program achieved high retention, was well received, and developed knowledge and skills needed to integrate HIV services in primary care.

Achieving high retention rates is a goal of all educational programs, but in internet-based courses retention rates are typically low [21]–[23], sometimes with dropout rates 10 to 20 percent higher in distance education than in traditional courses [24]. Notably, our training program achieved a high retention rate (87.2%) in the online component. We believe several factors were involved in achieving this high retention rate. First, our training program offered participants an incentive to successfully complete the online component: the top 200 participants who completed the online component would be invited to participate in the on-site component in Panama. Many participants had never left their country and to take part in this intensive on-site workshop was a great achievement for them. Although this program could have been limited to different areas in a single country, participants reported that a particularly beneficial and unique aspect of the on-site component was the opportunity to connect with participants from other countries, exchange ideas, and learn from the health care systems in settings different to their own. Their combined experiences led to a better understanding of how different services could be integrated and to the development of realistic and innovative project proposals.

The lack of human connection in internet-based courses is a major factor leading to low retention rates [15], [21], [22], [24]. The sense of isolation was minimized in our training program due to various factors. As participants were nominated by the national health authorities in selected areas of their countries, workers from the same region participating in the training program formed small clusters of participants that could work together face-to-face and motivate each other. In-country tutors maintained the human connection with their assigned participants by being in constant contact with them by phone, e-mail, or internet and some even organized small face-to-face study groups for their participants. Additionally, partnering with local government and health ministry representatives provided an extra mechanism to reach out to participants when tutors were unable to obtain a response from the participants. The addition of tutors in the program to support the participants was an essential factor to the success of the program. A recurrent theme that emerged from focus group conversations and open ended feedback questions was the importance of the tutors in facilitating the course. The tutors served as mentors and advocates, ensuring completion of assignments according to schedule, identifying barriers to completion due to knowledge gaps, lack of connectivity, or limited computer literacy, and bridging the communication between participants and course coordinators. They also initiated discussions with their participants to relate the course material to their practices and clinics, and assisted them in developing feasible intervention action projects to complete the program.

Another potential factor in the high retention rate was the feasibility, acceptability, and applicability of the program which was highly valued by our participants. For most participants working in underserved communities with limited access to educational resources, taking time off work to attend training workshops outside of their communities was unrealistic, much like health care providers in general [15], [25]. Our training program provided an opportunity to obtain the bulk of training online in their own setting, preparing them for a much shorter, intense face-to-face workshop where they could fully interact and participate with the knowledge they had learned in the online component while minimizing the time away from their clinics. As a result of the needs assessment, the program content taught practical knowledge and skills that the participants felt were applicable to their setting, and therefore, participants were more likely to continue and complete the program.

The program aimed to address the deficiencies in knowledge, lack of integration, and pervasive stigma identified in health providers at the primary level of care. We found that this intervention was highly effective in building both the knowledge and skills needed for the health care workers to integrate HIV into primary care. The majority of our participants felt that the online component alone would have been insufficient to acquire the knowledge, attitudes, and skills necessary to integrate HIV services, suggesting that the blended approach of online, onsite, and project components played a major role in making this program successful. As participants presented their projects, it was apparent that they not only acquired knowledge and skills that will help them in their present work, but they have learned how to search for answers to questions and to develop and research new ideas.

Throughout the process of developing and implementing this training program, our multi-partner team learned several important lessons. Although technology was a useful tool to reach the most underserved areas, it was also a bigger barrier than expected [15], [26]. While reliable internet connectivity was an obstacle, content on the CD-ROMS allowed participants to continue their coursework with limited access to the internet. Additionally, our program specifically targeted HCWs in the trenches of care in resource-limited settings, who are often excluded from multinational training initiatives. Because of the emphasis in reaching HCW working in underserved areas, computer literacy was intentionally not taken into consideration in participant selection. In such a scenario, the tutors became of paramount importance, teaching highly motivated participants how to enter the online classroom and perform the basic functions of uploading and downloading files, email, and discussion forums. As such, an unanticipated benefit of the program was the reported improvements in computer literacy gained by the participants as a result of the program (Table 2).

For many participants, completion of this program was a personal achievement that empowered them to integrate HIV care into their practice and identify barriers to optimal care so that they may overcome them. As participants advanced through the program, they formed new learning communities among their peers locally and internationally that have the potential to sustain the momentum of integrating HIV services into primary care in the long term. Furthermore, partnering with local government, academic, and health ministry representatives throughout the program was also very important in providing participants a channel to make a difference in their communities. Particularly, the final presentation of their project proposals in front of these higher level officials, gave them the opportunity to present their work to people who have the resources to help them implement their projects. This requirement served as an additional learning experience and as an incentive to complete the program as they presented to potential funders.

Despite the success of the program and the positive feedback from the participants, there were limitations, and several elements could be improved. An introductory interactive module to familiarize participants with basic computer functions and how to navigate the course more effectively was available to participants but not required, and because of delays at the beginning of the course, few participants completed this introductory module. Future deployments of this training program should require this introductory module to assist in the smooth operation of the online component. Limitations to internet connectivity in these underserved areas also added undue stress for some participants. Potentially, a minimal amount of funding could be obtained to provide a central location where participants could access the internet; provide a small stipend for internet cafes or to purchase personal mobile hotspots, which several of our highly motivated participants did with their own funds; or the use of smart phones given that cell phone use and connectivity may be more common than internet. Additionally, future deployments should establish the infrastructure needed to finance the small-scale intervention projects which have the potential to contribute to national health programs by implementing local effective and feasible strategies to integrate HIV care into primary care [27]. Evaluation of competency-based education programs is complex [28], [29], however our training program used a variety of assessment tools in order to overcome limitations of any individual tool. Further limitations of our study can be found in the lack of standardization of our evaluation tools, including the pre- and post-tests and participant feedback surveys, and the subjectivity in the grading of the reflexive essays by different tutors, although this was minimized by the use of a checklist of criteria and there was no significant difference in the scores of participants from different countries. Notably, composition of the essays was an additional unexpected challenge for some participants in terms of deficiencies in spelling and syntax, but tutors worked with those participants to improve their writing skills. Finally, our study lacks long term health outcomes data that would be beneficial as an evaluation of the program. Additional funding for a long term evaluation will provide useful information on the impact of the training program on integration of HIV services, clinical practice, and patient outcomes. The baseline evaluation and final essays contained valuable information that could be used as a form of local assessment by health authorities and to adapt future deployments of the program to specific countries. Ongoing efforts in the region, such as a subregional workshop in El Salvador on the *Development of Human Resource Competencies* which used this training program as a model, the development of a self-paced version of the online component (available at www.ccghe.net), and the development of self-contained Learning Modules (Supporting Information S6, S7, S8, S9, S10, S11, S12, S13) aim to ensure long-term sustainability of this training program.

### Conclusions

This innovative curriculum utilized technology to target HCWs with limited access to educational resources. The novel 3-pronged blended educational strategy successfully impacted capacity to convert theoretical knowledge to action with its competency-based curriculum that achieved our goal of providing participants with the knowledge, skills, and attitudes necessary to improve the delivery of health services by integrating HIV care with primary care. Our program achieved a high retention rate with the support from tutors who provided the needed human connection that is usually lacking in distance education. Initially, technology proved to be a barrier but participants benefited from technical skills acquired through the process and they were able to continue working in their underserved communities while participating in the online component. They could then learn how to apply their newly acquired knowledge in problem-solving workshops, and propose practical interventions to improve integration of HIV care into primary care. More investment in training programs for underserved areas and future deployments of similar training programs in different countries could make a significant impact in improving HIV care and access to health care services by building the capacity of local HCWs.

## Supporting Information

Supporting Information S1 **Alternative Language Abstract.** Spanish
(DOCX)

Supporting Information S2 **Manual for Tutors.** This manual was created for the tutors to assist in their training and to describe their roles and responsibilities throughout the different phases of the program. This manual also serves as a detailed description of the program that others can use in order to replicate the program.
(DOC)

Supporting Information S3 **Components and breakdown of the training program.** Topics covered, length, and modality used for each component.
(PDF)

Supporting Information S4 **Online Component Participant Feedback Survey.** This survey was constructed and delivered using SurveyMonkey [13] and participants took the survey at the conclusion of the online component.
(PDF)

Supporting Information S5 **Overall Program Participant Feedback Survey.** This survey was constructed and delivered using SurveyMonkey [13] and participants took the survey after completing all three components.
(PDF)

Supporting Information S6 **Learning Module 1 - HIV and the Social Dimension of Health.** Each Learning Module includes a sample of 2 video conferences, a self-assessment (quiz), and complementary materials that were originally presented in the online component of the program. Learning Module 1 includes video conferences on “Epidemiology of HIV in Latin America” and “The Social Dimension of Health: The Social Determinants”.
(RAR)

Supporting Information S7 **Learning Module 2 - HIV, health systems and programs.** Each Learning Module includes a sample of 2 video conferences, a self-assessment (quiz), and complementary materials that were originally presented in the online component of the program. Learning Module 2 includes video conferences on “A Comprehensive and Integrated Approach” and “Basic Health Programs: Healthy Life Cycle”.
(RAR)

Supporting Information S8 **Learning Module 3 - Managing Change for the Integration of HIV into Primary Care.** Each Learning Module includes a sample of 2 video conferences, a self-assessment (quiz), and complementary materials that were originally presented in the online component of the program. Learning Module 3 includes video conferences on “Change Management for the Renewal of Primary Health Care” and “Community Participation: Intersectoriality”.
(RAR)

Supporting Information S9 **Learning Module 4 - Integration of HIV into Primary Care.** Each Learning Module includes a sample of 2 video conferences, a self-assessment (quiz), and complementary materials that were originally presented in the online component of the program. Learning Module 4 includes video conferences on “Models of Integration of HIV Services” and “HIV Prevention in Adolescents and Youth”.
(RAR)

Supporting Information S10 **Learning Module 5 - Initial Management of HIV.** Each Learning Module includes a sample of 2 video conferences, a self-assessment (quiz), and complementary materials that were originally presented in the online component of the program. Learning Module 5 includes video conferences on “Mechanisms of Transmission and Natural History of HIV Infection” and “HIV Counseling and Testing”.
(RAR)

Supporting Information S11 **Learning Module 6 - HIV in pregnant women.** Each Learning Module includes a sample of 2 video conferences, a self-assessment (quiz), and complementary materials that were originally presented in the online component of the program. Learning Module 6 includes video conferences on “HIV Testing in Prenatal Care” and “Management of Delivery and the Exposed Infant”.
(RAR)

Supporting Information S12 **Learning Module 7 - Chronic Management of HIV.** Each Learning Module includes a sample of 2 video conferences, a self-assessment (quiz), and complementary materials that were originally presented in the online component of the program. Learning Module 7 includes video conferences on “Tuberculosis and HIV Coinfection” and “Toxicity, Adherence, and Laboratory Monitoring”.
(RAR)

Supporting Information S13 **Learning Object 8: Prevalent Infections and HIV.** Each Learning Module includes a sample of 2 video conferences, a self-assessment (quiz), and complementary materials that were originally presented in the online component of the program. Learning Module 8 includes video conferences on “Hepatitis and HIV Coinfection” and “Parasites in Patients with HIV/AIDS”.
(RAR)
