# Correction: A Novel Educational Strategy Targeting Health Care Workers in Underserved Communities in Central America to Integrate HIV into Primary Medical Care

**DOI:** 10.1371/annotation/0e024b1f-cfcc-444d-836c-6fd97a6169c1

**Published:** 2013-07-30

**Authors:** Tamara Flys, Rosalba González, Omar Sued, Juana Suarez Conejero, Edgar Kestler, Nestor Sosa, Jane McKenzie-White, Irma Irene Monzón, Carmen-Rosa Torres, Kathleen Page

Supporting Information files S2-S5 are linked to the incorrect files. The legends are correct. Please use the following links to access the proper Supporting Information files:

S2: 

S3: 

S4: 

S5: 
