# Supplementary material for: A Novel Educational Strategy Targeting Health Care Workers in Underserved Communities in Central America to Integrate HIV into Primary Medical Care
Source: PLoS One. 2012 Oct 24;7(10):e46426. doi: 10.1371/journal.pone.0046426 (PMC3480350; doi:10.1371/journal.pone.0046426)
Supplement: Supporting Information S1 — Alternative Language Abstract. Spanish (DOCX) [file pone.0046426.s001.docx]

**Una nueva estrategia educativa dirigida a trabajadores de la salud de comunidades marginadas en América Central para integrar el VIH en la atención primaria.**

**Antecedentes**: Las estrategias actuales educativas para integrar el VIH en la atención primaria en América Central han estado dirigidas, tradicionalmente, a los administradores o funcionarios de alto nivel, en lugar de a los trabajadores locales de salud. Hemos desarrollado un programa de formación interactiva en línea y presencial para llegar a los trabajadores de salud locales del primer nivel de atención en comunidades marginadas.

**Método**: El programa fue dirigido a médicos, enfermeras y personal de salud de comunidades con acceso limitado a la capacitación presencial tradicional en cuatro países: Panamá, Nicaragua, República Dominicana y Guatemala. El plan de estudios se centró en los principios de la atención del VIH y los sistemas de salud, usando un enfoque educativo multimodal (presencial y virtual) y dividido en tres componentes donde los participantes estuvieron acompañados por tutores. El primer componente fue el virtual que duró 8 semanas. El segundo consistió en una semana de talleres presenciales enfocados a la resolución de problemas, y finalmente, en el tercer componente se elaboraron intervenciones basadas en proyectos individuales.

**Resultados**: De los 258 participantes inicialmente activos, 225 (225/258 = 87,2%) completaron el componente en línea y los mejores 200 fueron invitados a los talleres presenciales. De ellos, 170 (170/200 = 85%) asistieron. En total, 142 completaron los tres componentes, incluyendo la fase de proyectos. Instrumentos de evaluación cuantitativos y cualitativos incluyeron evaluaciones de los conocimientos, ensayos reflexivos, y las encuestas de aceptabilidad. Las puntuaciones medias de los ensayos pre y post demostraron la comprensión de los determinantes sociales, la organización de los sistemas de salud, y la integración de los servicios de VIH y fueron del 70% y el 87,5% respectivamente, con un aumento en el conocimiento del 17,2% (p <0,001). Las puntuaciones medias de las evaluaciones pre y post que evaluaron el conocimiento clínico fueron 70.9% y 90.3% respectivamente, con un aumento en el conocimiento del 19,4% (p <0,001). Una encuesta realizada que aplicó la escala Likert y preguntas abiertas demostró la satisfacción de los participantes con el contenido del curso, la estructura y la eficacia en la mejora de los conocimientos y habilidades sobre el VIH.

**Conclusiones**: Este programa innovador utilizó la tecnología para incluir a trabajadores de salud que tenían acceso limitado a recursos y programas educativos. Los participantes se beneficiaron de las habilidades técnicas y tecnológicas adquiridas a través del proceso, y pudieron seguir trabajando dentro de sus comunidades durante su participación, lo que les posibilitó implementar las intervenciones en su entorno inmediato, hecho que vinculó con éxito el conocimiento teórico adquirido con la acción, y que permitió mejorar la integración del VIH en la atención primaria.
