# Supplementary material for: A Novel Educational Strategy Targeting Health Care Workers in Underserved Communities in Central America to Integrate HIV into Primary Medical Care
Source: PLoS One. 2012 Oct 24;7(10):e46426. doi: 10.1371/journal.pone.0046426 (PMC3480350; doi:10.1371/journal.pone.0046426)
Supplement: Supporting Information S2 — Manual for Tutors. This manual was created for the tutors to assist in their training and to describe their roles and responsibilities throughout the different phases of the program. This manual also serves as a detailed description of the program that others can use in order to replicate the program. (DOC) [file pone.0046426.s002.doc]

**Supporting Information S2. Components and breakdown of the training program**

| **COMPONENT** | **TOPIC** | **TOPICS COVERED** | **HOURS** | **MODALITY** |
| --- | --- | --- | --- | --- |
|  | **Introduction** |  | 10 | Online |
| **COMPONENT 1** | **Topic 1: Integration of HIV in the context of the renewal of primary health care** | *Week 1*: Social determinants of health, human rights, and HIV | 30 | Online |
|  |  | *Week 2*: Health systems and their impact in the response to HIV and prevalent infectious diseases |  |  |
|  |  | *Week 3*: Renovation of primary health care |  |  |
|  |  | *Week 4*: Models of integration of HIV and other prevalent infectious diseases |  |  |
|  | **Topic 2: Clinical management of HIV and other prevalent infectious diseases in primary care** | *Week 1*: Initial management of HIV+ patients | 30 | Online |
|  |  | *Week 2*: HIV in pregnant women |  |  |
|  |  | *Week 3*: Opportunistic infections and chronic management of HIV |  |  |
|  |  | *Week 4*: Other prevalent infections. Individual and community interventions for prevention and control of vector-transmitted infections |  |  |
| **COMPONENT 2** | **Generating Learning Communities: from theory to practice** | Primary Care | 40 | On-site |
|  |  | eHealth |  |  |
|  |  | Sexual and Reproductive Health |  |  |
|  |  | Provider-initiated HIV testing and counseling |  |  |
|  |  | HIV Treatment |  |  |
|  |  | HIV-associated diseases |  |  |
|  |  | Health promotion and prevention |  |  |
|  |  | HIV+ pregnant women and congenital syphilis |  |  |
|  |  | Research methodology |  |  |
| **COMPONENT 3** | **Projects** |  | 40 | Partial On-site |
|  | **TOTAL** |  | 150 |  |
