# Supplementary material for: A Novel Educational Strategy Targeting Health Care Workers in Underserved Communities in Central America to Integrate HIV into Primary Medical Care
Source: PLoS One. 2012 Oct 24;7(10):e46426. doi: 10.1371/journal.pone.0046426 (PMC3480350; doi:10.1371/journal.pone.0046426)
Supplement: Supporting Information S3 — Components and breakdown of the training program. Topics covered, length, and modality used for each component. (PDF) [file pone.0046426.s003.pdf]

HIV training program - Online component participant feedback

1. Felicitaciones por haber completado la parte virtual de este curso

Estimado/a participante,

Gracias por su participación en este curso virtual "Integración del VIH y Otras Enfermedades Infecciosas Prevalentes en el Primer Nivel de Atención".

Se tomará unos 5-10 minutos para completar esta breve evaluación de curso que está diseñada para proporcionar información a los directores del curso y los docentes a cerca su experiencia con este programa de capacitación. Agradecemos sus opiniones francas y honestas a cerca de que funcionó bien y que no funcionó bien, con el fin de ayudarnos a revisar y optimizar cursos para el futuro. Para promover su aportación constructiva y honesta, le queremos avisar que comunicaremos sus opiniones a los docentes a través de un resumen que no se asociará con su nombre.

La realización de esta evaluación es un requisito para recibir el certificado oficial final. Le pedimos su nombre en la siguiente página únicamente para estar al tanto de quien ha terminado la evaluación y para proporcionarle su certificado. Como indicamos anteriormente, no se va a asociar su nombre con los comentarios.

Una vez más, muchas gracias por su participación en este curso y le agradecemos su ayuda con esta evaluación.

Saludos,

Equipo docente

2. Su experiencia con este curso virtual

*1. Por favor, escriba su nombre aquí.

2. En cuanto a la estructura del curso virtual,

	Totalmente de acuerdo	De acuerdo	No tengo opinión	En desacuerdo	Totalmente en desacuerdo
Los objetivos del curso definieron claramente el contenido del curso y las expectativas	○	○	○	○	○
El orden de las conferencias fue apropiado	○	○	○	○	○
La duración de las conferencias fue adecuada	○	○	○	○	○
El número de conferencias fue apropiado	○	○	○	○	○
El curso se organizó de una manera que facilitó el aprendizaje	○	○	○	○	○

Otros comentarios sobre la forma en que se estructuró el curso

3. Por favor evalúe el contenido de esta parte virtual del curso.

	Totalmente de acuerdo	De acuerdo	No tengo opinión	En desacuerdo	Totalmente en desacuerdo	No se aplica
El contenido fue útil para mi práctica	○	○	○	○	○	○
El material del curso mejoró mi capacidad para integrar el VIH	○	○	○	○	○	○
Los foros contribuyeron a mi aprendizaje	○	○	○	○	○	○
Fue dificil mantenerse al día con el trabajo del curso debido a responsabilidades familiares y laborales	○	○	○	○	○	○

Otros comentarios sobre el contenido del curso

4. Por favor evalúe esta parte virtual del curso

	Muy valiosas	Valiosas	Poco valiosas	Nada valiosas	No las usé
Las grabaciones de las conferencias	○	○	○	○	○
Las diapositivas y los archivos pdf para descargar	○	○	○	○	○
Las transcripciones y los archivos pdf para descargar	○	○	○	○	○
El material de lectura para descargar	○	○	○	○	○
El foro de discusión en línea	○	○	○	○	○
Las actividades evaluativas	○	○	○	○	○
Ayuda y aportación de los tutores	○	○	○	○	○

Otros comentarios sobre el valor del curso

5. Por favor evalúe su experiencia del sitio web del curso http://cursospaises.campusvirtualsp.org

	Totalmente de acuerdo	De acuerdo	No tengo opinión	En desacuerdo	Totalmente en desacuerdo	No se aplica
El proceso de registro fue fácil	○	○	○	○	○	○
El sitio web está bien organizado	○	○	○	○	○	○
El acceso a las conferencias fue fácil	○	○	○	○	○	○
Las evaluaciones cualitativas (ej. ensayo, reflexiones) del Tema 1 contribuyeron a mi aprendizaje	○	○	○	○	○	○
Contrastar lo que escribí en la reflexión inicial con lo que escribí en la reflexión final me permitió constatar mi aprendizaje	○	○	○	○	○	○
Las evaluaciones iniciales y finales del Tema 2 contribuyeron a mi aprendizaje	○	○	○	○	○	○
La estructura de calificación fue justa y razonable	○	○	○	○	○	○
La comunicación por correo electrónico con los tutores fue fácil y eficiente	○	○	○	○	○	○
Los correos semanales enviados por la coordinación académica con las instrucciones de aprendizaje fueron útiles y eficientes	○	○	○	○	○	○

Otros comentarios sobre el sitio web del curso

6. Por favor evalúe los foros semanales

	Totalmente de acuerdo	De acuerdo	No tengo opinión	En desacuerdo	Totalmente en desacuerdo	No se aplica
Fue fácil participar en los foros	○	○	○	○	○	○
Fueron dinámicos y accesibles a hacer preguntas	○	○	○	○	○	○
Recibimos respuestas en un tiempo apropiado	○	○	○	○	○	○
Las respuestas eran útiles	○	○	○	○	○	○
Se presentaron casos prácticos e instructivos	○	○	○	○	○	○
El foro fomentó discusiones interesantes entre los alumnos	○	○	○	○	○	○

Otros comentarios sobre los foros semanales

7. Por favor evalúe los correos electrónicos

	Totalmente de acuerdo	De acuerdo	No tengo opinión	En desacuerdo	Totalmente en desacuerdo	No se aplica
Fue fácil communicarse con los tutores y la coordinación académica por correo electrónico	○	○	○	○	○	○
Recibimos respuestas en un tiempo apropiado	○	○	○	○	○	○
Las respuestas eran útiles	○	○	○	○	○	○

Otros comentarios sobre los foros semanales

8. Por favor evalúe los CDs

	Totalmente de acuerdo	De acuerdo	No tengo opinión	En desacuerdo	Totalmente en desacuerdo	No se aplica
Fueron fáciles de usar	○	○	○	○	○	○
Los CDs han sido importantes para superar problemas de conectividad	○	○	○	○	○	○
Tenían todos los materiales que se necesitaba	○	○	○	○	○	○

Otros comentarios sobre los foros semanales

9. Por favor evalúe los tutores

	Totalmente de acuerdo	De acuerdo	No tengo opinión	En desacuerdo	Totalmente en desacuerdo	No se aplica
Los tutores facilitaron el proceso general del curso	○	○	○	○	○	○
Los tutores facilitaron el manejo técnico del curso	○	○	○	○	○	○
Los tutores facilitaron el proceso de aprendizaje	○	○	○	○	○	○
El número de alumnos por tutor era adecuado	○	○	○	○	○	○
Los tutores respondieron las preguntas y correos de los alumnos a tiempo y con información correcta	○	○	○	○	○	○
Los tutores clarificaron los objetivos del curso y responsabilidades de los alumnos	○	○	○	○	○	○
Los tutores corrigieron los trabajos imparcialmente y proporcionaron sugerencias instructivas	○	○	○	○	○	○
Mi tutor ha sido un elemento fundamental para poder terminar este curso	○	○	○	○	○	○

Otros comentarios sobre los tutores

10. Por favor díganos su opinión acerca de:

	Totalmente de acuerdo	De acuerdo	No tengo opinión	En desacuerdo	Totalmente en desacuerdo	No se aplica
El Ministerio de Salud me brindó apoyo continuo para participar en este curso	○	○	○	○	○	○
Las autoridades locales me brindaron apoyo continuo para participar en este curso	○	○	○	○	○	○
En mi centro de trabajo se me brindó apoyo continuo para poder hacer el curso	○	○	○	○	○	○
Tuve las condiciones tecnológicas y conexiones a internet necesarias para hacer el curso virtual	○	○	○	○	○	○

Otros comentarios sobre los tutores

11. Por favor marque su nivel de acuerdo con las siguientes frases

	Totalmente de acuerdo	De acuerdo	No tengo opinión	En desacuerdo	Totalmente en desacuerdo	No se aplica
Tengo capacidad para cuidar a pacientes con VIH.	◯	◯	◯	◯	◯	◯
Puedo aconsejar a pacientes sobre la prueba de VIH.	◯	◯	◯	◯	◯	◯
Puedo proporcionar resultados de VIH (positivos o negativos)a los pacientes.	◯	◯	◯	◯	◯	◯
Tengo conocimiento sobre la transmisión del VIH.	◯	◯	◯	◯	◯	◯
Puedo realizar la evaluación inicial del paciente con VIH.	◯	◯	◯	◯	◯	◯
Tengo conocimiento sobre la toxicidad antirretroviral.	◯	◯	◯	◯	◯	◯
Tengo capacidad de alentar a los pacientes a que adhieran a su tratamiento antirretroviral.	◯	◯	◯	◯	◯	◯
Sé qué intervenciones positivas de prevención son necesarias para pacientes con el VIH.	◯	◯	◯	◯	◯	◯
Tengo capacidad de detectar la TB en todos los pacientes con VIH.	◯	◯	◯	◯	◯	◯
Tengo conocimientos para proponer intervenciones que integren VIH con salud sexual, atención maternoinfantíl, y otros servicios en mi centro de atención.	◯	◯	◯	◯	◯	◯

12. Por favor evalúe cómo va a utilizar esta parte virtual del curso en su trabajo

	Totalmente de acuerdo	De acuerdo	No tengo opinión	En desacuerdo	Totalmente en desacuerdo	No se aplica
Voy a brindar consejo sin juzgar las conductas sexuales de los individuos que consultan	○	○	○	○	○	○
Voy a promover el acercamiento de mi centro a ONG y otras instituciones que trabajan en prevención y atención de VIH	○	○	○	○	○	○
Voy a crear mas canales de comunicación con los encargados de tratamiento a donde debo derivar los pacientes de mi centro	○	○	○	○	○	○
Voy a aprovechar mejor las oportunidades para hacer prevención sobre VIH	○	○	○	○	○	○
Puedo solicitar una prueba de VIH sin temores	○	○	○	○	○	○
Sé como voy a apoyar el seguimiento de un paciente en TARV.	○	○	○	○	○	○
Voy a ofrecer la prueba de VIH y sífilis a todas las mujeres embarazadas.	○	○	○	○	○	○
Voy a ofrecer la prueba de VIH a todos los pacientes con TB.	○	○	○	○	○	○

13. ¿Cuál sería lo más positivo en general de esta parte virtual del curso?

14. ¿Cuál sería lo más negativo en general de esta parte virtual del curso?

15. Otros comentarios que quisiera compartir con nosotros

3. Planificación de cursos en el futuro

16. Por favor ayude a mejorar y planificar los próximos cursos

	Totalmente de acuerdo	De acuerdo	No tengo opinión	En desacuerdo	Totalmente en desacuerdo
Yo le recomendaría este curso a un amigo o colega	◯	◯	◯	◯	◯
Tomaría un curso similar de nuevo	◯	◯	◯	◯	◯

17. Me gustaría tomar otro curso en línea sobre

	Alta prioridad	Algo interesado	No tengo opinión	No muy interesado	Baja prioridad	No se aplica
Prevención de la Transmisión Materno-Infantil del VIH	◯	◯	◯	◯	◯	◯
Cuidado de la Mujer Embarazada con VIH	◯	◯	◯	◯	◯	◯
Estudios de Laboratorio y Otros Diagnósticos en el Cuidado del Paciente con VIH	◯	◯	◯	◯	◯	◯
Co-infección con VIH y la Tuberculosis	◯	◯	◯	◯	◯	◯
Curso de Calidad de Atención	◯	◯	◯	◯	◯	◯
Curso de Atención Primaria de Salud	◯	◯	◯	◯	◯	◯
Curso de Fundamentos de Determinantes Sociales de Salud	◯	◯	◯	◯	◯	◯
Curso de Genero y Salud	◯	◯	◯	◯	◯	◯
Curso de Estadística Básica	◯	◯	◯	◯	◯	◯

Otro (por favor, especifíque)

18. Tenemos dificultades para apoyar estos cursos. Por favor, háganos saber de cualquier fuente de financiación que puedan estar interesadas en apoyar más cursos.

4. Final de la evaluación

Gracias por participar en esta evaluación.
