# Supplementary material for: A Novel Educational Strategy Targeting Health Care Workers in Underserved Communities in Central America to Integrate HIV into Primary Medical Care
Source: PLoS One. 2012 Oct 24;7(10):e46426. doi: 10.1371/journal.pone.0046426 (PMC3480350; doi:10.1371/journal.pone.0046426)
Supplement: Supporting Information S4 — Online Component Participant Feedback Survey. This survey was constructed and delivered using SurveyMonkey [13] and participants took the survey at the conclusion of the online component. (PDF) [file pone.0046426.s004.pdf]

HIV training program - Overall program participant feedback

1. Por favor, escriba su nombre aquí.

2. Por favor, escriba su correo electrónico aquí.

3. Mis habilidades en computación mejoraron al tomar este curso.

- ◯ Totalmente de acuerdo
- ◯ De acuerdo
- ◯ No tengo opinión
- ◯ En desacuerdo
- ◯ Totalmente en desacuerdo

4. ¿Qué nivel de habilidades técnicas tenía antes del curso? Por favor, califique las siguientes habilidades.

	Excelente	Muy bueno	Bueno	Regular	Deficiente
Abrir archivos	◯	◯	◯	◯	◯
Correo electrónico	◯	◯	◯	◯	◯
Abrir archivos adjuntos	◯	◯	◯	◯	◯
Acceder al internet	◯	◯	◯	◯	◯
Navegar por la Internet (búsqueda de información)	◯	◯	◯	◯	◯
Cargar/Descargar archivos	◯	◯	◯	◯	◯
Instrucción/curso virtual previo	◯	◯	◯	◯	◯

5. ¿Qué nivel de habilidades técnicas tenía después del curso? Por favor, califique las siguientes habilidades.

	Excelente	Muy bueno	Bueno	Regular	Deficiente
Abrir archivos	◯	◯	◯	◯	◯
Correo electrónico	◯	◯	◯	◯	◯
Abrir archivos adjuntos	◯	◯	◯	◯	◯
Acceder al internet	◯	◯	◯	◯	◯
Navegar por la Internet (búsqueda de información)	◯	◯	◯	◯	◯
Cargar/Descargar archivos	◯	◯	◯	◯	◯
Instrucción/curso virtual previo	◯	◯	◯	◯	◯

6. ¿Tiene acceso a una computadora en . . .?

	Sí	A veces	No
La casa	◯	◯	◯
El trabajo	◯	◯	◯
Un café internet	◯	◯	◯

7. ¿Tiene acceso al Internet en . . .?

	Sí	A veces	No
La casa	◯	◯	◯
El trabajo	◯	◯	◯
Un café internet	◯	◯	◯

8. ¿Donde se conectó al Internet para completar el trabajo del curso?

- ◯ Principalmente en la casa
- ◯ Principalmente en el trabajo
- ◯ Un café internet
- ◯ Otro (por favor especifique)

9. ¿Vive en una zona rural o urbana?

- ◯ Rural
- ◯ Urbana

10. ¿El lugar donde trabaja se encuentra en un área rural o urbana?

- ◯ Rural
- ◯ Urbana

11. Su práctica o consultorio clínico está principalmente ubicado en un/a

- ◯ Hospital - Centro de tercer nivel de atención (hospital de referencia)
- ◯ Hospital comunitario
- ◯ Clínica pequeña (menos de 3 médicos)
- ◯ Clínica mediana (3 a 8 médicos)
- ◯ Clínica grande (+8 médicos)

12. El curso virtual me preparó para el curso presencial.

- ◯ Totalmente de acuerdo
- ◯ De acuerdo
- ◯ No tengo opinión
- ◯ En desacuerdo
- ◯ Totalmente en desacuerdo

13. Tomando sólo el curso virtual hubiera sido suficiente para integrar el VIH en el primer nivel de atención.

- ◯ Totalmente de acuerdo
- ◯ De acuerdo
- ◯ No tengo opinión
- ◯ En desacuerdo
- ◯ Totalmente en desacuerdo

14. La parte presencial contribuyó significativamente a mi capacidad de aplicar los conocimientos adquiridos en el curso virtual en mi práctica/clínica.

- ◯ Totalmente de acuerdo
- ◯ De acuerdo
- ◯ No tengo opinión
- ◯ En desacuerdo
- ◯ Totalmente en desacuerdo

15. El proceso de preparación de las propuestas de proyectos ha contribuido significativamente a mi capacidad de aplicar los conocimientos y/o habilidades adquiridos en la parte virtual y presencial en mi práctica/clínica.

- ◯ Totalmente de acuerdo
- ◯ De acuerdo
- ◯ No tengo opinión
- ◯ En desacuerdo
- ◯ Totalmente en desacuerdo

16. Califique la importancia de cada parte del curso en su experiencia de aprendizaje en general.

	Muy importante	Importante	Moderamente importante	Poco importante	Muy poco importante	No se aplica
Virtual	◯	◯	◯	◯	◯	◯
Presencial	◯	◯	◯	◯	◯	◯
Propuesta de proyecto	◯	◯	◯	◯	◯	◯

17. Quiero llevar a cabo el proyecto con el fin de mejorar la atención del VIH en mi centro de trabajo.

◯ Sí

◯ No

Si no, ¿por qué? (por ejemplo, la falta de recursos, falta de apoyo del supervisor, falta de tiempo, falta de financiación)

18. ¿Podemos contactarlo en 6 meses para ver el progreso de su proyecto?

◯ Sí

◯ No

19. En mi experiencia de aprendizaje, los siguientes factores crearon

	Muchas dificultades	Pocas dificultades	Ninguna dificultad	No tengo opinión
Conectividad al Internet	◯	◯	◯	◯
Acceso a una computadora	◯	◯	◯	◯
Tiempo	◯	◯	◯	◯
Apoyo de mi familia	◯	◯	◯	◯
Apoyo en el trabajo	◯	◯	◯	◯
Apoyo del gobierno	◯	◯	◯	◯

20. ¿Qué componentes educativos del curso entero contribuyeron más a su aprendizaje? (Selecione 5)

- [ ] Tutores
- [ ] Conferencias grabadas
- [ ] Las transcripciones y archivos pdf para descargar
- [ ] Las diapositivas y archivos pdf para descargar
- [ ] Las pruebas pre y post
- [ ] Los CDs
- [ ] Casos clínicos
- [ ] Centro de simulación
- [ ] Personificación de situaciones clínicas
- [ ] Ensayos reflexivos
- [ ] Material de referencia
- [ ] Desarrollo de la propuesta de proyecto
- [ ] La presentación de la propuesta de proyecto

Muchas gracias por su participación y esperamos que esta experiencia haya sido productiva.

21. Si tiene algún otro comentario, por favor indíquelo debajo:
