# Supplementary material for: A Novel Educational Strategy Targeting Health Care Workers in Underserved Communities in Central America to Integrate HIV into Primary Medical Care
Source: PLoS One. 2012 Oct 24;7(10):e46426. doi: 10.1371/journal.pone.0046426 (PMC3480350; doi:10.1371/journal.pone.0046426)
Supplement: Supporting Information S5 — Overall Program Participant Feedback Survey. This survey was constructed and delivered using SurveyMonkey [13] and participants took the survey after completing all three components. (PDF) [file pone.0046426.s005.pdf]

Curso Integración del VIH y otras enfermedades infecciosas prevalentes en el 1er nivel de atención

Pan American Health Organization

JOHNS HOPKINS
UNIVERSITY

CENTRO DE INVESTIGACIÓN EPIDEMIOLOGICA EN SALUD SEXUAL Y REPRODUCTIVA

Manual de Tutores

Este Manual ha sido elaborado por:

Dra. Juana Elvira Suárez Conejero
Dra. Laura Magaña Valladares
Lic. Lorena Ruiz Relloso

Y con la especial colaboración del Dr. Omar Sued.
Organización Panamericana de la Salud

ÍNDICE

PROPÓSITO 4

PARTE I..... 5
LA EDUCACIÓN POR COMPETENCIAS, LA MULTIMODALIDAD Y EL TUTOR.

CAPÍTULO I. EL PROCESO DE ENSEÑANZA - APRENDIZAJE..... 5
1.1 LA EDUCACIÓN PARA ADULTOS. 5
1.3 LA EDUCACIÓN POR COMPETENCIAS..... 12
1.4 LAS ESTRATEGIAS PARA EL APRENDIZAJE. 17
1.5 LA EVALUACIÓN DE LOS APRENDIZAJES. 21

CAPITULO II. LAS MODALIDADES EDUCATIVAS 24
2.1 LA EDUCACIÓN PRESENCIAL..... 24
2.2 LA EDUCACIÓN VIRTUAL 26
2.3 LA MULTIMODALIDAD 29

CAPÍTULO III. EL TUTOR..... 30
3.1 EL TUTOR EN LA MULTIMODALIDAD 30
3.2 EL PERFIL DEL TUTOR. 32
3.3 LA MISIÓN DEL TUTOR. 33
3.4 LAS HABILIDADES Y COMPETENCIAS REQUERIDAS DE UN TUTOR 34

PARTE II.. 37
EL TUTOR EN EL CURSO INTEGRACIÓN DEL VIH Y OTRAS ENFERMEDADES INFECCIOSAS PREVALENTES EN EL PRIMER NIVEL DE ATENCIÓN.

CAPÍTULO IV. LAS ESTRATEGIAS DE OPERACIÓN DEL CURSO. 37
4.1 LA EDUCACIÓN EN SALUD. EL PERFIL DE LOS ALUMNOS. 37
4.2 CARACTERÍSTICAS GENERALES DEL CURSO. 38
4.3 COMPETENCIAS Y MODELO PEDAGÓGICO. 40
4.4 EVALUACIÓN. 40
4.5 CALENDARIO. 44
4.6. PROGRAMA. DESCRIPCIÓN DE LAS UNIDADES DIDÁCTICAS. 45
4.7 LA PLATAFORMA TECNOLÓGICA. 69

CAPÍTULO V. EL ROL DEL TUTOR EN EL CURSO. 95
5.1 EL ROL DEL TUTOR DURANTE LA FASE VIRTUAL. 95
5.2 EL CALENDARIO DEL TUTOR DURANTE LA FASE VIRTUAL. 97
5.3 EL ROL DEL TUTOR DURANTE LA FASE PRESENCIAL DEL CURSO. 101
5.4 EL ROL DEL TUTOR EN LOS PROYECTOS DE INTERVENCIÓN ACCIÓN. LOS TALLERES NACIONALES. 101
5.5 LA MEDICIÓN DEL DESEMPEÑO DE LOS TUTORES 107

CAPÍTULO VI. EL FUTURO: IMPLEMENTAR ACCIONES Y CREAR COMUNIDADES DEL CONOCIMIENTO. 108
6.1 LAS LICENCIAS CREATIVE COMMONS. EL ACCESO LIBRE A LA INFORMACIÓN 108
6.2 LOS TUTORES COMO PROMOTORES DE CAPACITACIÓN EN LA REGIÓN: CONSTRUYENDO UNA RED Y UNA CULTURA HACIA LA INTEGRACIÓN DEL VIH Y OTRAS ENFERMEDADES INFECCIOSAS PREVALENTES EN EL 1ER. NIVEL DE ATENCIÓN.............................. 110

ANEXOS... 113

BIBLIOGRAFÍA 146

Pan American Health Organization

Propósito

Bienvenidos al Manual de Tutores del Curso "Integración del VIH y otras enfermedades infecciosas prevalentes en el 1er. nivel de atención". Este curso es un esfuerzo conjunto entre la Universidad Johns Hopkins de los Estados Unidos, la Organización Panamericana de la Salud (OPS), el Instituto Conmemorativo Gorgas de Estudios de Salud de Panamá y cuenta con el financiamiento del Departamento de Salud y Servicios Humanos de los Estados Unidos. Esta iniciativa busca apoyar a recursos humanos en salud para que desarrollen capacidades de organización y atención en el primer nivel que posibiliten mejorar las prestaciones de los servicios integrados para VIH y otras enfermedades infecciosas prevalentes.

En general, los tutores de este curso serán los encargados de apoyar a los alumnos durante todo el proceso de enseñanza aprendizaje. Es por ello que este Manual tiene dos objetivos fundamentales. El primero, ser una guía para el trabajo de los tutores durante el desarrollo de este proyecto. El segundo, que futuros lectores puedan disponer de un tutorial claro que posibilite replicar este curso en diferentes escenarios.

Este Manual, en su primera parte, brindará a sus lectores una panorámica de la educación y evaluación por competencias, en tanto modelo pedagógico de este curso. También les dará elementos de pedagogía que posibilitarán aplicar mayor dinamismo a la enseñanza así como un conjunto de recomendaciones para llevar a cabo la labor del tutor con calidad y excelencia. En la segunda parte de este Manual se definen los procedimientos de trabajo del tutor durante las diferentes fases del curso y también las expectativas futuras que tenemos con este material, el cual se pone a disposición de todos.

PARTE I

LA EDUCACIÓN POR COMPETENCIAS, LA MULTIMODALIDAD Y EL TUTOR.

CAPÍTULO I. EL PROCESO DE ENSEÑANZA - APRENDIZAJE.

1.1 LA EDUCACIÓN PARA ADULTOS.

Una buena manera de comenzar este Manual es tratar de saber cómo aprenden los adultos. Los participantes de este curso deben ser profesionales de la salud, con experiencia laboral, elemento que nos facilitará el proceso de aprendizaje.

Para lograr las condiciones ideales de aprendizaje en un adulto es muy importante que se emplee un método didáctico enfocado en el participante. Este método debe tomar en cuenta que los adultos se desempeñan mejor cuando:

- Ejercen cierto control y asumen la responsabilidad de dirigir su propio aprendizaje.

LOS ADULTOS SE DESEMPEÑAN MEJOR CUANDO EJERCEN CIERTO CONTROL Y ASUMEN LA RESPONSABILIDAD DE DIRIGIR SU PROPIO APRENDIZAJE.

- Pueden utilizar su experiencia personal como punto de partida para el nuevo aprendizaje y como referencia a medida que el aprendizaje avanza.

- Se les proporcionan oportunidades para reflexionar sobre sus experiencias personales y para transformar dicha reflexión en aprendizaje.

- El aprendizaje es activo y pueden aprender por medio de actividades, teniendo la oportunidad de aplicar la teoría a sus prácticas.

- La tarea de aprendizaje está enfocada a algún problema específico, es decir, cuando tratan con problemas y buscan soluciones de aplicación inmediata.

- Pueden compartir ideas con otros y aprovechar así sus experiencias.

- Se encuentran en un ambiente tranquilo y propicio para el estudio, donde tengan confianza en los procedimientos, en la actitud del personal y de los miembros del grupo, así como en los objetivos del programa de aprendizaje.

- Se involucran a "negociar" el aprendizaje, participan en la responsabilidad de proyectar, conducir y evaluar el programa de aprendizaje, y expresan su preferencia por los métodos de estudio.

En general, los adultos se involucran en el aprendizaje una vez que están convencidos que los conocimientos a adquirir les permitirán lograr un cambio positivo. Es por ello que el principal rol de un tutor

LOS ADULTOS SE DESEMPEÑAN MEJOR CUANDO PUEDEN COMPARTIR IDEAS CON OTROS Y APROVECHAR ASÍ SUS EXPERIENCIAS.

en la educación de adultos es mostrarle a los participantes los beneficios que se obtendrán al aplicar los conocimientos adquiridos.

Una buena manera de aprender a enseñar, además de conocer las características de aprendizaje de los alumnos, es contar con herramientas pedagógicas que posibiliten sacar el mayor provecho en el este proceso. Por esta razón, resulta básico que el lector de este Manual cuente con los conocimientos más importantes acerca de la historia de la pedagogía y los conocimientos más actualizados con que se cuenta al respecto. A continuación brindamos algunos elementos indispensables al respecto.

1.2 LOS PARADIGMAS EDUCATIVOS.

Antes de la existencia de la imprenta y de la difusión masiva de los libros, cuando solamente unos pocos accedían a la cultura, el profesor o tutor de familia era prácticamente el único proveedor de información (junto con las bibliotecas). La enseñanza estaba centrada en el profesor y el proceso de aprendizaje solo buscaba la memorización del saber, el cual transmitía el maestro de manera sistemática, estructurada, didáctica.

Poco a poco, los libros se fueron difundiendo entre la sociedad y la cultura se fue extendiendo entre las diversas capas sociales. El libro de texto complementaba las explicaciones magistrales del profesor y a veces sugería ejercicios a realizar para reforzar los aprendizajes. El profesor era un instructor y la enseñanza se centraba en los contenidos que el alumno debía memorizar y aplicar para contestar preguntas y realizar ejercicios que le ayudaban a asimilar los contenidos.

UNA BUENA MANERA DE APRENDER A ENSEÑAR ES CONTAR CON HERRAMIENTAS PEDAGÓGICAS QUE POSIBILITEN SACAR EL MAYOR PROVECHO EN EL PROCESO DE ENSEÑANZA APRENDIZAJE.

A principios del siglo XX y con la progresiva "democratización del saber" surge la idea de la "escuela activa". Se considera entonces que el alumno no debe estar pasivo recibiendo y memorizando la información que le proporcionan el profesor y el libro de texto. En este enfoque, la enseñanza debe proporcionar entornos de aprendizaje ricos en recursos educativos (información bien estructurada, actividades adecuadas y significativas) en los que los estudiantes puedan desarrollar proyectos y actividades que les permitan descubrir el conocimiento, aplicarlo en situaciones prácticas y desarrollar todas sus capacidades (experimentación, descubrimiento, creatividad, iniciativa). La enseñanza se centra en la actividad del alumno, que a menudo debe ampliar y reestructurar sus conocimientos para poder hacer frente a las problemáticas que se le presentan. Se busca que el alumno participe, se implique, adquiera responsabilidades.

A finales del siglo XX gracias a la evolución del pensamiento pedagógico y a la Revolución de las Tecnologías de la Información y las Comunicaciones se configura una nueva sociedad: la de la Información y el Conocimiento. En este marco, con el acceso cada vez más abierto de los ciudadanos a los medios de comunicación y a Internet, junto al auge de las redes, se va abriendo paso un nuevo paradigma: "la enseñanza constructivista".

Derivado del análisis histórico anterior se destacan tres formas de concebir la educación o paradigmas educativos: el conductista, el cognitivista y el constructivista. A continuación sus aspectos más importantes.

DEL ANÁLISIS HISTÓRICO SE DESTACAN TRES PARADIGMAS EDUCATIVOS: CONDUCTISMO, COGNITIVISMO Y CONSTRUCTIVISMO.

El Paradigma Conductista

Sus inicios se remontan a las primeras décadas el siglo XX y su fundador fue J.B. Watson. Las bases del conductismo watsoniano se encuentran en las obras de autores como Pavlov y Thorndike. En este paradigma el aprendizaje es definido como un cambio observable en el comportamiento, por lo que el estudio del aprendizaje debe enfocarse en fenómenos observables y medibles. Sus fundamentos nos hablan de un aprendizaje producto de una relación "estímulo-repuesta", por lo que procesos tales como el pensamiento y la motivación no son considerados relevantes para el aprendizaje. Para los conductistas el aprendizaje ocurre únicamente cuando se observa un cambio en el comportamiento. Si no hay cambio observable no hay aprendizaje. Los principios de las ideas conductistas pueden aplicarse con éxito en la adquisición de conocimientos memorísticos, que suponen niveles primarios de comprensión, como por ejemplo el aprendizaje de las tablas de multiplicar.

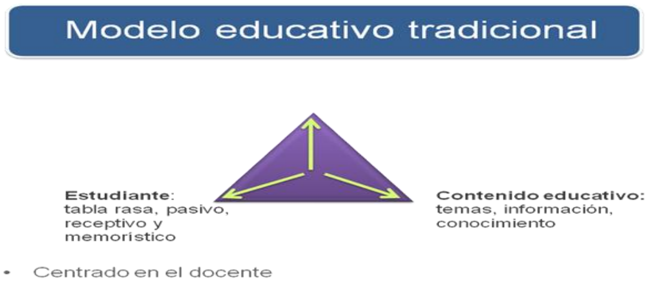

Este paradigma o forma de enseñanza tiene una limitación importante: la repetición no garantiza la asimilación de nuevas conductas sino sólo su ejecución (el alumno sabe multiplicar, pero no sabe cuándo debe hacerlo, ni sabe resolver un problema en el que tiene que utilizar la multiplicación). La situación aprendida no es fácilmente traspasable a otras situaciones. El conductismo, es uno de los paradigmas de mayor tradición y pese a las numerosas críticas aún tiene gran vigencia en nuestra cultura.

EN EL CONDUCTISMO SE CONCIBE EL APRENDIZAJE A TRAVÉS DE LA REPETICIÓN MEMORÍSTICA.

El Paradigma Cognitivo

Este paradigma surge a comienzos de los años sesenta del siglo pasado y es precisamente la teoría que sustituye a las perspectivas conductistas. Durante los años 1960 y 1980 las teorías de Jean Piaget desempeñaron un papel decisivo como punto de referencia teórico para la investigación pedagógica y las innovaciones en educación. Piaget puso de manifiesto que el crecimiento intelectual no consiste en una adición de conocimientos, sino en grandes periodos de reestructuración y, en muchas ocasiones, de re-restructuración de las mismas informaciones. Aprender es sinónimo de comprender, por lo tanto, solo lo que se logre comprender será lo que se aprenda y se recuerde, porque quedará integrado en las estructuras de conocimientos.

Otro eminente precursor del cognitivismo es Lev Vygotsky, quien concibe al sujeto como un ser eminentemente social y al conocimiento como un producto social. Parte de la idea de que el aprendizaje no es una actividad individual sino grupal, porque considera que todos los procesos psicológicos de orden superior (comunicación, lenguaje y razonamiento) se adquieren primero en un contexto social y luego se interiorizan. Esta interiorización es producto del uso de un determinado comportamiento cognitivo en un contexto social.

El Paradigma Constructivista

El constructivismo es una posición compartida por diferentes tendencias de la investigación psicología y educativa. Se basa en la premisa de que el aprendizaje es esencialmente activo, es un proceso subjetivo que cada persona va modificando constantemente a la luz de sus experiencias.

EL COGNITIVISMO PLANTEA QUE APRENDER ES SINÓNIMO DE COMPRENDER.

Tiene su génesis en el pensamiento de un sinnúmero de autores: Ausubel, Bruner, Dewey, Gagné, Piaget, Novak, Vygotsky, entre otros. El constructivismo es un paradigma postmoderno que parte del principio de que la adquisición del conocimiento es un proceso gradual, que tiene lugar en el propio sujeto y para el cual la interacción social es determinante. Ello implica nuevos roles para los docentes y los alumnos:

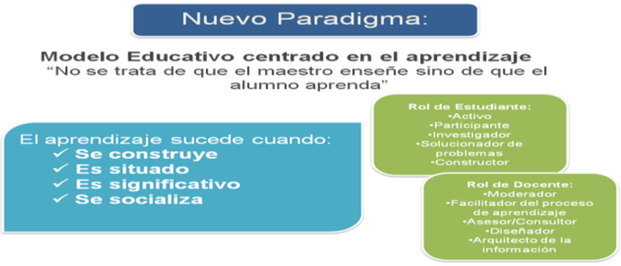

El aprendizaje es un proceso de construcción del conocimiento, mientras que la enseñanza es un proceso de intervención que facilita el aprendizaje. El alumno tiene un rol activo en la construcción de mecanismos de comprensión y comunicación y esta es la gran diferencia del constructivismo con el conductismo o paradigma tradicional. En resumen, el profesor se convierte en un mediador de los aprendizajes de los estudiantes. Éstos trabajan colaborativamente entre ellos y con el profesor. El objetivo ahora es construir conocimientos.

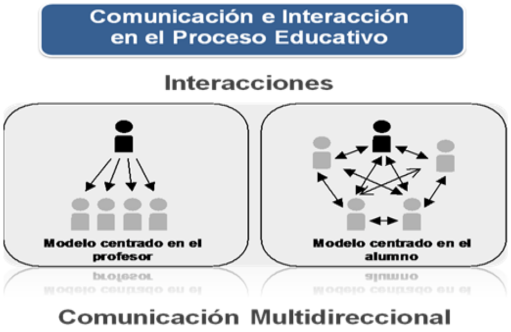

EN EL CONSTRUCTIVISMO EL APRENDIZAJE ES UN PROCESO DE CONSTRUCCIÓN DEL CONOCIMIENTO, MIENTRAS QUE LA ENSEÑANZA ES UN PROCESO DE INTERVENCIÓN QUE FACILITA EL APRENDIZAJE.

1.3 LA EDUCACIÓN POR COMPETENCIAS.

A partir del desarrollo y auge del constructivismo surge la educación por competencias. Inicialmente tuvo sus orígenes en el mundo empresarial, dado el desbalance existente entre los profesionales formados por los sistemas de enseñanza y las necesidades de especialistas competentes que exigía el mundo laboral. Más allá de estos inicios y de cualquier trasfondo ideológico que pueda haber estado insertado en él, la educación basada en competencias es una tendencia cada vez más importante en el ámbito educativo a nivel mundial, como reacción ante una educación tradicional, memorística, y poco vinculada con el mundo laboral que se expresa en los paradigmas conductista y, en menor medida, en el cognitivista.

LA EDUCACIÓN POR COMPETENCIAS NACE CON EL AUGE DEL CONSTRUCTIVISMO.

Competencia significa básicamente "saber de ejecución" y puesto que "todo conocer" se traduce en un saber, entonces es posible decir que competencia y saber son recíprocos. Así podemos hablar de saber pensar, saber desempeñar, saber interpretar, saber actuar en diferentes escenarios, entre otros.

La UNESCO define las competencias como el conjunto de comportamientos socio-afectivos y habilidades cognoscitivas, psicológicas, sensoriales y motoras que permiten llevar a cabo adecuadamente un desempeño, una función, una actividad o una tarea.

La OCDE asume que las competencias son capacidades para responder a las demandas y llevar a cabo tareas de forma adecuada. Cada competencia se construye a través de la combinación de habilidades cognitivas y prácticas, conocimientos, motivación, valores, actitudes, emociones y otros componentes sociales y conductuales.

Autores como Andrew Gonzci y James Athanasou consideran que la competencia es una compleja combinación de atributos (conocimientos, habilidades, actitudes y valores) que se ponen en juego para que los estudiantes interpreten la situación específica en que se encuentran y actúen en consecuencia. Este enfoque es holístico, en el sentido que integra y relaciona atributos y tareas (...), nos permite incorporar la ética y los valores como elementos del desempeño competente, la importancia del contexto y el hecho de que es posible ser competente de diversas maneras.[1]

Para Phillipe Perrenound una competencia es la facultad de movilizar un conjunto de recursos cognoscitivos (conocimientos, capacidades, información, etcétera) para enfrentar con pertinencia y eficacia una familia de situaciones.

El modelo pedagógico por competencias está comprometido con la calidad e innovación educativa a través de programas académicos pertinentes, mediante los cuales se busca que los alumnos se apropien significativamente de las demandas de su profesión: lenguajes, responsabilidades, funciones, métodos, posibilidades de acción, etcétera. En síntesis, se busca instalar un perfil de profesional capaz de realizar una práctica competente a partir de la asimilación y aplicación de conocimientos, habilidades, actitudes y valores necesarios para el ejercicio cotidiano de su labor.

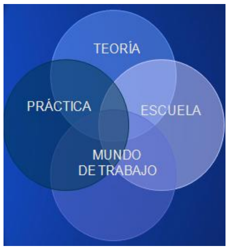

UNA COMPETENCIA PUEDE SER DEFINIDA COMO LA FACULTAD DE MOVILIZAR UN CONJUNTO DE RECURSOS COGNOSCITIVOS (CONOCIMIENTOS, CAPACIDADES, INFORMACIÓN, ETCÉTERA) PARA ENFRENTAR CON PERTINENCIA Y EFICACIA UNA FAMILIA DE SITUACIONES.

---

[1] Gonzci Andrew y James Athanasou, “Instrumentación de la educación basada en competencias: perspectivas de la teoría y la práctica en Australia”, en Antonio Argüelles (comp.) Competencia Laboral y Educación Basada en Normas de Competencia, México: Limusa, p. 275.

En el modelo pedagógico que proponemos para este curso se define a la competencia como la interacción dinámica de un conjunto de conocimientos (saber), habilidades (saber hacer) y actitudes (saber ser) que permiten a una persona desempeñar ciertas actividades de manera eficiente y efectiva. Desde esta perspectiva, conceptualizar a la educación o capacitación basada en competencias implica también el reconocimiento de sus componentes: filosófico, conceptual y psicopedagógico, los cuales describiremos a continuación:

Componente filosófico

La educación basada en el aprendizaje por competencias pretende la formación de sujetos integralmente desarrollados, es decir, profesionales que muestren desempeños competentes y pertinentes con su entorno social y productivo, de manera que posibiliten el desarrollo de las organizaciones y de la sociedad en general. Este modelo educativo se sustenta en los cuatro pilares de la educación para el milenio que propone la UNESCO: aprender a conocer, aprender a hacer, aprender a convivir y aprender a ser. Por ello, la filosofía intrínseca del proceso nuestro proceso educativo busca generar competencias en los estudiantes que promuevan valores humanos tales como responsabilidad, honestidad, compromiso, creatividad, innovación, cooperación, solidaridad, trabajo en equipo, pluralismo, humanismo, entre otros.

Componente conceptual

Este modelo educativo, por otra parte, enfatiza la construcción y/o desarrollo de conocimientos, habilidades y actitudes que permitan a los estudiantes insertarse adecuadamente en su estructura laboral y adaptarse a los cambios.

LA EDUCACIÓN BASADA EN EL APRENDIZAJE POR COMPETENCIAS PRETENDE LA FORMACIÓN DE SUJETOS INTEGRALMENTE DESARROLLADOS.

SUS PILARES SON: APRENDER A CONOCER, APRENDER A HACER, APRENDER A CONVIVIR Y APRENDER A SER.

Por ello, las competencias se definen (como nos referimos con anterioridad) como la generación de capacidades efectivas en los alumnos para llevar a cabo exitosamente su actividad laboral. Las competencias no son una probabilidad de éxito en la ejecución de un trabajo sino una capacidad real y que debe ser demostrada. Implican un saber conocer, saber hacer, saber convivir y saber ser que deben poder ser transferidos con creatividad a cualquier contexto laboral o productivo.

Componente psicopedagógico

Este componente se basa en la necesidad de una práctica educativa centrada en el aprendizaje, la cual trascienda a la docencia tradicional. Los roles del estudiante y del docente cobran nuevos sentidos. La técnica educativa constructivista conlleva a que el estudiante se vea forzado a construir el aprendizaje a través de la interacción con la información y los contenidos, asumiendo una actitud crítica, creativa y reflexiva que le permita ir aplicando lo que aprende en su entorno. Desde esta perspectiva, el estudiante se vuelve gestor de su propio aprendizaje. Por su parte, el docente y el propio diseño de contenidos serán los responsables de propiciar ambientes de aprendizaje que promuevan actitudes abiertas posibiliten el desarrollo de habilidades para que los estudiantes:

- Aprendan a aprender (a regular sus procesos de aprendizaje, es decir, a darse cuenta de qué y cómo aprenden).

- Aprendan a hacer (a desarrollar habilidades que les permita aplicar lo aprendido en beneficio de su entorno).

LAS COMPETENCIAS NO SON UNA PROBABILIDAD DE ÉXITO EN LA EJECUCIÓN DE UN TRABAJO SINO UNA CAPACIDAD REAL Y QUE DEBE SER DEMOSTRADA.

- Aprendan a convivir (a trabajar en equipo con pluralismo, incorporando una cultura de la legalidad y la ética a su formación y desempeño profesional).

- Aprendan a ser (a visualizarse como seres orientados hacia la universalidad y el humanismo, comprometidos con su formación profesional y con el desarrollo de la sociedad).

Un conjunto de premisas garantizan la correcta y exitosa operación de este modelo:

Negociar y conducir con los alumnos los proyectos de trabajo: La relación profesor (tutor) –estudiante se fundamenta en la participación activa del alumno y en el papel del docente como guía del mismo. El profesor (tutor) orienta, corrige, da sugerencias, brinda información externa que le permita al estudiante mejorar sus competencias como profesionista, es un animador del debate que favorece la pregunta, la investigación, la consulta.

Utilizar los conocimientos o saberes como recursos para movilizar: Trabajar por competencias no supone renunciar a la trasmisión de conocimientos, sino hacer buen uso de ellos. Los conocimientos son recursos determinantes para que los alumnos puedan resolver de manera adecuada un problema y tomar decisiones.

Trabajar con situaciones-problema: Para movilizar conocimientos, habilidades, actitudes y valores es necesario que el profesor (tutor) diseñe y utilice actividades que impliquen un verdadero reto cognitivo para los alumnos. Sólo se adquieren competencias cuando ponemos los conocimientos adquiridos ante situaciones-problema complejas, retadoras, realistas, contextualizadas y significativas.

EL MODELO DE EDUCACIÓN POR COMPETENCIAS SE BASA EN UNA SERIE DE PREMISAS BÁSICAS QUE COMPETEN A LOS ROLES DEL DOCENTE Y DEL ALUMNO.

Diversificar las formas de enseñar, implementar nuevas estrategias didácticas: Si se parte de la idea que cada alumno aprende de manera diferente y tiene intereses y necesidades distintas, es necesario que el profesor (tutor) instale una pedagogía diferenciada que le brinde a cada quien justo lo que necesita. Un profesor (tutor) puede hacer uso de diferentes formas de trabajo en el aula.

Practicar nuevas maneras de evaluar los aprendizajes de los alumnos: El enfoque de evaluación por competencias se concentra en el dominio de lo aprendido y en el resultado del aprendizaje, propiciando experiencias donde el desempeño es el criterio fundamental para evaluar la apropiación de conocimientos, habilidades y actitudes.

Asumir una mayor cercanía los campos del conocimiento: El profesor (tutor) que trabaja por competencias debe asumir como compromiso la formación integral del estudiante y no sólo de su módulo o tema. Esto supone trabajar colaborativamente con otros profesores (tutores) a fin de identificar puntos de conexión y construir puentes transversales para lograr una formación más consistente y articulada.

1.4 LAS ESTRATEGIAS PARA EL APRENDIZAJE.

Uno de los grandes desafíos de la educación por competencias y específicamente del docente es cómo hacer para que el alumno aprenda a ser competente. Es común escuchar como respuesta a esta pregunta que lo que hay que hacer es diseñar e implementar "estrategias didácticas". Sin embargo, para utilizarlas es necesario saber qué son, aprender a planearlas con anticipación y definir cuál es el momento adecuado para realizarlas.

UNO DE LOS GRANDES DESAFÍOS DE LA EDUCACIÓN POR COMPETENCIAS, Y ESPECÍFICAMENTE DEL DOCENTE, ES CÓMO HACER PARA QUE EL ALUMNO APRENDA A SER COMPETENTE.

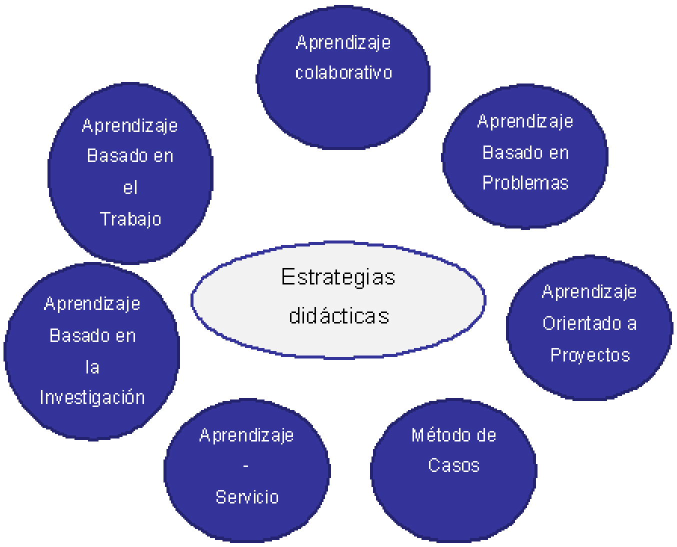

Las estrategias didácticas son un sistema de acciones o conjunto de actividades entre el profesor (tutor) y sus estudiantes, organizadas y planificadas por el docente, con la finalidad de posibilitar el aprendizaje de los estudiantes.

Entre otras, las estrategias didácticas pueden clasificarse como:

Estrategias de apoyo: Se ubican en el plano afectivo - motivacional y permiten al estudiante mantener un estado propicio para el aprendizaje.

Estrategias de aprendizaje o inducidas: Son aquellas que estimulan los procedimientos y habilidades que el alumno posee.

Estrategias de enseñanza: Son aquellas que facilitan el aprendizaje y comprensión de los alumnos.

Estrategias de aproximación a la realidad: son las que evitan los excesos teóricos mediante el contacto directo con las condiciones, problemas y actividades de la vida cotidiana.

LAS ESTRATEGIAS DIDÁCTICAS SON UN SISTEMA DE ACCIONES O CONJUNTO DE ACTIVIDADES DEL PROFESOR Y SUS ESTUDIANTES, ORGANIZADAS Y PLANIFICADAS POR EL DOCENTE, CON LA FINALIDAD DE POSIBILITAR EL APRENDIZAJE DE LOS ESTUDIANTES.

Estrategias de búsqueda, organización y selección de la información: son las que preparan a los alumnos para localizar, sistematizar y organizar la información y el conocimiento a su alcance.

Estrategias de descubrimiento: Son aquellas estrategias que incitan el deseo de aprender, detonan los procesos de pensamiento y crean el puente hacia el aprendizaje independiente.

Estrategias de trabajo colaborativo: Son las que cohesionan al grupo, incrementan la solidaridad, la tolerancia, el respeto, la capacidad argumentativa. Permiten la apertura a nuevas ideas, procedimientos y formas de entender la realidad, multiplican las alternativas y rutas para abordar, estudiar y resolver los problemas.

Por supuesto que existen muchas más estrategias, solo hemos querido referirnos a las más utilizadas.

Para un uso adecuado de las estrategias es indispensable conocer los niveles de abstracción cognitiva. Estos son ascendentes y se muestran en el gráfico a continuación:

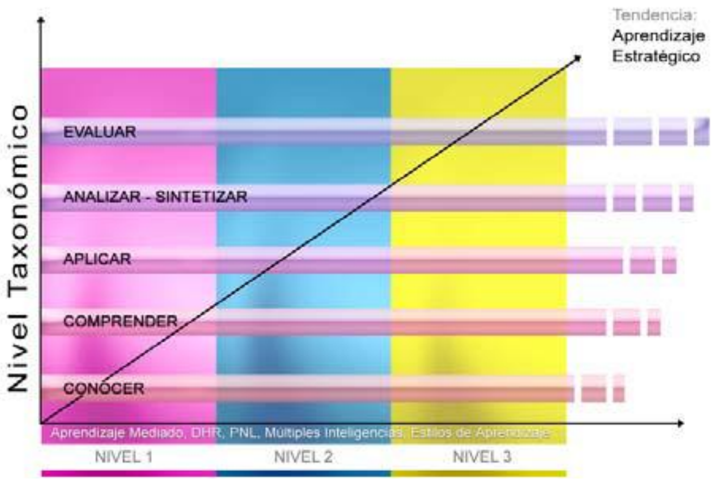

PARA UN USO ADECUADO DE LAS ESTRATEGIAS DIDÁCTICAS ES NECESARIO CONOCER LOS NIVELES DE ABSTRACCIÓN COGNITIVA.

Benjamin Bloom fue uno de los precursores de la taxonomía cognitiva. Esta se basa en la idea de que las operaciones cognitivas pueden clasificarse en seis niveles de complejidad creciente. Lo que tiene de taxonómico esta teoría es que cada nivel depende de la capacidad del alumno para desempeñarse en éste o en los niveles precedentes. Por ejemplo, la capacidad de evaluar – el nivel más alto de la taxonomía cognitiva – se basa en el supuesto de que el estudiante, para ser capaz de evaluar, tiene que disponer de la información necesaria, comprender esa información, ser capaz de aplicarla, de analizarla, de sintetizarla y, finalmente, de evaluarla. La taxonomía no es un mero esquema de clasificación, sino un intento de ordenar jerárquicamente los procesos cognitivos.

Clasificación de la Taxonomia de Bloom.

Para Bloom el campo cognoscitivo comprende el área intelectual que abarca las sub áreas: conocimiento, comprensión, aplicación, análisis, síntesis y evaluación. Por ello, se divide en seis procesos jerárquicos:

Conocimiento: Implica noción de hechos específicos, formas y medios de tratar con los mismos. Son, de modo general, elementos que deben memorizarse.

Comprensión: Concierne el aspecto más simple del entendimiento que consiste en captar el sentido directo de una comunicación o de un fenómeno, como la comprensión de una orden escrita u oral, o la percepción de lo que ocurrió en cualquier hecho particular.

Aplicación: Es el área que concierne a la interrelación de principios y generalizaciones con casos particulares o prácticos.

LA TAXONOMÍA DE BLOOM ABARCA LAS SUB ÁREAS:
CONOCIMIENTO
COMPRENSIÓN
APLICACIÓN
ANÁLISIS
SÍNTESIS
Y EVALUACIÓN.

Pan American Health Organization

Análisis: Implica la división de un todo en sus partes y la percepción del significado de las mismas en relación con el conjunto.

Síntesis: Concierne a la comprobación de la unión de los elementos que forman un todo. Puede consistir en la producción de una comunicación, un plan de operaciones o la derivación de una serie de relaciones abstractas.

Evaluación: Comprende una actitud crítica ante los hechos. Puede estar en relación con juicios relativos a la evidencia interna y con juicios relativos a la evidencia externa.

1.5 LA EVALUACIÓN DE LOS APRENDIZAJES.

Un elemento esencial en la educación por competencias es la evaluación. Esta se concibe, en general, no como un acto final de selección de los participantes, sino como un elemento básico para el proceso de enseñanza - aprendizaje.

Es por ello que la evaluación en el enfoque por competencias es un proceso continuo, que apoya la construcción del aprendizaje y orienta a los participantes corrigiendo, estimulando, discriminando, señalando sus logros, realizando un seguimiento del proceso y analizando los productos o resultados.

En este sentido, la evaluación es un proceso integral, orientado hacia la adquisición, perfeccionamiento o mejora de las competencias de los estudiantes, por lo que no solo incluye al aprendizaje, sino al propio programa educativo y la actividad docente (tutorial).

UN ELEMENTO ESENCIAL EN LA EDUCACIÓN POR COMPETENCIAS ES LA EVALUACIÓN, EN TANTO HERRAMIENTA PARA EL APRENDIZAJE.

La evaluación puede conceptualizarse como un proceso dinámico, continuo y sistemático, enfocado hacia los cambios de las conductas y rendimientos, mediante el cual verificamos los logros adquiridos en función de los objetivos propuestos. Adquiere sentido en la medida que comprueba la eficacia y posibilita el perfeccionamiento de la acción docente.

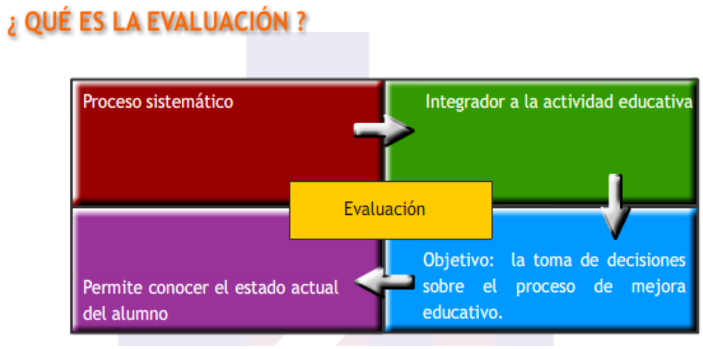

La gran mayoría de los autores (R. Tyler, B. Bloom, G. De Landsheere, B. Maccario) agrupan los objetivos y funciones de la evaluación en tres grandes categorías:

La Evaluación Predictiva o Inicial (Diagnóstica): se realiza para predecir un rendimiento o para determinar el nivel de aptitud previo al proceso educativo. Busca determinar cuáles son las características del alumno previas al desarrollo del programa, con el objetivo de ubicarlo en su nivel, clasificarlo y adecuar individualmente el nivel de partida del proceso educativo.

La Evaluación Formativa: es aquella que se realiza al finalizar cada tarea de aprendizaje y tiene por objetivo informar de los logros obtenidos y, eventualmente, advertir donde y en qué nivel existen dificultades de aprendizaje, permitiendo la búsqueda de nuevas estrategias educativas más exitosas. Aporta una retroalimentación permanente al desarrollo del programa educativo.

LA EVALUACIÓN PUEDE SER DIAGNÓSTICA, FORMATIVA O SUMATIVA.

La Evaluación Sumativa: tiene la estructura de un balance y es realizada después de un período de aprendizaje, al finalizar un programa o curso. Sus objetivos son calificar en función de un rendimiento, otorgar una certificación y determinar e informar sobre el nivel alcanzado a todos los niveles (alumnos, institución, docentes, tutores, etc.).

Por su parte, la evaluación tiene también sentido a partir de los contenidos que se van a evaluar. Estos son:

El contenido conceptual
Lo que busca es medir el nivel en que el alumno es capaz de profundizar el concepto y cada uno de los elementos que lo constituyen.

El contenido factual
Ya que revisa el conocimiento que hace referencia a lo relacionado con: terminologías y hechos específicos. Su intención es la la memorización del contenido por parte del alumno.

El contenido procedimental
Lo que busca es evaluar el conjunto de acciones ordenadas, dirigidas a la consecución de una meta y para ello es necesario dominar: contextualización, automatización, generalización y aplicación del procedimiento a situaciones específicas.

El contenido actitudinal
Las actitudes son sentimientos que muestran las preferencias hacia determinadas cosas y para ser desarrolladas evaluadas necesitan del elemento afectivo, conductual y cognitivo.

PARA EVALUAR CORRECTAMENTE ES NECESARIO CONOCER EL TIPO DE CONTENIDOS QUE SE VAN A EVALUAR.

SEGÚN EL TIPO DE CONTENIDO EXISTEN DIFERENTES MÉTODOS E INSTRUMENTOS DE EVALUACIÓN.

CAPITULO II. LAS MODALIDADES EDUCATIVAS

En la actualidad, el proceso de enseñanza aprendizaje se basa en dos modalidades principales: la presencial, forma clásica de este proceso, y la virtual, modalidad derivada del auge de las tecnologías de la información y las comunicaciones. Existe también la conocida como modalidad semipresencial o multimodal, que combina las dos anteriores. A continuación se describen:

LAS MODALIDADES EDUCATIVAS SON:

PRESENCIAL
VIRTUAL
MULTIMODAL

2.1 LA EDUCACIÓN PRESENCIAL

En la modalidad educativa presencial existe, como su nombre lo indica, una interacción física a través de un contacto directo visual y/o auditivo. Este tipo de educación tiene sus antecedentes o esencia histórica en los antiguos colegios griegos, donde se reunían las personas en torno a los grandes pensadores para oír sus charlas.

Para planificar una sesión un docente presencial debe:

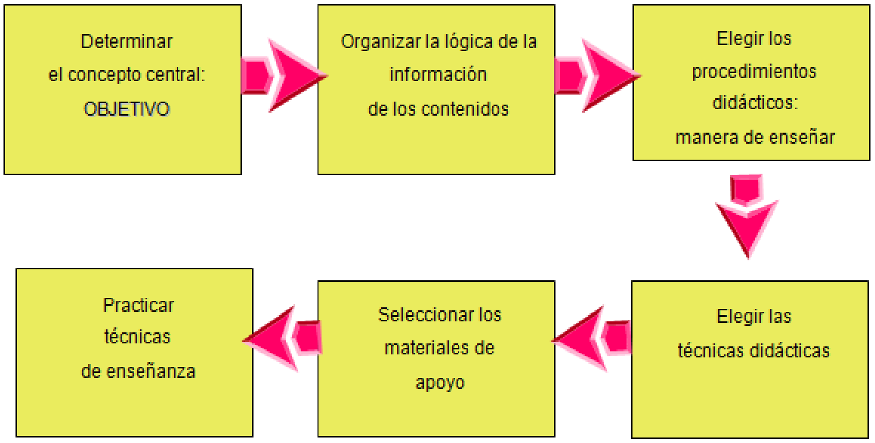

Aspectos básicos a considerar en la educación presencial

Antes de empezar la sesión el docente (tutor) deberá:	Ensayar la exposición.<br>Conocer los materiales a utilizar.<br>Asegurarse que el material esté completo, en orden y sea el adecuado.<br>Probar con anticipación el equipo cuando sea el caso.

Al principio de la sesión el docente (tutor) deberá:	Ser puntual.<br>Presentarse antes de comenzar la primera sesión.<br>Exponer las competencias esperadas, los objetivos y la forma en que se evaluarán.<br>Conocer a la audiencia y sus expectativas.<br>Generar confianza y credibilidad.

En el desarrollo de la sesión el docente (tutor) deberá:	Involucrar al grupo.<br>Conocer y dominar del tema.<br>Mantener contacto visual con la audiencia.<br>Desarrollar el tema de manera interesante, estructurando el objetivo, el desarrollo y las conclusiones.<br>Promover la participación de los asistentes.<br>Emplear material de apoyo.

Al finalizar la sesión el docente (tutor) deberá:	Hacer una recapitulación.<br>Aclarar dudas.<br>Fomentar el interés en temas posteriores si fuese el caso.

UN BUEN DOCENTE SIEMPRE CONOCE Y DOMINA SU TEMA, GENERANDO ASÍ CONFIANZA Y CREDIBILIDAD.

2.2 LA EDUCACIÓN VIRTUAL

La educación virtual surge, entre otras, gracias al desarrollo acelerado de la informática y las telecomunicaciones, desarrollo catalogado de Revolución Tecnológica dado el ritmo vertiginoso de su desarrollo y masificación.

El uso de las Tecnologías de la Información y las Comunicaciones (TIC) y específicamente de Internet aún se contabiliza en años. A solo 20 escasos años de su inicio para todo tipo de público, el 25% de la población mundial accede a Internet. Poco a poco Internet está dejando de ser una herramienta de lujo y se está convirtiendo en una herramienta de masas, aunque aún estemos lejos de las estadísticas ideales y persista fuertemente la brecha digital entre países y dentro de los mismos. El acceso a Internet a través de teléfonos o dispositivos móviles es una opción cada vez más socializada que hasta hace unos años era el privilegio de unos pocos.

Esta Revolución ha tenido un impacto directo en todas las esferas de la vida social, entre ellas en la educación. Con la masificación de Internet surge también la educación virtual, justo en el momento en que el paradigma pedagógico constructivista necesitaba nuevos vehículos para su expansión. Es importante recalcar esta idea porque de todos es conocido que la educación a distancia existía desde mucho antes y no tenía necesariamente ninguna vinculación con el desarrollo de las TIC. La enseñanza tradicional había encontrado modos de posibilitar los procesos asincrónicos: enseñanza por televisión, por correspondencia, etc. Pero la educación a distancia "clásica" no implicaba ni un cambio de paradigma educativo ni un soporte tecnológico revolucionario. Estos dos factores se conjugaron en la educación virtual.

CON LA MASIFICACIÓN DE INTERNET SURGE LA EDUCACIÓN VIRTUAL, JUSTO EN EL MOMENTO EN QUE EL PARADIGMA PEDAGÓGICO CONSTRUCTIVISTA NECESITABA NUEVOS VEHÍCULOS PARA SU EXPANSIÓN.

Hay dos elementos fundamentales que distinguen a la educación virtual. El primero, es la comunicación, la cual puede ser de dos tipos: sincrónica (en tiempo real) y asincrónica (diferida en el tiempo). El segundo elemento es el uso de materiales de aprendizaje diseñados y preparados específicamente para su utilización asincrónica y su reutilización, combinando aspectos didácticos y técnicos de estudio individual. El diseño de los materiales debe estar basado en lo que se sabe acerca de cómo aprende el individuo, cuánta información puede asimilar en cada ocasión, qué tipo de actividad práctica le ayuda a consolidar lo aprendido y otros hallazgos pedagógicos.

Si tenemos que resumir en una las ventajas de la educación virtual podemos afirmar que ella nos posibilita regresar un sinnúmero de veces al mismo objeto de aprendizaje, lo cual posibilita aumentar la eficacia del proceso de enseñanza. Otra ventaja básica son las posibilidades de implementar estrategias de enseñanza a amplios públicos, así como la reutilización de los cursos virtuales en tantas ediciones como se requiera.

Otras ventajas son:

- Eliminación de las barreras espacio-temporales entre el profesor y el estudiante.
- Extensión de la formación a un número mayor de personas.
- Flexibilización de la enseñanza.
- Favorecimiento del aprendizaje cooperativo y el autoaprendizaje.
- Conexión entre estudiantes dispersos geográficamente.
- Independencia geográfica y temporal de la acción formativa.
- Actualización y adaptación de contenidos de forma rápida y a bajo costo.

HAY DOS ELEMENTOS FUNDAMENTALES QUE DISTINGUEN A LA EDUCACIÓN VIRTUAL.

EL PRIMERO, ES LA COMUNICACIÓN, LA CUAL PUEDE SER DE DOS TIPOS: SINCRÓNICA (EN TIEMPO REAL) Y ASINCRÓNICA (DIFERIDA EN EL TIEMPO).

EL SEGUNDO ELEMENTO ES EL USO DE MATERIALES DE APRENDIZAJE DISEÑADOS Y PREPARADOS ESPECÍFICAMENTE PARA SU UTILIZACIÓN ASINCRÓNICA Y SU REUTILIZACIÓN.

- Reducción de costos al eliminar el desplazamiento de los profesores y de los estudiantes participantes en la acción formativa.
- Ritmos de aprendizaje marcados por los estudiantes.
- Ampliación de los escenarios para el aprendizaje: centros educativos, centros de trabajo, cibercafés, hogares, etc.

CON LA EDUCACIÓN VIRTUAL SE AMPLIAN LOS ESCENARIOS PARA EL APRENDIZAJE.

Aspectos básicos a considerar en la educación virtual

Durante el curso, el docente (tutor) deberá:	Presentar el curso y las normas de funcionamiento a los estudiantes.<br>Resolver dudas de forma individual y colectiva. Aclarar y profundizar conceptos y la información recibida.<br>Facilitar información adicional.<br>Señalar en los alumnos posibles mejoras para lograr una mejor comunicación, entendimiento y seguimiento del proceso.<br>Facilitar y negociar compromisos cuando existan diferencias entre los miembros del equipo.<br>Animar la participación de los estudiantes.<br>Fomentar el trabajo colaborativo por medio de distintas actividades.<br>Crear actividades individuales y grupales.<br>Asesorar en métodos de estudio en red.<br>Medir el desempeño del alumno en las actividades realizadas.<br>Desarrollar una evaluación continua formativa.

2.3 LA MULTIMODALIDAD

Finalmente, está la modalidad semipresencial o multimodal que, como decíamos anteriormente, combina la modalidad presencial con la virtual.

La multimodalidad en la actualidad se vuelve la forma de enseñanza por excelencia. Se aprovechan las posibilidades de la educación virtual y sus ventajas y se refuerza el proceso de comunicación a través de los espacios presenciales.

El componente virtual de la multimodalidad implica que, en gran medida, el rendimiento de los estudiantes dependa de un eficiente estudio individual de su parte, por lo que la orientación del profesor debe estar correctamente dirigida hacia los objetivos del programa de estudio y apoyada por materiales que permitan a los estudiantes orientarse en su auto-preparación.

La educación multimodal sigue diferentes esquemas:

1. Primero se realiza un período presencial y luego otro virtual.
2. Primero se realiza un período virtual y luego otro presencial.
3. Se mezclan indistintamente más de dos períodos.

EN LA ACTUALIDAD, LA MULTIMODALIDAD ES LA FORMA EDUCATIVA POR EXCELENCIA, PUES COMBINA LA MODALIDAD PRESENCIAL CON LA VIRTUAL.

CAPÍTULO III. EL TUTOR.

3.1 EL TUTOR EN LA MULTIMODALIDAD

La palabra tutor hace referencia a una figura que ejerce protección o tutela de otra persona. En educación multimodal, el tutor tiene como función fundamental fomentar el desarrollo del estudio independiente. Eso lo convierte en un orientador del aprendizaje del alumno, es cual carece, en ocasiones, de la presencia del docente (tutor) presencial habitual.

UN TUTOR ES UN FACILITADOR DEL PROCESO DE ENSEÑANZA APRENDIZAJE.

Ello implica que el tutor, además de facilitar el proceso de enseñanza aprendizaje a través de los elementos de pedagogía y didáctica que posee, debe ser un excelente comunicador y animador, sobre todo cuando se desempeña en los medios virtuales.

La principal diferencia entre la educación presencial y la virtual, o al menos la más notable para los alumnos, es la ausencia de una comunicación presencial y del lenguaje gestual al que cotidianamente estamos acostumbrados. Es por ello que el tutor en la multimodalidad, y especialmente en la etapa virtual de los cursos semipresenciales o multimodales, debe convertirse en un comunicador de excelencia, que maneje los procesos de comunicación sincrónica y asincrónica a la perfección.

Las herramientas más comunes de la comunicación sincrónica en entornos virtuales son los chats y las plataformas de comunicación como Webex y Elluminate. Estas herramientas posibilitan que los tutores sustituyan la comunicación habitual por una comunicación virtual en tiempo real.

En este caso, generalmente solo falta el lenguaje gestual para que la comunicación sea lo más parecida al cotidiano. Sin embargo, la mayor parte de las plataformas de comunicación sincrónica incorporan íconos gestuales que le permiten a los participantes expresar emociones más allá de las palabras o textos. Entre estos símbolos se destacan ☺ ☹ √ X, entre otros.

Las herramientas más comunes de comunicación asincrónica son los foros y el correo electrónico. El correo electrónico es la herramienta más conocida por lo que no nos detendremos en ella.

Los foros son una herramienta asíncrona de comunicación de excelencia, que permite a un grupo comunicarse sin necesidad de coincidir en el tiempo. A través de los foros se puede realizar una serie de intervenciones por escrito sobre diversos temas. Los foros son un medio ideal para publicar mensajes y mantener discusiones públicas. Los foros son una herramienta de comunicación muy valiosa porque permiten compartir y crear conocimientos entre sus usuarios.

Las principales utilidades de los foros radican en que:

- Impulsan al alumno a escribir, a ordenar su pensamiento de manera autónoma.
- Las interacciones resultantes permiten el aprendizaje de manera privilegiada.
- Posibilitan un tratamiento reflexivo que en herramientas sincrónicas no suele ser fácil por problemas de tiempo, número de participantes, etc.
- Educan en el respeto a la opinión ajena, diferente.
- Se aprende a argumentar, habilidad cognitiva de nivel superior.

UN BUEN TUTOR DEBE PRIVILEGIAR LOS FOROS, PORQUE SON UNA HERRAMIENTA DE COMUNICACIÓN MUY VALIOSA QUE PERMITE COMPARTIR Y CREAR CONOCIMIENTOS ENTRE LOS PARTICIPANTES.

- Posibilitan ejercitar el pensamiento crítico y creativo.
- Se enriquecen los roles: en los foros todos podemos ser co-discípulos.

3.2 EL PERFIL DEL TUTOR.

Un buen tutor debe estar calificado en diversas disciplinas: informática, tecnologías de las información y las comunicaciones, pedagogía. Debe también tener conocimientos básicos de los contenidos a impartir.

Aunque generalmente los modelos educativos que trabajan con tutores los utilizan como auxiliares para el aprendizaje y no como docentes principales responsables de los contenidos de los cursos, un tutor tiene que disponer tanto de herramientas pedagógicas (que propicien el aprendizaje del grupo) como de contenido, (que le posibiliten la coherencia en las evaluaciones y la aclaración de dudas). Más allá de que el curso donde el tutor se involucre cuente con expertos en contenidos para el seguimiento de los alumnos, el rol del tutor sigue siendo esencial en el proceso formativo.

Es por ello que el perfil de tutor en el modelo pedagógico que proponemos debe cumplir un conjunto de requisitos:

<u>Pedagógicos</u>

- Experiencia previa en la docencia (obligatorio).
- Experiencia en educación virtual, como docente o alumno (deseable).

UN BUEN TUTOR DEBE ESTAR CALIFICADO EN DIVERSAS DISCIPLINAS: INFORMÁTICA, TECNOLOGÍAS DE LAS INFORMACIÓN Y LAS COMUNICACIONES, PEDAGOGÍA Y DEBE TENER CONOCIMIENTOS BÁSICOS DE LOS CONTENIDOS A IMPARTIR.

Tecnológicos

- Buen manejo de las tecnologías de la información y las comunicaciones (software corrientes, paquetería office, correo electrónico, etc.) (obligatorio).
- Conocimiento previo de la plataforma tecnológica de educación virtual del curso (deseable).

Contenidos del curso

- Universitario de carrera afín al tipo de contenido (obligatorio).
- Conocimientos específicos del tipo de contenido del curso (deseable).

Es importante también que el tutor posea:

- Facilidad para comunicarse, proporcionar confianza y credibilidad.
- Capacidad para liderar un proyecto hacia un objetivo común.
- Facilidad para relacionarse y manejar de conflictos.
- Flexibilidad, adaptabilidad.
- Aptitudes de un agente de cambio.

3.3 LA MISIÓN DEL TUTOR.

La misión del tutor se resume en posibilitar el aprendizaje del alumno y facilitar, a través de estrategias didácticas y comunicativas el proceso de enseñanza.

La misión del tutor presupone un deseo sincero por captar las aspiraciones, motivaciones e inquietudes de cada uno de los participantes que le han sido asignados. Ello implica tener la

UN BUEN TUTOR POSIBILITA EL APRENDIZAJE DEL ALUMNO Y FACILITA, A TRAVÉS DE ESTRATEGIAS DIDÁCTICAS Y COMUNICATIVAS, EL PROCESO DE ENSEÑANZA.

capacidad de la empatía. El mejor tutor no es el que da las mejores respuestas, sino el que ayuda al alumno a encontrarlas.

La actitud de un buen tutor tiene que ser acogedora, flexible, comprensiva, amigable y paciente. Hay que conjuntar la tarea, no siempre fácil, de conjugar un clima democrático, que permita a todos la libre expresión, con un serio control del grupo que impida las divagaciones, pérdidas de tiempo y de ritmo.

EL MEJOR TUTOR NO ES EL QUE DA LAS MEJORES RESPUESTAS, SINO EL QUE AYUDA AL ALUMNO A ENCONTRARLAS.

3.4 LAS HABILIDADES Y COMPETENCIAS REQUERIDAS DE UN TUTOR

Las habilidades y competencias del tutor se describen en las etapas del aprendizaje interactivo diseñadas por Gilly Salmon.

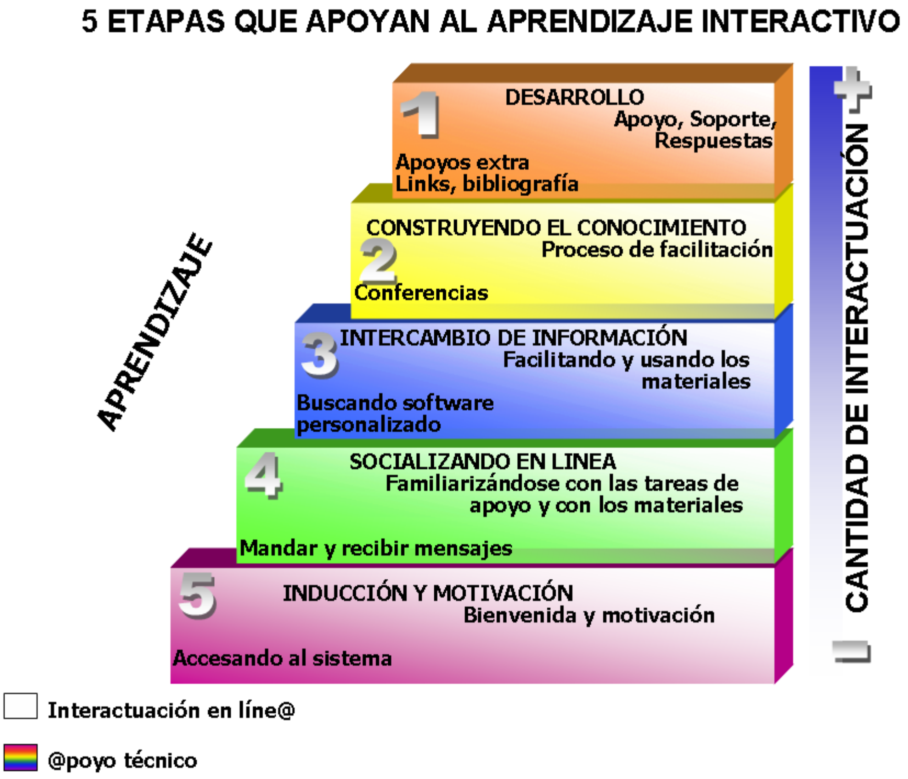

Etapa 1. Inducción y motivación

Esta primera etapa tiene como objetivo llevar a los participantes a incorporarse al grupo. Para ello, el tutor debe destacar sus habilidades de motivación y liderazgo, trasmitiendo a los alumnos seguridad y confianza en que estarán siempre apoyados por él. Debe ser paciente, responder eficientemente todas las inquietudes y recordar que siempre en la primera etapa de un curso se presenta la resistencia al cambio y al uso de las tecnologías, por lo que son básicas las habilidades de dominio de las TIC por parte del tutor.

Etapa 2. Socialización en línea

Un segundo momento es el de la socialización y creación de comunidades. En esta etapa el tutor debe desplegar sus habilidades de comunicación y ser cálido y acogedor. Es necesario “romper el hielo”, buscar empatías, de manera que los participantes sientan confianza para participar naturalmente, compartir ideas y experiencias y se logre el aprendizaje grupal.

Etapa 3. Intercambio de información

En esta etapa los tutores deben apelar, sobre todo, a sus habilidades de conocimiento del tema. Es importante que los alumnos estén apoyados en sus dudas y que se les contextualice el curso con casos prácticos y ejemplos reales.

Etapa 4. Construcción del conocimiento

Esta es la parte más significativa de todo el proceso. En ella los tutores deben desplegar todas sus habilidades, tanto tecnológicas, como de contenidos y pedagógicas.

UN BUEN TUTOR SE DEBE DESTACAR POR SUS HABILIDADES DE MOTIVACIÓN Y LIDERAZGO, TRASMITIENDO A LOS ALUMNOS SEGURIDAD Y CONFIANZA EN QUE ESTARÁN SIEMPRE APOYADOS POR ÉL.

Los tutores en esta etapa son el vehículo que invita a los participantes a discutir los conceptos clave y a enriquecer con sus ideas la información recibida y aclarar cualquier duda.

Etapa 5. Desarrollo

Esta etapa también es primordial, porque en ella los alumnos adquieren las competencias. El tutor se convierte en un agente de cambio, creativo e innovador, que incita a los participantes a reflexionar acerca de lo que puede ser hecho e implementado en su entorno inmediato a partir de los conocimientos adquiridos en el curso.

UN TUTOR ES UN AGENTE DE CAMBIO.

PARTE II

EL TUTOR EN EL CURSO INTEGRACIÓN DEL VIH Y OTRAS ENFERMEDADES INFECCIOSAS PREVALENTES EN EL PRIMER NIVEL DE ATENCIÓN.

EN LA EDUCACIÓN EN SALUD DEBE PRIVILEGIARSE EL ENFOQUE DE LA PROMOCIÓN Y LA PREVENCIÓN.

CAPÍTULO IV. LAS ESTRATEGIAS DE OPERACIÓN DEL CURSO.

4.1 LA EDUCACIÓN EN SALUD. EL PERFIL DE LOS ALUMNOS.

Para la UNESCO, el concepto de la educación en salud, que actualmente abarca un concepto más amplio de promoción de la salud y un nuevo énfasis en la prevención, se concentra cada vez más en el aprendizaje y en el proceso de fortalecimiento de la autonomía.

Como consecuencia, la educación y capacitación de la fuerza de trabajo en salud están abarcando más y más aspectos y ocupando cada vez más seguido una función central dentro de los sectores que desarrollan e implementan las políticas de salud. Como resultado de estos cambios, se han desarrollado nuevas estrategias en áreas en donde la salud y la educación de adultos se entrecruzan. A medida que se forjan nuevas conexiones, se percibe la necesidad de un enfoque sistemático de colaboración entre los dos sectores.

La propuesta formativa que nos ocupa: Curso Integración del y otras enfermedades infecciosas prevalentes en el 1er nivel de atención, se adhiere a esta perspectiva de la UNESCO, en el sentido que relaciona educación en salud y capacitación de adultos. Por estas razones, para un aprendizaje óptimo se recomienda que los participantes de este curso sean profesionales de la salud (médicos, enfermeras, trabajadores comunitarios, gerentes de salud) que presten servicios directos en el 1er. nivel de atención en comunidades postergadas o áreas de riesgo de VIH.

En esta edición del curso, se trabajará con 200 participantes de Nicaragua, Panamá, Guatemala y República dominicana.

4.2 CARACTERÍSTICAS GENERALES DEL CURSO.

El curso Integración del VIH y otras enfermedades infecciosas prevalentes en el 1er nivel de atención es semipresencial (multimodal), porque combina la modalidad virtual con la presencial.

En todo momento los participantes estarán acompañados por los tutores, quienes los guiarán para su mejor aprendizaje. El esquema de trabajo será de 13 a 20 alumnos por tutor y el curso se divide en tres etapas: virtual, presencial y proyectos.

PARA UN APRENDIZAJE ÓPTIMO, LOS ALUMNOS DE ESTE CURSO DEBEN SER PROFESIONALES DE LA SALUD QUE PRESTEN SUS SERVICIOS EN EL 1ER. NIVEL DE ATENCIÓN.

Etapa Virtual

Una primera etapa de este curso, completamente virtual, consiste en:

Tema 1. La integración del VIH en el marco de la renovación de la Atención Primaria de Salud (APS), impartido por especialistas de la Organización Panamericana de la Salud (OPS). Esta parte del curso es virtual y está colocada en una plataforma Moodle.

Tema 2. El manejo clínico del VIH, impartido por docentes de la Universidad Johns Hopkins de los Estados Unidos de América. Esta parte del curso también es virtual y está colocada en la misma plataforma Moodle que el Tema ofertado por la OPS.

Etapa presencial

Una segunda etapa de este curso, en modalidad presencial, consiste en los siguientes momentos:

Tema 3. Generando comunidades de aprendizaje: de la teoría a la práctica. Esta parte del curso es presencial y en esta edición se hará en el Instituto Conmemorativo Gorgas de Panamá.

Etapa de proyectos

La tercera etapa de este curso, en modalidad semipresencial, consiste en:

Elaboración de un proyecto de intervención acción a través del trabajo individual y bajo la supervisión de sus tutores y la presentación de este proyecto ante las autoridades de salud de los países en Talleres Nacionales.

ESTE CURSO TIENE DOS ETAPAS: UNA VIRTUAL Y UNA PRESENCIAL, POR LO QUE ES CONSIDERADO MULTIMODAL.

4.3 MODELO PEDAGÓGICO.

Con este curso los participantes entran a una experiencia pedagógica diferente a la tradicional. Esto se debe a dos razones.

La principal razón es que durante la primera etapa aprenderán de manera virtual a través de la plataforma informática Moodle, software libre, sencillo, amigable y fácil de navegar. El segundo motivo que hace que esta experiencia sea diferente a las habituales es que este curso está diseñado bajo un modelo pedagógico que privilegia que el alumno adquiera competencias para "saber hacer" y "saber ser". Gracias a este modelo pedagógico, al culminar este curso, el participante dominará nuevos procedimientos y habrá modificado actitudes que le posibilitarán ejercer una intervención más eficaz en la integración del VIH y otras enfermedades infecciosas prevalentes en el primer nivel de atención.

Este curso se basa en la metodología de aprender haciendo, por lo que la participación activa es fundamental. Ello facilitará el proceso de intercambio de ideas y de construcción de conocimientos y así los participantes podrán modificar sus esquemas de acción y obtendrán resultados de aprendizaje óptimos.

ESTE CURSO ESTÁ BASADO EN UN MODELO PEDAGÓGICO POR COMPETENCIAS.

4.4 EVALUACIÓN.

Durante el curso los participantes tendrán que entregar al tutor un conjunto de evaluaciones, que llamamos EVIDENCIAS DE APRENDIZAJE y que son trabajos que se solicitarán a los alumnos periódicamente. La suma de estas evaluaciones (evidencias de aprendizaje) constituye el PORTAFOLIO DE EVIDENCIAS.

Hay dos tipos de evaluaciones (evidencias de aprendizaje) que solicitan en este curso:

- Las evidencias cualitativas
- Los cuestionarios.

En el caso de las evidencias de aprendizaje cualitativas (casos, ensayos, resúmenes, etc.) los participantes y el tutor dispondrán de una Lista de Cotejo para saber previamente qué aspectos serán evaluados y qué importancia tiene cada uno de ellos. El tutor revisará cada evaluación (evidencia de aprendizaje) y enviará sus sugerencias a los alumnos, descritas y calificadas en la Lista de Cotejo. De esta manera, los participantes tendrán diversas oportunidades para ir mejorando y perfilando los conocimientos que van adquiriendo.

En el caso de las evidencias de aprendizaje que consisten en cuestionarios automatizados, la propia plataforma Moodle compilará las respuestas y otorgará la calificación de manera automática.

Para ir midiendo los avances, durante la etapa virtual de este curso los alumnos podrán consultar sus calificaciones en la sección "Mis evaluaciones" de la pantalla principal, submenú Herramientas.

Es importante recalcar que TODAS las evidencias de aprendizaje durante la etapa virtual del curso deben ser entregadas a los tutores a través de la plataforma informática. Esta será la única manera de que la plataforma compile las calificaciones. Si un alumno entrega las evidencias a través del correo electrónico o por otra vía, el tutor no tendrá posibilidades de incluir sus calificaciones en Moodle.

DURANTE EL CURSO SE LE SOLICITARÁN A LOS ALUMNOS DOS TIPOS DE EVIDENCIAS DE APRENDIZAJE: CUALITATIVAS Y CUESTIONARIOS.

La acreditación final del curso será la suma de la acreditación de la etapa virtual, la acreditación de la etapa presencial y el puntaje obtenido en el proyecto de intervención acción. Esta acreditación la propondrá el tutor y la otorgará la Coordinación Académica del curso (conformada por la Organización Panamericana de la Salud, la Universidad Johns Hopkins y el Instituto Conmemorativo Gorgas de Estudios de Salud de Panamá).

El portafolio de evidencias del curso y su puntaje correspondiente es el siguiente:

	PUNTAJE MÁXIMO DE ACREDITACIÓN
Tema 1	Total: 20 puntos<br>(5 puntos cada evidencia de aprendizaje semanal)
Tema 2	Total: 20 puntos<br>(5 puntos cada cuestionario de evaluación final semanal)
Actividades finales del curso virtual	Total: 10 puntos<br>(10 puntos la reflexión final)
Tema 3	Total: 15 puntos
Proyecto de intervención acción	Total: 35 puntos
ACREDITACIÓN FINAL	100 PUNTOS

Durante la etapa virtual, el curso presencial en el Instituto Gorgas y la elaboración del proyecto de intervención acción, el tutor será la figura que evaluará al participante (excepto en el caso de los cuestionarios automatizados). El tutor colocará en plataforma (cuando corresponda) la calificación obtenida por el alumno y siempre (excepto en el caso de los cuestionarios automatizados) le brindará nuevas oportunidades de rehacer el trabajo.

LA ACREDITACIÓN FINAL DEL CURSO LA PROPONDRÁ EL TUTOR Y LA OTORGARÁ LA COORDINACIÓN ACADÉMICA DEL CURSO (CONFORMADA POR LA ORGANIZACIÓN PANAMERICANA DE LA SALUD, LA UNIVERSIDAD JOHNS HOPKINS Y EL INSTITUTO CONMEMORATIVO GORGAS DE ESTUDIOS DE SALUD DE PANAMÁ).

El tutor está obligado a entregarle al alumno, en el momento de la calificación, la lista de cotejo con las puntuaciones específicas por rubros y sus comentarios en cada caso cuando no alcanza la máxima puntuación. El tutor siempre le entregará a la Coordinación Académica las calificaciones de los participantes.

Es importante señalar que el alumno debe completar TODOS los temas aunque no los acredite. El tutor deberá velar por ello. Es decir, un alumno no puede entrar al Tema 2 si no ha finalizado el Tema 1, más allá de la calificación obtenida.

Con respecto a la calificación de los proyectos, el tutor, además de ser corresponsable de su elaboración y acompañante del alumno durante su elaboración, deberá evaluar su calidad y entregar también la calificación correspondiente.

Al finalizar el curso (después de la entrega de los proyectos), el tutor conformará el portafolio de evidencias de cada alumno así como redactará dos párrafos con una opinión cualitativa acerca de éste, basándose en su actitud, compromiso y participación durante todo el curso. Esta información el tutor la entregará a la Coordinación Académica del curso (conformada por personas de la Universidad Johns Hopkins, la OPS y el Instituto Gorgas). A partir de esta información, la Coordinación Académica le informará a cada estudiante su situación final, a través de la escala siguiente:

Acreditó con excelencia (más de 90 puntos).
Acreditó (entre 70 y 90 puntos).
No acreditó (menos de 70 puntos).

DURANTE ESTE CURSO EL ALUMNO DEBE COMPLETAR TODOS LOS MÓDULOS AUNQUE NO LOS ACREDITE.

NO PODRÁ ASISTIR A LA FASE PRESENCIAL SIN HABER COMPLETADO LA VIRTUAL.

4.5 CALENDARIO.

FASE VIRTUAL

	FECHA
Inscripciones y Programa de familiarización	Del 24 de marzo al 3 de abril
INICIO DE LA FASE VIRTUAL DEL CURSO	4 de abril
Actividades iniciales	Del 4 al 10 de abril
INICIO DEL TEMA 1	11 de abril
Tema 1 - Semana 1: Los determinantes sociales de la salud, los derechos humanos y el VIH.	Del 11 al 17 de abril
Tema 1 - Semana 2: Los sistemas de salud y su impacto en la respuesta al VIH y enfermedades prevalentes.	Del 18 al 24 de abril
Tema 1 - Semana 3: La renovación de la atención primaria de salud (APS).	Del 25 de abril al 1ro. de mayo
Tema 1 - Semana 4: Modelos de integración del VIH y otras enfermedades infecciosas prevalentes.	Del 2 al 8 de mayo
INICIO DEL TEMA 2	9 de mayo
Tema 2 - Semana 1: Manejo inicial del paciente infectado con VIH	Del 9 al 15 de mayo
Tema 2 - Semana 2: VIH en la mujer embarazada	Del 16 al 22 de mayo
Tema 2 - Semana 3: Infecciones oportunistas y el manejo crónico del VIH	Del 23 al 29 de mayo
Tema 2 - Semana 4: Otras infecciones prevalentes e intervenciones individuales y comunitarias para la prevención y el control de las infecciones trasmitidas por vectores	Del 30 de mayo al 5 de junio
Actividades finales	Del 6 al 11 de junio
FIN DE LA FASE VIRTUAL DEL CURSO	11 de junio

LA FASE VIRTUAL DE ESTE CURSO SERÁ ENTRE EL 4 DE ABRIL Y EL 11 DE JUNIO DE 2011.

FASE PRESENCIAL

	FECHA
TEMA 3 – Cohorte 1	Junio
TEMA 3 – Cohorte 2	Julio
Elaboración de los proyectos de intervención acción	Julio y agosto
Entrega de los proyectos de intervención acción	1ro. de septiembre
Presentación en Talleres Nacionales de los proyectos de intervención acción	Septiembre 2011

LA FASE PRESENCIAL DE ESTE CURSO ABARCA LOS MESES DE JUNIO, JULIO Y AGOSTO DE 2011.

ESTE CURSO TIENE UNA CARGA HORARIA TOTAL DE 150 HORAS.

4.6. PROGRAMA DEL CURSO.

El curso, como se ha mencionado anteriormente, tiene dos fases de contenidos: una virtual y una presencial. La fase virtual está compuesta por Actividades iniciales, Tema 1 y 2 y Actividades finales. La fase presencial está compuesta por el Tema 3. A continuación se describe el Programa de cada una de estas fases y Temas.

GENERALIDADES DEL CURSO

CARGA HORARIA: 150 HORAS

COMPETENCIAS ESPERADAS:

Este curso ha sido diseñado de manera que los participantes, al finalizar las dos etapas (virtual y presencial), sean capaces de haber adquirido las siguientes competencias:

1. Analizar el contexto global en el cual se organiza la prestación de los servicios de salud.

2. Distinguir la organización de los sistemas y servicios de salud en el ámbito nacional y sus especificidades en el ámbito local.

3. Integrar en su lugar de trabajo herramientas que faciliten la promoción, prevención, diagnóstico y tratamiento del VIH, en particular en la prevención de la transmisión materno-infantil del VIH, de la sífilis congénita y de otras enfermedades infecciosas asociadas.

4. Manejar herramientas clínicas para mejorar la provisión de servicios a personas con riesgo de VIH y enfermedades infecciosas prevalentes.

5. Desarrollar propuestas de intervención sostenibles, incluyendo las de capacitación en su contexto de trabajo, para mejorar la eficiencia de la atención.

ACTIVIDADES INICIALES

GENERALIDADES

MODALIDAD: VIRTUAL

CARGA HORARIA: 10 HORAS

DURACIÓN: 1 SEMANA

COMPETENCIAS ESPERADAS:

Que al finalizar la semana los alumnos sean capaces de:

LA FASE VIRTUAL DEL CURSO COMIENZA CON UNA SEMANA DE ACTIVIDADES INICIALES LUEGO DE CONCLUIDO EL PROCESO DE INSCRIPCIONES Y FAMILIARIZACIÓN.

2. Situarse en la problemática de la integración del VIH y otras enfermedades prevalentes en el 1er. Nivel.

DESCRIPCIÓN DE LAS ACTIVIDADES

ACTIVIDAD 1: Llenar el cuestionario de datos iniciales.
ACTIVIDAD 2: Entrar a su perfil y colocar sus datos.
ACTIVIDAD 3: Conocer a su tutor y a sus compañeros.
ACTIVIDAD 4: Navegar el Curso.
ACTIVIDAD 5: Escriba un ensayo corto para identificar los principales factores que afectan la organización e integración de servicios de salud para el control del VIH y otras enfermedades infecciosas prevalentes en su entorno directo de trabajo en el nivel local.

TEMA 1

"LA INTEGRACIÓN DEL VIH EN EL MARCO DE LA RENOVACIÓN DE LA ATENCIÓN PRIMARIA DE SALUD (APS)"

GENERALIDADES

MODALIDAD: VIRTUAL

CARGA HORARIA TOTAL: 30 HORAS

CARGA HORARIA SEMANAL: 7 HORAS Y MEDIA

DURACIÓN: 4 SEMANAS

SEMANA 1: LOS DETERMINANTES SOCIALES DE LA SALUD, LOS DERECHOS HUMANOS Y EL VIH (DEL 11 AL 17 DE ABRIL).

EL TEMA 1 DE LA FASE VIRTUAL DE ESTE CURSO ES IMPARTIDO POR PRESTIGIOSOS DOCENTES DE LA ORGANIZACIÓN PANAMERICANA DE LA SALUD (OPS). SE COMPILAN EN ESTA PARTE DEL CURSO 21 CONFERENCIAS.

SEMANA 2: LOS SISTEMAS DE SALUD Y SU IMPACTO EN LA RESPUESTA AL VIH Y ENFERMEDADES INFECCIOSAS PREVALENTES (DEL 18 AL 24 DE ABRIL).

SEMANA 3: LA RENOVACIÓN DE LA ATENCIÓN PRIMARIA DE SALUD (DEL 25 DE ABRIL AL 1RO. DE MAYO).

SEMANA 4: MODELOS DE INTEGRACIÓN DEL VIH Y OTRAS ENFERMEDADES INFECCIOSAS PREVALENTES(DEL 2 AL 8 DE MAYO).

COMPETENCIAS ESPECÍFICAS

Que al finalizar el tema los alumnos sean capaces de:

1. Distinguir los factores socioculturales y los determinantes sociales que afectan en su contexto las condiciones de salud de la población, en particular las relacionadas con el VIH.
2. Identificar las políticas de protección social en el sector salud y su integración con el VIH y enfermedades infecciosas prevalentes.
3. Analizar las características de la organización de los sistemas y servicios de salud.
4. Analizar el grado de integración de las redes de servicios desde la perspectiva de la fragmentación y segmentación de los sistemas.
5. Reconocer la renovación de la atención primaria como elemento sustantivo en la mejora de la calidad de la atención del VIH y enfermedades infecciosas prevalentes.
6. Formular acciones locales que coadyuven al fortalecimiento de la respuesta de los sistemas de salud frente al VIH y enfermedades infecciosas prevalentes.

DURANTE LAS 4 SEMANAS IMPARTIDAS POR LA OPS, LOS PARTICIPANTES PODRÁN APRENDER ACERCA DE LA INTEGRACIÓN DEL VIH EN EL MARCO DE LA RENOVACIÓN DE LA ATENCIÓN PRIMARIA DE LA SALUD.

DESCRIPCIÓN DEL TEMA 1

Durante la operación del Tema 1, las actividades se estructuran en 3 tipos:

- Actividades obligatorias
- Actividades complementarias
- Actividades de reflexión

Cuando las actividades son OBLIGATORIAS, significa que ellas serán tomadas en cuenta en el portafolio de evidencias y que si el participante no las completa podría influir negativamente en la acreditación del tema y del curso.

Cuando las actividades son COMPLEMENTARIAS O DE REFLEXIÓN significa que el participante no está obligado a realizarlas, sin embargo, se sugieren en tanto buenas prácticas para su aprendizaje.

En el caso de las actividades DE REFLEXIÖN, se recomienda utilizar el Foro para comunicar las reflexiones. Los tutores deben tomar la iniciativa en estos casos y motivar este espacio de discusión, en tanto actividad enriquecedora y que le permitirá a los participantes analizar con más profundidad lo aprendido.

DURANTE LAS 4 SEMANAS IMPARTIDAS POR LA OPS, LAS ACTIVIDADES SE ESTRUCTURAN EN OBLIGATORIAS, COMPLEMENTARIAS Y DE REFLEXIÓN.

Semana 1 – Tema 1 - Los determinantes sociales y el VIH

Actividades obligatorias

1. Escuchar atentamente las tres conferencias:

   CONFERENCIA1. Epidemiologia del VIH en Latinoamérica.
   CONFERENCIA 2. La dimensión social de la salud: los determinantes sociales.
   CONFERENCIA 3. Desigualdades e inequidades de género y etnia.

2. Realizar la lectura Derechos Humanos y VIH. Legislación, política y práctica en cinco paises de centroamérica.

3. Completar la evaluación y enviarla a su tutor.

La evaluación consiste en:

Elaborar un ensayo entre 2 y 3 páginas, formato Word, tipo de letra Arial 11 puntos a doble espacio, para responder el Caso Tierradentro.

Actividades complementarias

CONFERENCIA 1: Desigualdades e inequidades de ingreso y clase social.
CONFERENCIA 2: Pobreza y desigualdades en la utilización de los servicios de salud.
CONFERENCIA 3: El acceso a la salud como derecho humano.

EN LA PRIMERA SEMANA IMPARTIDA POR LA OPS LOS ALUMNOS APRENDERÁN SOBRE LOS DETERMINANTES SOCIALES Y EL VIH.

Actividades de reflexión

Como ACTIVIDADES DE REFLEXIÓN de esta semana lo invitamos a que se conteste a sí mismo estas preguntas. Lo invitamos a escribir en el Foro algunas de sus reflexiones. Le recordamos que la discusión con sus compañeros y tutor siempre será enriquecedora y le permitirá analizar con más profundidad lo aprendido.

1. ¿Es la epidemia de VIH un problema de salud pública en su país? ¿Por qué?
2. ¿Qué poblaciones son las más afectadas? Justifique su respuesta.
3. ¿Cuáles son determinantes sociales más importantes relacionados con el VIH/SIDA?
4. ¿Qué significa alcanzar la equidad de género en la salud?
5. ¿Qué puede hacer Usted desde su posición para favorecer el respeto de los derechos humanos de aquellas personas con el VIH/SIDA?

Material bibliográfico

CIDH. La Convención Americana de Derechos Humanos.
http://www.cidh.oas.org/Basicos/Spanish/Basicos2.htm

CIDH. Protocolo Adicional a la Convención Americana de Derechos Humanos (Protocolo de San Salvador).
http://www.cidh.oas.org/Basicos/Spanish/Basicos4.htm

OPS. Género, diversidad y derechos humanos.
http://www.paho.org/genero_etnicidad/

EN TODOS LOS CASOS SE BRINDA MATERIAL BIBLIOGRÁFICO ADICIONAL.

OMS. Comisión de Determinantes Sociales de la Salud (CSDH). "Determinantes sociales de la salud y equidad en salud: las causas de las causas" en: Lograr la equidad en salud desde las causas iniciales a los resultados justos.

OMS. Comisión sobre Determinantes Sociales de la Salud. Subsanar las desigualdades en una generación. Alcanzar la equidad sanitaria actuando sobre los determinantes sociales de la salud. Resumen Analítico del Informe Final, 2008.

OMS. Determinantes sociales de la salud: los hechos irrefutables.

OMS. Abordar las causas del riesgo y la vulnerabilidad al VIH.

OMS: Derechos humanos y salud. Personas que viven con VIH.

OMS. Subsanar las dificultades. ¿Cómo?

Semana 2 – Tema 1 – Los sistemas de salud y su impacto en la respuesta al VIH y enfermedades prevalentes

Actividades obligatorias

1. Escuche atentamente las tres conferencias:

   CONFERENCIA 1. Los sistemas de salud. Sistemas de salud basados en APS.
   CONFERENCIA 2. Un enfoque integral e integrado. Ámbito de abordaje y modelo asistencial.
   CONFERENCIA 3. La formulación de políticas sanitarias y de protección social.

2. Realice la lectura: Evaluación para el fortalecimiento del sistema de salud al VIH SIDA.
3. Realice la evaluación y envíela a su tutor.

EN LA SEGUNDA SEMANA IMPARTIDA POR LA OPS LOS ALUMNOS APRENDERÁN SOBRE LOS SISTEMAS DE SALUD Y SU IMPACTO EN LA RESPUESTA AL VIH Y OTRAS ENFEREMEDADES INFECCIOSAS PREVALENTES.

La evaluación consiste en:

Revise cuidadosamente el documento Perfil de salud correspondiente a su país. Escriba una reflexión crítica acerca de cómo la situación de salud ahí descrita impacta en el control e integración del VIH y enfermedades infecciosas prevalentes en las acciones y estrategias de salud de su país.

Actividades complementarias

CONFERENCIA 1: Las redes de servicios. Redes integradas.
CONFERENCIA 2: Los perfiles de los sistemas de salud y su impacto en la respuesta al VIH y enfermedades prevalentes. Oportunidades perdidas.

Actividades de reflexión

Como ACTIVIDADES DE REFLEXIÓN de esta semana lo invitamos a que se conteste a sí mismo estas preguntas. Si lo desea, puede escribir en el Foro algunas de sus reflexiones. Le recordamos que la discusión con sus compañeros y tutor siempre será enriquecedora y le permitirá analizar con más profundidad lo aprendido.

1. ¿Promueve su Estado programas de promoción y prevención de la transmisión del VIH para HSH (hombres que tienen sexos con otros hombres, gay, bisexuales, transgénero) y otros)?
2. ¿Existe disponibilidad de prevención post exposición (PEP) para todos los trabajadores de salud de manera gratuita? ¿Han sido capacitados sobre su disponibilidad y uso?
3. ¿Qué poblaciones están o podrían estar con baja cobertura de PTMI?
4. ¿Se hace prueba y seguimiento a la pareja de la mujer embarazada con VIH o SC?
5. ¿Hay mecanismos comunitarios de captación temprana de embarazadas?
6. ¿Cuál es la política nacional sobre el acceso a tratamiento y cuidado? ¿Hay un paquete mínimo de servicios e intervenciones definidas y de acceso universal?
7. ¿Cuáles son las brechas en la cobertura de atención a personas con VIH?

DURANTE LAS CUATRO SEMANAS IMPARTIDAS POR LA OPS LAS EVALUACIONES SIEMPRE SERÁN CUALITATIVAS.

Material bibliográfico

OPS. Análisis del sector salud. Una herramienta para viabilizar la formulación de políticas.

OPS. Redes Integradas de Servicios de Salud. Conceptos, Opciones de Política y Hoja de Ruta para su Implementación en las Américas.

Lineamientos Metodológicos para elaboración de los Perfiles de los Sistemas de Salud.

Informe sobre la salud en el mundo 2000 - Mejorar el desempeño de los sistemas de salud, Capítulo 1.

Perfiles de salud de todos los países de la región en:

http://www.lachealthsys.org/index.php?option=com_content&task=view&id=373&Itemid=450

EN LA TERCERA SEMANA IMPARTIDA POR LA OPS LOS ALUMNOS APRENDERÁN SOBRE LA RENOVACIÓN DE LA APS.

Semana 3 – Tema 1 - La renovación de la atención primaria de salud (APS).

Actividades obligatorias

1. Escuche atentamente las cuatro conferencias:

   CONFERENCIA 1. Programas básicos de salud: el ciclo de vida saludable.
   CONFERENCIA 2. La gestión del cambio para la renovación de la APS.
   CONFERENCIA 3. La importancia del recurso humano en la APS.
   CONFERENCIA 4: La participación comunitaria. Intersectorialidad.

2. Realice la evaluación y envíela a su tutor.

La evaluación consiste en:

Elaborar un ensayo entre 2 y 3 páginas, formato Word, tipo de letra Arial 11 puntos a doble espacio, para responder el Caso "Estrategias de gestión e integración de programas de atencion de salud. Una nueva orientación de la atencion de salud para el Centro de Atención Primaria de Salud de los Lapachos".

Actividad complementaria

CONFERENCIA 1: La educación permanente. Construyendo liderazgos para la gestión.

Actividades de reflexión

Como ACTIVIDADES DE REFLEXIÓN de esta semana lo invitamos a que se conteste a sí mismo estas preguntas. Si lo desea, puede escribir en el Foro algunas de sus reflexiones. Le recordamos que la discusión con sus compañeros y tutor siempre será enriquecedora y le permitirá analizar con más profundidad lo aprendido.

1. ¿Cuál es la conexión entre la participación comunitaria, los determinantes de la salud y la Atención Primaria en Salud?
2. Indique cuatro ejes principales que articulan el trabajo entre el sistema de salud y lo s determinantes de la salud.
3. Mencione y describa una iniciativa para promover la participación comunitaria en la prevención de VIH/SIDA y sus factores de riesgo.
4. ¿Cómo debe realizarse fundamentalmente la prevención del VIH en la población de jóvenes en América Latina?
5. ¿A qué está asociada principalmente la vulnerabilidad biológica al VIH?
6. Describa la importancia del uso de las tecnologías de la información y las comunicaciones para la prevención del VIH y otras enfermedades infecciosas prevalentes.

AUNQUE LAS ACTIVIDADES COMPLEMENTARIAS NO SON OBLIGATORIAS, SE RECOMIENDAN DE MANERA IMPORTANTE PARA GARANTIZAR UN BUEN APRENDIZAJE.

Material bibliográfico

OPS/OMS. La Renovación de la APS en las Américas, documento de posición de la Organización Panamericana de la Salud/ Organización Mundial de la Salud (OPS/OMS).

OPS/OMS. Palabras de la Dra. Mirta Roses, Directora OPS/OMS en el cierre de la Conferencia Internacional Buenos Aires

Semana 4 – Tema 1 – La integración del VIH en el marco de la renovación de la atención primaria de salud (APS)

Actividades obligatorias

1. Escuche atentamente las tres conferencias:

   CONFERENCIA 1. Modelos de integración del VIH en los servicios.
   CONFERENCIA 2. Prevención del VIH en adolecentes y jóvenes.
   CONFERENCIA 3. Promoviendo la salud sexual y reproductiva en la APS.

2. Realice la evaluación y envíela a su tutor.

La evaluación consiste en:

Elaborar un ensayo entre 2 y 3 páginas, formato Word, tipo de letra Arial 11 puntos a doble espacio, para responder el Caso "Oportunidades perdidas y estrategias de intervención para la prevención del VIH en adolescentes".

Actividades complementarias

CONFERENCIA 1: Servicios farmacéuticos en la APS.
CONFERENCIA 2: Uso de la información para la toma de decisiones.

EN LA CUARTA SEMANA IMPARTIDA POR LA OPS LOS ALUMNOS APRENDERÁN SOBRE LA INTEGRACIÓN DEL VIH EN EL MARCO DE LA RENOVACIÓN DE LA APS.

Actividades de reflexión

Como ACTIVIDADES DE REFLEXIÓN de esta semana lo invitamos a que se conteste a sí mismo estas preguntas. Si lo desea, puede escribir en el Foro algunas de sus reflexiones. Le recordamos que la discusión con sus compañeros y tutor siempre será enriquecedora y le permitirá analizar con más profundidad lo aprendido.

1. ¿Cómo es la entrega de medicamentos en su entorno inmediato?
2. ¿En el servicio de salud donde trabaja existen servicios farmacéuticos o solamente entrega de medicamentos?
3. ¿Se acompaña la dispensación de medicamentos con información al paciente?
4. ¿Participa el servicio farmacéutico en campañas educativas o en los programas
   de adherencia a los tratamientos?
5. ¿Crees que la propuesta presentada sobre los servicios farmacéuticos en el contexto de la Atención Primaria de salud centrados en las personas puede contribuir a mejorar el control del VIH? ¿Por qué?
6. ¿Cómo podría mejorar desde los servicios de salud, incluidos los servicios farmacéuticos, los procesos de apoyo relacionados con el suministro de medicamentos?

Material bibliográfico

OPS/OMS. Iniciativa regional para la eliminación de la transmisión materno-infantil del VIH y de la sífilis congénita en América Latina y el Caribe: Estrategia de monitoreo regional.

OPS/OMS. Vinculación de programas y servicios de salud sexual y reproductiva, género y de prevención de VIH e ITS.

OPS/OMS. Proyecto para la Provisión de Atención Integral a los hombres gay y otros hombres que tienen sexo con hombres (HSH) en América Latina y el Caribe.

OPS/OMS. Infecciones de transmisión sanguínea o sexual entre las personas que se inyectan drogas y sus parejas en las Américas. Manual para profesionales de la salud.

OPS/OMS. Indicadores de alerta temprana de farmacorresistencia del VIH.

SE RECOMIENDA QUE LAS ACTIVIDADES DE REFLEXIÓN SE CONVIERTAN EN UN ESPACIO IMPORTANTE PARA EL DEBATE Y LA CONSTRUCCIÓN DE UN CONOCIMIENTO COLECTIVO.

TEMA 2

"MANEJO CLÍNICO DEL VIH Y ENFERMEDADES INFECCIOSAS PREVALENTES EN EL PRIMER NIVEL DE ATENCIÓN"

GENERALIDADES

MODALIDAD: VIRTUAL

CARGA HORARIA TOTAL: 30 HORAS

CARGA HORARIA SEMANAL: 7 HORAS Y MEDIA

DURACIÓN: 4 SEMANAS

SEMANA 1: MANEJO INICIAL DEL PACIENTE INFECTADO CON VIH (DEL 9 AL 15 DE MAYO).

SEMANA 2: VIH EN LA MUJER EMBARAZADA (DEL 16 AL 22 DE MAYO).

SEMANA 3: INFECCIONES OPORTUNISTAS Y EL MANEJO CRÓNICO DEL VIH (DEL 23 AL 29 DE MAYO).

SEMANA 4: OTRAS INFECCIONES PREVALENTES, Y TAMBIÉN ACERCA DE LAS INTERVENCIONES INDIVIDUALES Y COMUNITARIAS PARA LA PREVENCIÓN Y EL CONTROL DE LAS INFECCIONES TRASMITIDAS POR VECTORES (DEL 30 DE MAYO AL 5 DE JUNIO).

EL TEMA 2 DE LA FASE VIRTUAL DE ESTE CURSO ES IMPARTIDO POR PRESTIGIOSOS DOCENTES Y COLABORADORES DE LA UNIVERSIDAD JOHNS HOPKINS (UJH). SE COMPILAN EN ESTA PARTE DEL CURSO 21 CONFERENCIAS.

COMPETENCIAS ESPECÍFICAS

Que al finalizar el tema los alumnos sean capaces de:

1. Aplicar los principios básicos de la prevención y evaluación inicial del VIH a los cuidados primarios.
2. Reconocer la importancia de hacer la prueba de VIH a toda mujer embarazada como un paso indispensable para la prevención de la transmisión del VIH al infante y la evaluación de la familia.
3. Reconocer como cuidados primarios básicos el tratamiento adecuado de las infecciones de transmisión sexual, la planificación familiar y la detección de la tuberculosis y su impacto en la epidemia del VIH.
4. Manejar herramientas básicas que mejoren la calidad de los servicios de salud a pacientes con VIH u otras infecciones prevalentes.
5. Proponer estrategias que mejoren la capacidad local de detectar el VIH a tiempo y conducir la evaluación básica del paciente infectado en el contexto familiar.

DESCRIPCIÓN DEL TEMA 2

Durante la operación del Tema 2, las actividades se estructuran en 4 tipos:

- Evaluación inicial de la semana
- Conferencias de la semana
- Evaluación final de la semana
- Actividades de reflexión

DURANTE LAS CUATRO SEMANAS IMPARTIDAS POR LA UJH LAS ACTIVIDADES SE ESTRUCTURAN EN CUATRO TIPOS:

EVALUACIÓN INICIAL DE LA SEMANA.

CONFERENCIAS.

ACTIVIDADES DE REFLEXIÓN.

EVALUACIÓN FINAL DE LA SEMANA.

La evaluación inicial de cada semana, las conferencias y la evaluación final son actividades OBLIGATORIAS.

Las actividades DE REFLEXIÓN, al igual que anteriormente, no son obligatorias para el participante, sin embargo, se sugieren en tanto buenas prácticas para su aprendizaje.

Al igual que en el Tema 1, en el caso de las actividades DE REFLEXIÖN, se recomienda utilizar el Foro para comunicar las reflexiones. Los tutores deben tomar la iniciativa en estos casos y motivar este espacio de discusión, en tanto actividad enriquecedora y que le permitirá a los participantes analizar con más profundidad lo aprendido.

Semana 1 – Tema 2 – Manejo inicial del paciente infectado con VIH

Conferencias obligatorias

CONFERENCIA 1. Mecanismos de transmisión e historia natural de la infección por VIH.
CONFERENCIA 2. Diagnóstico temprano de la infección por VIH.
CONFERENCIA 3. Evaluación inicial del paciente infectado con VIH.
CONFERENCIA 4. Promoción de la salud en personas con VIH.
CONFERENCIA 5. Infecciones de transmisión sexual y VIH.

Actividades de reflexión

Como ACTIVIDADES DE REFLEXIÓN de esta semana lo invitamos a que se conteste a sí mismo estas preguntas. Lo invitamos a escribir en el Foro algunas de sus reflexiones. Le recordamos que la discusión con sus compañeros y tutor siempre será enriquecedora y le permitirá analizar con más profundidad lo aprendido.

EN LA PRIMERA SEMANA IMPARTIDA POR LA UJH LOS ALUMNOS APRENDERÁN ACERCA DEL MANEJO INICIAL DEL PACIENTE INFECTADO CON VIH.

1. ¿Cuáles son las barreras al diagnóstico temprano del VIH en su clínica y/o en su país?
2. ¿De qué manera crea el estigma una barrera para recibir pruebas del VIH y atención médica? Considere el estigma desde el punto de vista de los pacientes y de los proveedores.
3. ¿Ha sido testigo a la discriminación o parcialidad inconsciente contra los pacientes con VIH o que están a riesgo de contraer el VIH? La parcialidad inconsciente es una creencia profundamente arraigada a cerca de un grupo de individuos que puede afectar el comportamiento de manera no deliberada. Un ejemplo de parcialidad inconsciente es una persona que cree que las personas con VIH merecen su infección debido a sus acciones.
4. En su clínica, ¿todos quienes se hacen las pruebas para los ITS también se hacen la prueba del VIH y vice versa? Si no es así, ¿cuáles son algunas de las intervenciones prácticas que pueden cambiar esto?

EN TODOS LOS CASOS SE OFRECE MATERIAL BIBLIOGRÁFICO ADICIONAL.

<u>Material bibliográfico</u>

CDC en Español: VIH/SIDA
http://www.cdc.gov/hiv/spanish/
Información básica sobre el VIH y el SIDA
http://www.cdc.gov/hiv/spanish/topics/basic/index.htm
Preguntas y Respuestas acerca del VIH/SIDA
http://www.cdc.gov/hiv/spanish/resources/qa/index.htm
Transmisión del VIH
http://www.cdc.gov/hiv/spanish/resources/qa/transmission.htm
¿Cómo prevenir la transmisión ocupacional del VIH en el personal de cuidado de salud?
http://www.cdc.gov/hiv/spanish/resources/factsheets/hcwprev.htm

Semana 2 – Tema 2- VIH en la mujer embarazada

Conferencias obligatorias

CONFERENCIA 1. Prueba de VIH en los cuidados prenatales y notificación de pareja.
CONFERENCIA 2. Planificación familiar en pacientes con VIH.
CONFERENCIA 3. Uso de antirretrovirales y complicaciones relacionadas al VIH durante el embarazo .
CONFERENCIA 4. Manejo de parto y del infante expuesto.
CONFERENCIA 5. Profilaxis post-exposición (PPE) para el VIH.

Actividades de reflexión

Como ACTIVIDADES DE REFLEXIÓN de esta semana lo invitamos a que se conteste a sí mismo estas preguntas. Lo invitamos a escribir en el Foro algunas de sus reflexiones. Le recordamos que la discusión con sus compañeros y tutor siempre será enriquecedora y le permitirá analizar con más profundidad lo aprendido.

1. En los EEUU, la transmisión materno infantil del VIH ha disminuido a menos del 2%. En Centroamérica, aunque la terapia antirretroviral está disponible para todos, la transmisión materno infantil sigue siendo un problema importante de salud pública. ¿A qué se debe esto?
2. Si usted fuera el Ministerio de Salud, ¿cuál sería su recomendación para disminuir la transmisión del VIH a los recién nacidos?
3. Si usted fuera el director de su clínica, ¿qué recomendaría?
4. En su posición, ¿qué podría hacer para mejorar la prevención de la transmisión del VIH a los recién nacidos?

EN LA SEGUNDA SEMANA IMPARTIDA POR LA UJH LOS ALUMNOS APRENDERÁN ACERCA DEL VIH EN LA MUJER EMBARAZADA.

5. Caso: Una mujer de 19 años de edad se presenta a la clínica durante el segundo trimestre del embarazo. Se realiza la prueba del VIH y resulta positiva. ¿Cómo le revelaría los resultados al paciente y qué recomendaciones importantes haría para que tenga un parto sin riesgo?
6. Al enterarse de su resultado, una mujer comienza a llorar inconsolablemente y dice que su marido la mataría si se enterara de que es VIH positiva. ¿Qué la aconsejaría? ¿Qué tan importante es evaluar el riesgo de violencia domestica para determinar si se debe notificar el resultado positivo a su pareja y la manera de hacerlo? ¿Se debe proteger la confidencialidad? ¿De quién?

Material bibliográfico

La infancia y el SIDA (OMS)
Ampliar la prevención, el diagnóstico y el tratamiento de los lactantes y los niños con el VIH (OMS)
Aunque una madre tenga el VIH, el bebé no tiene por qué contraerlo

Semana 3 – Tema 2 - Infecciones oportunistas y el manejo crónico del VIH

Conferencias obligatorias

CONFERENCIA 1. Coinfección Tuberculosis y VIH.
CONFERENCIA 2. Neumonía por Pneumocystis jirovecii (PcP) en pacientes infectados por el VIH.
CONFERENCIA 3. Criptococosis y Toxoplasmosis.
CONFERENCIA 4. Terapia ARV 2011: de las guías a la práctica diaria.
CONFERENCIA 5. Toxicidad, adherencia y monitoreo de laboratorio.

EN LA TERCERA SEMANA IMPARTIDA POR LA UJH LOS ALUMNOS APRENDERÁN ACERCA DE LAS INFECCIONES OPORTUNISTAS Y EL MANEJO CRÓNICO DEL VIH.

Pan American Health Organization

Actividades de reflexión

Como ACTIVIDADES DE REFLEXIÓN de esta semana lo invitamos a que se conteste a sí mismo estas preguntas. Lo invitamos a escribir en el Foro algunas de sus reflexiones. Le recordamos que la discusión con sus compañeros y tutor siempre será enriquecedora y le permitirá analizar con más profundidad lo aprendido.

1. La OMS/OPS ha recomendado que los programas de tratamiento del VIH y TB debieran integrar sus servicios. ¿Cuáles son las barreras para garantizar que todos los pacientes con TB se hagan la prueba del VIH y que todos los pacientes con VIH se hagan la prueba de la TB?
2. La OMS/OPS recientemente cambió las recomendaciones para iniciar terapia antirretroviral en pacientes con un recuento de CD4 superior (<350 en vez de <200 como sugerían los directrices anteriores). ¿Cuáles son las ventajas de iniciar TARV temprano? ¿Hay desventajas? ¿Qué se puede hacer para reducirlas al mínimo?
3. Se toma un promedio de seis años desde el momento de la infección por el VIH hasta tener síntomas del VIH/SIDA. ¿Qué proporción de pacientes con VIH obtienen su primer diagnóstico cuando se presentan con síntomas de una infección oportunista (IO) grave? ¿Cómo podría motivar a los pacientes y la comunidad a que pidan o soliciten la prueba del VIH antes de caer enfermos? ¿Cómo se podría motivar a los proveedores a que realicen las pruebas de VIH en todos los pacientes?

EN TODOS LOS CASOS SE OFRECEN ACTIVIDADES DE REFLEXIÓN, LAS CUALES SON MUY IMPORTANTES PARA EL APRENDIZAJE. ESTAS SE DESARROLLAN EN EL FORO

Material bibliográfico

La tuberculosis: guía para adultos y adolescentes con el VIH

http://www.cdc.gov/hiv/spanish/resources/brochures/oi_tb.htm

Usted puede prevenir la PcP en adultos y adolescentes.

http://www.cdc.gov/hiv/spanish/resources/brochures/pcpb.htm

Usted puede prevenir la toxoplasmosis

http://www.cdc.gov/hiv/spanish/resources/brochures/toxo.htm

Semana 4 – Tema 2- Otras infecciones prevalentes, y también acerca de las intervenciones individuales y comunitarias para la prevención y el control de las infecciones trasmitidas por vectores

Conferencias obligatorias

CONFERENCIA 1. Dengue.
CONFERENCIA 2. Otras infecciones endémicas: La Malaria.
CONFERENCIA 3. Histoplasmosis.
CONFERENCIA 4. Coinfección Hepatitis y VIH.
CONFERENCIA 5. Parásitos en pacientes con VIH / SIDA.
CONFERENCIA 6. Intervenciones individuales y comunitarias para la prevención y el control de las infecciones transmitidas por vectores.

Actividades de reflexión

Como ACTIVIDADES DE REFLEXIÓN de esta semana lo invitamos a que se conteste a sí mismo estas preguntas. Lo invitamos a escribir en el Foro algunas de sus reflexiones. Le recordamos que la discusión con sus compañeros y tutor siempre será enriquecedora y le permitirá analizar con más profundidad lo aprendido.

EN LA CUARTA SEMANA IMPARTIDA POR LA UJH LOS ALUMNOS APRENDERÁN ACERCA DE OTRAS INFECCIONES PREVALENTES, Y TAMBIÉN ACERCA DE LAS INTERVENCIONES INDIVIDUALES Y COMUNITARIAS PARA LA PREVENCIÓN Y EL CONTROL DE LAS INFECCIONES TRASMITIDAS POR VECTORES.

1. ¿Qué intervenciones pueden implementar los funcionarios de salud pública, a nivel comunitario, para prevenir las enfermedades transmitidas por vectores y las enfermedades parasitarias?
2. ¿Qué intervenciones se pueden implementar a nivel individual para prevenir enfermedades transmitidas por vectores y las enfermedades parasitarias?
3. ¿Cuáles son los obstáculos a la erradicación de la malaria en su área?
4. ¿Qué se puede hacer para prevenir las muertes por dengue?

Material bibliográfico

La coinfección por el virus de la hepatitis C
http://www.cdc.gov/hiv/spanish/resources/qa/coinfection.htm
Alimentos y agua sin riesgos: Guía para personas infectadas con el VIH
http://www.cdc.gov/hiv/spanish/resources/brochures/food.htm

ACTIVIDADES FINALES

MODALIDAD: VIRTUAL

CARGA HORARIA: 10 HORAS

DURACIÓN: 1 SEMANA

LA FASE VIRTUAL DEL CURSO CONCLUYE CON UNA SEMANA DE ACTIVIDADES FINALES.

ACTIVIDADES

ACTIVIDAD 1: Escribir y enviar a su tutor un ensayo acerca de ¿cuáles son los principales factores que afectan la organización e integración de servicios de salud para el control del VIH y otras enfermedades infecciosas prevalentes en su entorno directo de trabajo en el nivel local y qué podría hacerse para mejorar la integración?
ACTIVIDAD 2: Definir el tema de su proyecto de intervención acción, hacer un resumen y enviarlo a su tutor.
ACTIVIDAD 3: Evaluar la etapa virtual de este curso.

TEMA 3

GENERANDO COMUNIDADES DE APRENDIZAJE: DE LA TEORÍA A LA PRÁCTICA.

MODALIDAD: PRESENCIAL

CARGA HORARIA TOTAL: 40 HORAS

DURACIÓN: 1 SEMANA

COMPETENCIAS ESPECÍFICAS

Que al finalizar el tema los alumnos sean capaces de:

1. Aplicar los conocimientos y habilidades adquiridos a través de la Fase Virtual del curso.
2. Utilizar herramientas que contribuyan al mejor desarrollo de los proyectos de intervención acción, incluyendo aquellas que puedan facilitar la consecución de recursos para su implementación.
3. Concretar proyectos locales que coadyuven a la integración del VIH y otras enfermedades infecciosas en el 1er. Nivel.

LA FASE PRESENCIAL DEL CURSO TIENE DOS MOMENTOS: EL TEMA 3 Y LA ELABORACIÓN DE LOS PROYECTOS DE INTERVENCIÓN ACCIÓN.

PROYECTOS DE INTERVENCIÓN ACCIÓN

MODALIDAD: PRESENCIAL

CARGA HORARIA: 30 HORAS

DURACIÓN: 2 MESES (8 SEMANAS QUE COMPRENDEN LOS MESES JULIO Y AGOSTO DE 2011)

CARGA HORARIA SEMANAL: 3.7 HORAS

COMPETENCIAS ESPERADAS:

1. Presentar y defender ante las autoridades de salud de los países proyectos locales que coadyuven a la integración del VIH y otras enferemedades infecciosas prevalentes en el 1er. Nivel de atención.

INFORMACIÓN IMPORTANTE DE LOS PROYECTOS

Como se ha mencionado anteriormente, como parte de las actividades del curso "Integración del VIH y otras enfermedades prevalentes en el primer nivel de atención" los alumnos deberán formular un proyecto de intervención orientado a aplicar los principales conceptos, metodologías y herramientas que aprenderán a lo largo del curso.

LOS PROYECTOS SERÁN PRESENTADOS EN TALLERES NACIONALES ANTE LAS AUTORIDADES DE SALUD DE LOS PAÍSES.

Pan American Health Organization

Para estos efectos los participantes recibirán el apoyo y monitoreo del tutor que los ha acompañado a lo largo del desarrollo del curso en su etapa virtual, para resolver dudas metodológicas de la formulación del proyecto, la adecuación de sus contenidos y el diseño de estrategias de implementación para el proyecto a desarrollar.

Los proyectos deberán basarse en la identificación de un problema o situación en el ámbito de acción de cada participante en su país, susceptible de ser modificado con la intervención propuesta.

Las intervenciones deberán ser factibles en términos de su implementación y viables desde el punto de vista técnico, financiero y de políticas locales de salud. Al mismo tiempo deberán tener resultados medibles y evaluables en términos de mejoría en el volumen y/o calidad de los servicios y atención de salud ofertada a la población.

EL TUTOR ES CO-RESPONSABLE DE LA ELABORACIÓN DE LOS PROYECTOS DE INTERVENCIÓN ACCIÓN.

4.7 LA PLATAFORMA TECNOLÓGICA.

La conocida plataforma Moodle es la utilizada como medio de enseñanza durante la fase virtual del curso.

El Moodle de este curso está disponible dentro de los cursos de del Campus Virtual de Panamá en la dirección web http://cursospaises.campusvirtualsp.org

Para los efectos del curso y mejor utilización de esta plataforma se ha elaborado una interface amigable, la cual se navega de arriba hacia abajo según el menú que está a la izquierda en la pantalla principal.

En esta plataforma están colocadas todas las actividades de aprendizaje para los alumnos referidas a los Temas 1 y 2 de este curso.

Además, hay información relacionada con el tema 3 y con los proyectos de intervención acción. Se asocian a esta plataforma también un conjunto de herramientas para facilitar el aprendizaje.

Al inicio del curso se incluye un período de inscripciones con un programa de familiarización que pone a disposición de los alumnos el Campus Virtual de Salud Pública de la OPS. También hay una semana de actividades iniciales que permitirá a los participantes adaptarse al uso de la plataforma informática y sus herramientas.

LA PLATAFORMA TECNOLÓGICA DEL CURSO VIRTUAL ES MOODLE.

EL CURSO ESTÁ ALOJADO EN EL SERVIDOR DEL CAMPUS VIRTUAL DE SALUD PÚBLICA DE LA OPS.

<u>VISTA GENERAL DE LA PLATAFORMA</u>

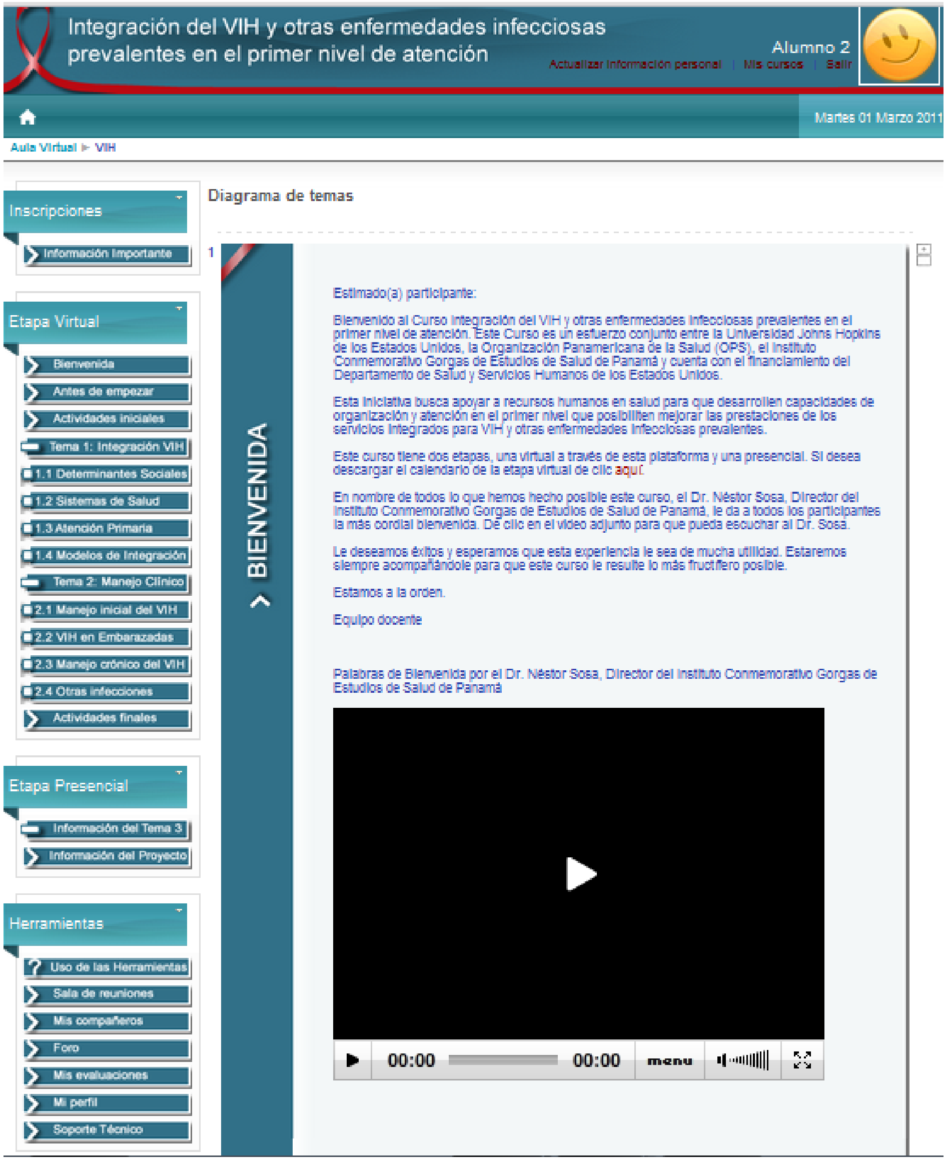

EL CURSO TIENE UN DISEÑO WEB SENCILLO Y AMIGABLE.

MENÚ INSCRIPCIONES – SUBMENÚ INFORMACIÓN IMPORTANTE

En el Menú Inscripciones de la Plataforma Moodle el participante encontrará el Submenú Información Importante, el cual informa al alumno acerca del programa de Familiarización que brinda el Campus Virtual de la OPS y que estará disponible durante el período de inscripciones.

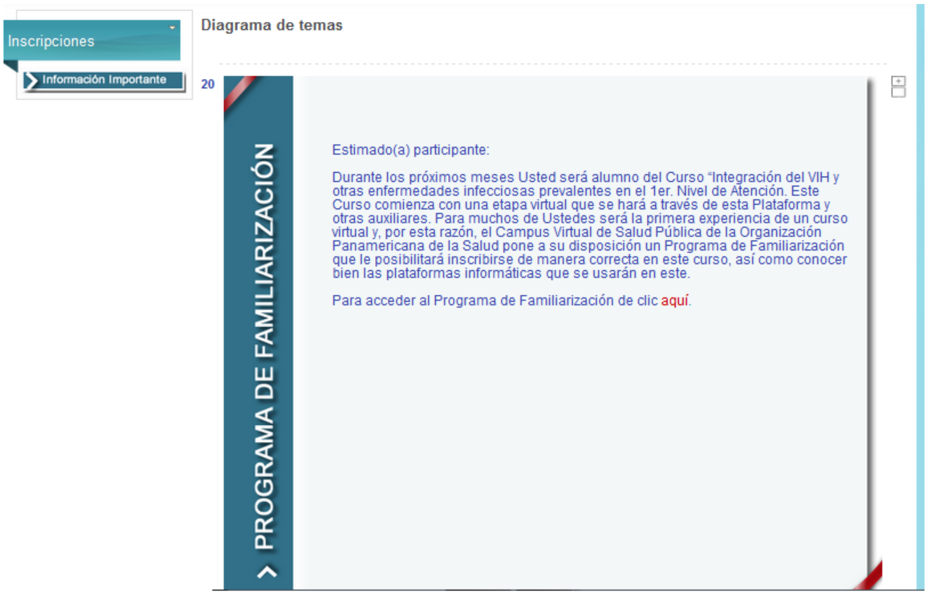

LA NAVEGACIÓN WEB DEL CURSO SE HACE A TRAVÉS DEL SISTEMA DE MENÚS COLOCADOS A LA IZQUIERDA, DE ARRIBA HACIA ABAJO.

MENÚ ETAPA VIRTUAL

En este Menú se despliegan un conjunto de submenús que competen a TODA la actividad virtual del curso. El orden lógico de navegación es de arriba hacia abajo.

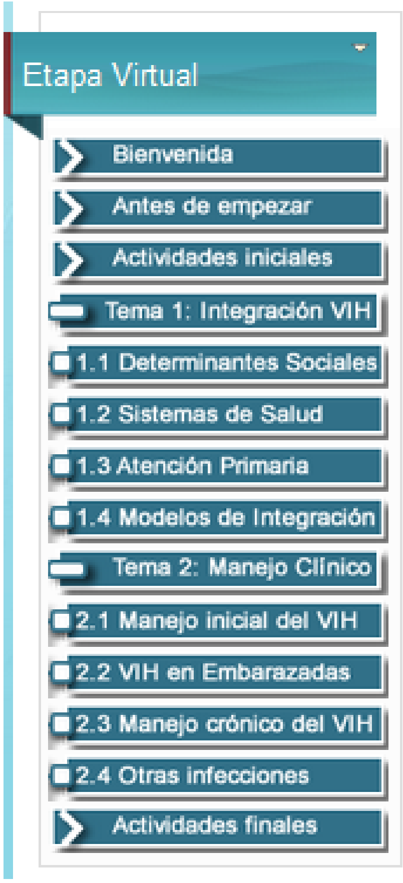

ALGUNOS MENÚS DESPLIEGAN SUBMENÚS.

SUBMENÚ BIENVENIDA

En este submenú se encuentra información que el Director del Instituto Gorgas brinda en nombre del Grupo de Desarrollo Curricular de este curso. La información brindada por el Dr. Néstor Sosa está en formato video. También se encuentran en esta sección el Calendario y Programa del curso.

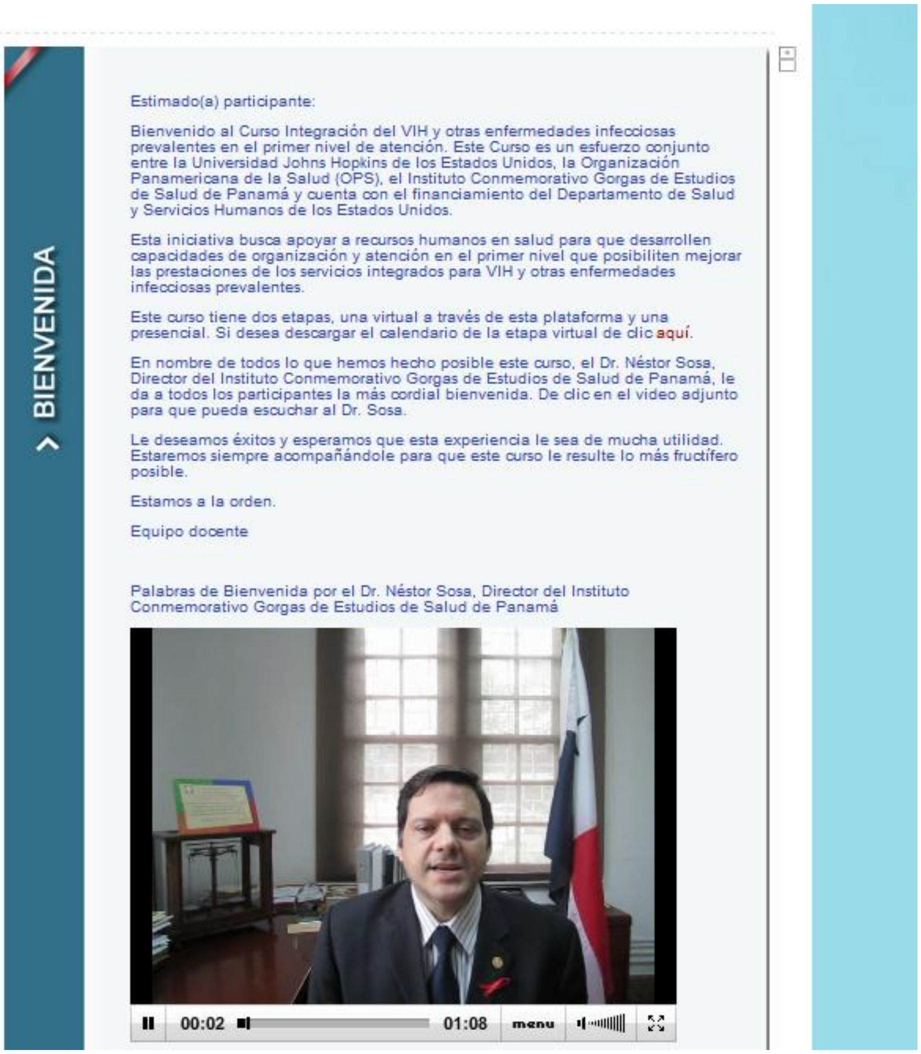

LA PLATAFORMA DEL CURSO TIENE INCORPORADOS LECTORES DE VIDEOS.

SUBMENÚ ANTES DE EMPEZAR

En el Submenú antes de empezar, se brindan un conjunto de informaciones generales sobre el curso, referentes a su modelo pedagógico, forma de evaluación, etc.

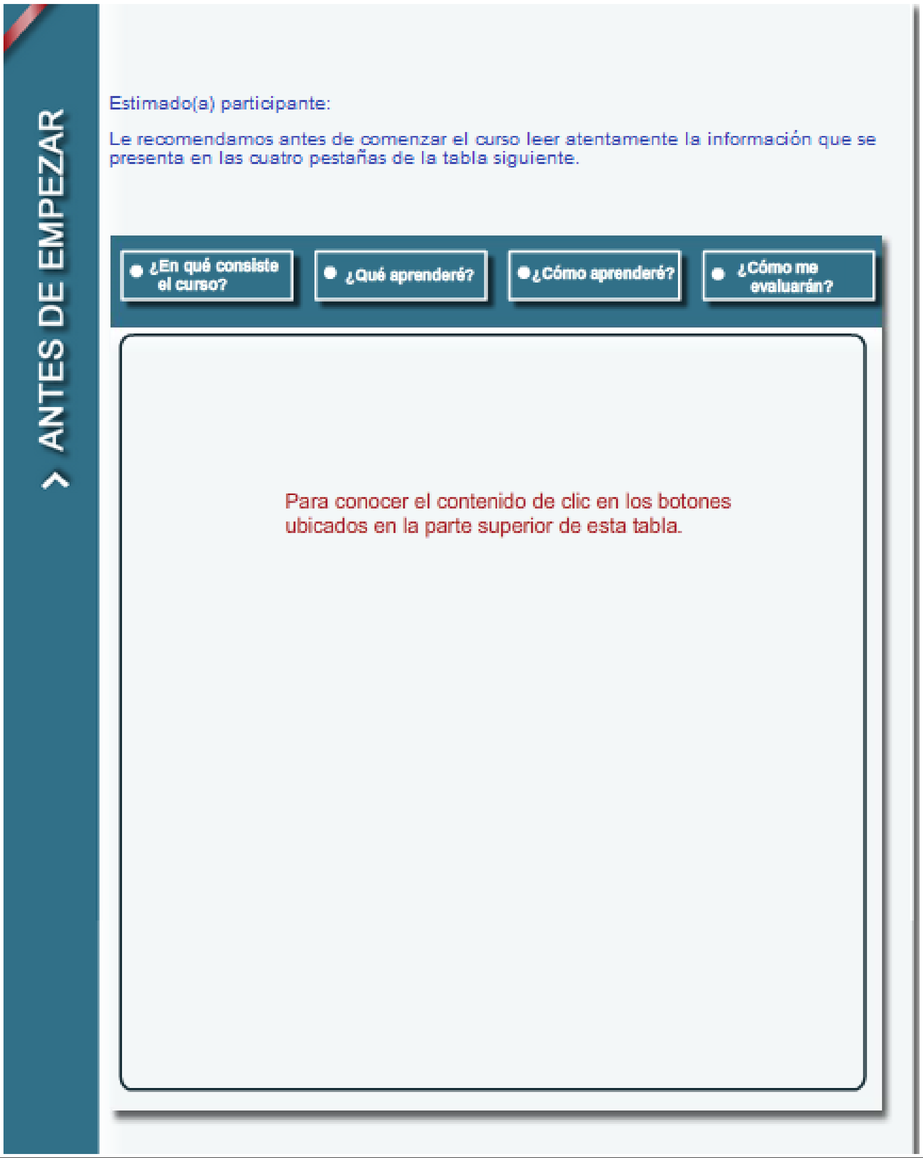

EN LOS SUBMENÚS EN FORMA DE TABLA, NÓTESE QUE SE DEBE NAVEGAR CADA UNA DE SUS PESTAÑAS.

SUBMENÚ ACTIVIDADES INICIALES

Con el submenú actividades iniciales comienza realmente la actividad pedagógica del curso. Se presentan un conjunto de actividades que deben ser completadas antes de entrar en los Temas a tratar por la OPS y la UJH. Nótese que para navegar entre las actividades deben usarse las flechas que se presentan en la parte de abajo de la tabla.

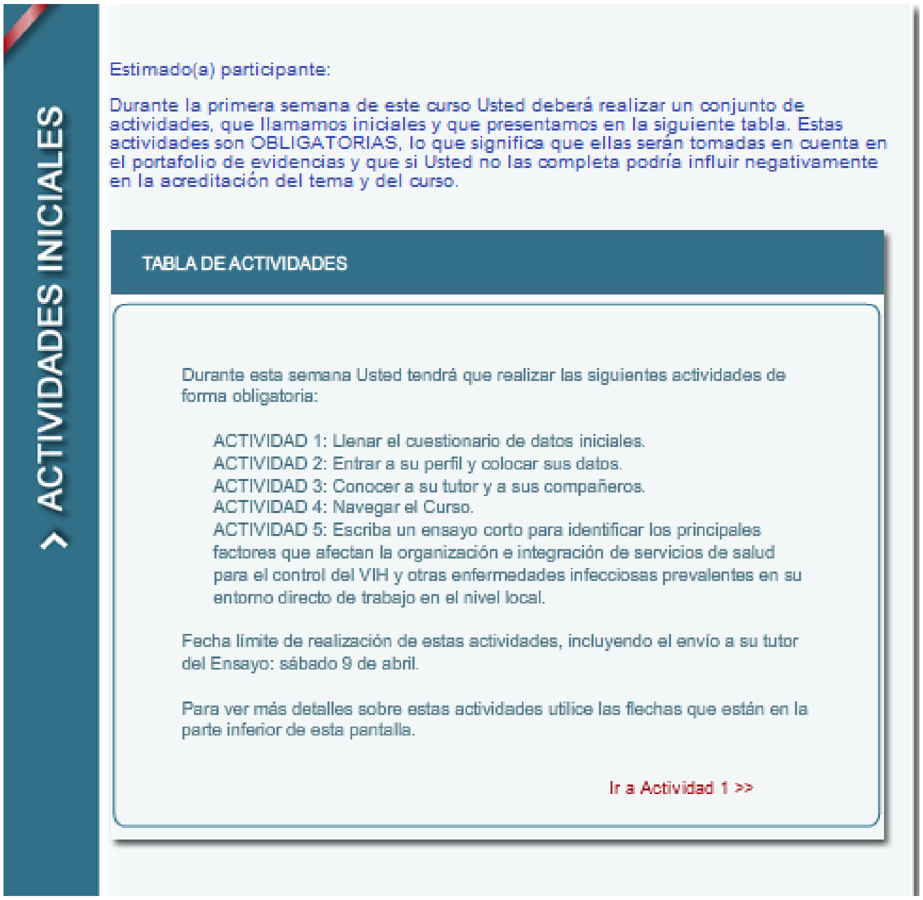
ACTIVIDADES INICIALES

Estimado(a) participante:

Durante la primera semana de este curso Usted deberá realizar un conjunto de actividades, que llamamos iniciales y que presentamos en la siguiente tabla. Estas actividades son OBLIGATORIAS, lo que significa que ellas serán tomadas en cuenta en el portafolio de evidencias y que si Usted no las completa podría influir negativamente en la acreditación del tema y del curso.

TABLA DE ACTIVIDADES

Durante esta semana Usted tendrá que realizar las siguientes actividades de forma obligatoria:

ACTIVIDAD 1: Llenar el cuestionario de datos iniciales.
ACTIVIDAD 2: Entrar a su perfil y colocar sus datos.
ACTIVIDAD 3: Conocer a su tutor y a sus compañeros.
ACTIVIDAD 4: Navegar el Curso.
ACTIVIDAD 5: Escriba un ensayo corto para identificar los principales factores que afectan la organización e integración de servicios de salud para el control del VIH y otras enfermedades infecciosas prevalentes en su entorno directo de trabajo en el nivel local.

Fecha límite de realización de estas actividades, incluyendo el envío a su tutor del Ensayo: sábado 9 de abril.

Para ver más detalles sobre estas actividades utilice las flechas que están en la parte inferior de esta pantalla.

Ir a Actividad 1 >>

TODAS LAS ACTIVIDADES INICIALES SON OBLIGATORIAS.

SUBMENÚ TEMA 1

Con el submenú Tema 1 comienzan las 4 semanas de trabajo con los contenidos proporcionados por la OPS.

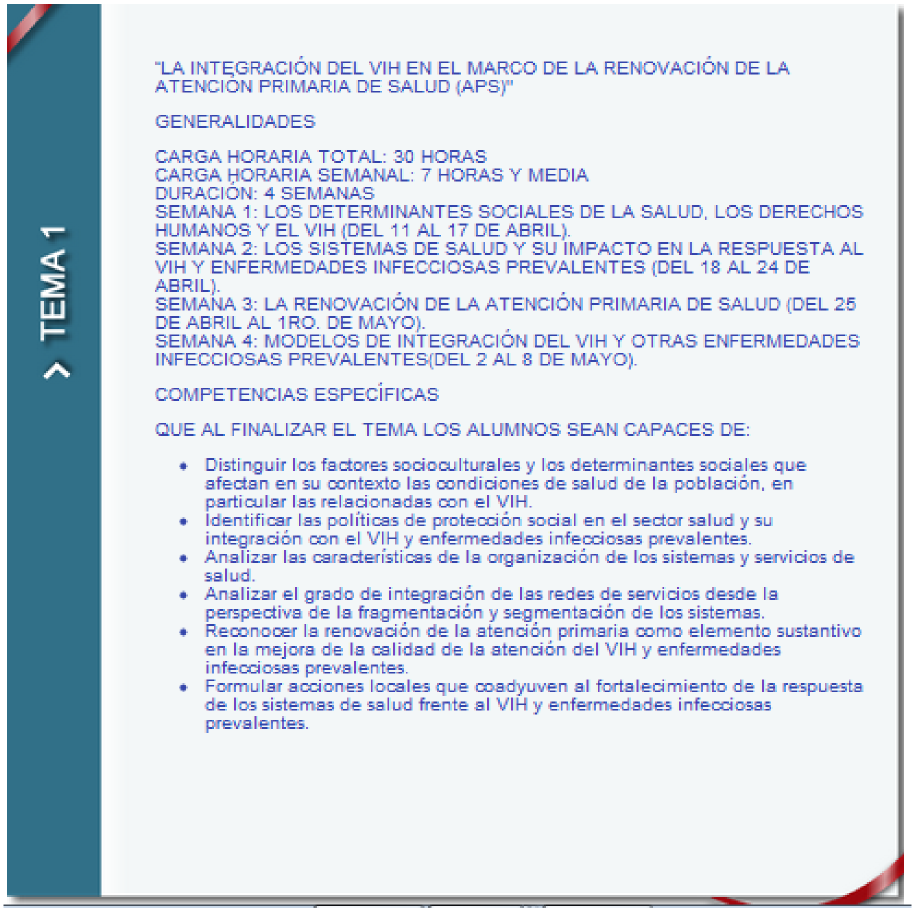

> TEMA 1

"LA INTEGRACIÓN DEL VIH EN EL MARCO DE LA RENOVACIÓN DE LA ATENCIÓN PRIMARIA DE SALUD (APS)"

GENERALIDADES

CARGA HORARIA TOTAL: 30 HORAS
CARGA HORARIA SEMANAL: 7 HORAS Y MEDIA
DURACIÓN: 4 SEMANAS
SEMANA 1: LOS DETERMINANTES SOCIALES DE LA SALUD, LOS DERECHOS HUMANOS Y EL VIH (DEL 11 AL 17 DE ABRIL).
SEMANA 2: LOS SISTEMAS DE SALUD Y SU IMPACTO EN LA RESPUESTA AL VIH Y ENFERMEDADES INFECCIOSAS PREVALENTES (DEL 18 AL 24 DE ABRIL).
SEMANA 3: LA RENOVACIÓN DE LA ATENCIÓN PRIMARIA DE SALUD (DEL 25 DE ABRIL AL 1RO. DE MAYO).
SEMANA 4: MODELOS DE INTEGRACIÓN DEL VIH Y OTRAS ENFERMEDADES INFECCIOSAS PREVALENTES(DEL 2 AL 8 DE MAYO).

COMPETENCIAS ESPECÍFICAS

QUE AL FINALIZAR EL TEMA LOS ALUMNOS SEAN CAPACES DE:

- Distinguir los factores socioculturales y los determinantes sociales que afectan en su contexto las condiciones de salud de la población, en particular las relacionadas con el VIH.
- Identificar las políticas de protección social en el sector salud y su integración con el VIH y enfermedades infecciosas prevalentes.
- Analizar las características de la organización de los sistemas y servicios de salud.
- Analizar el grado de integración de las redes de servicios desde la perspectiva de la fragmentación y segmentación de los sistemas.
- Reconocer la renovación de la atención primaria como elemento sustantivo en la mejora de la calidad de la atención del VIH y enfermedades infecciosas prevalentes.
- Formular acciones locales que coadyuven al fortalecimiento de la respuesta de los sistemas de salud frente al VIH y enfermedades infecciosas prevalentes.

CADA TEMA TIENE UN ESPACIO DE GENERALIDADES, DONDE SE EXPLICAN SUS PRINCIPALES CARACTERÍSTICAS.

Submenú secundario – 1.1 Determinantes sociales. TEMA 1 / SEMANA 1.

Se presentan las actividades que deben ser completadas la primera semana del Tema 1. Nótese que para navegar entre las actividades deben usarse las flechas que se presentan en la parte de debajo de la tabla y que para todas las conferencias hay tres posibilidades: oírlas, descargar la transcripción y/o descargar las diapositivas. En el caso de las lecturas basta un clic sobre el nombre para acceder a ellas. En lo referente a la evaluación se anexa en formato Word la evaluación propuesta y la lista de cotejo. En el espacio de la evaluación el participante tiene acceso directo al enlace para subir sus resultados y entregárselos a su tutor. Esta será la única vía.

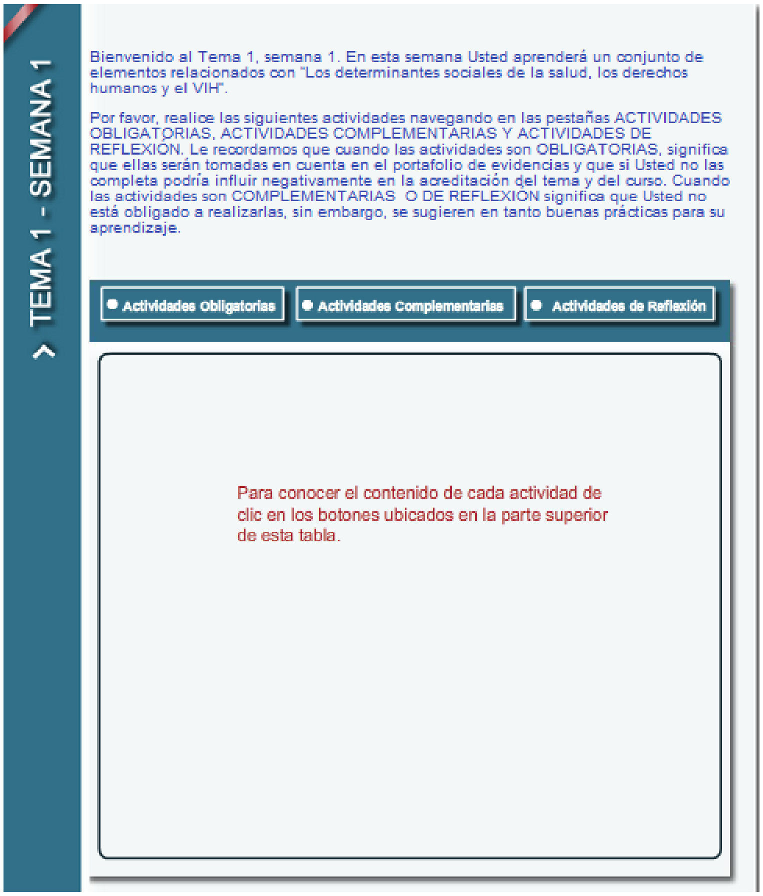

EN CADA CASO SE SEÑALA CUANDO LAS ACTIVIDADES A REALIZAR SON OBLIGATORIAS.

Submenú secundario – 1.2 Sistemas de salud. TEMA 1 / SEMANA 2.

Se presentan las actividades que deben ser completadas la segunda semana del Tema 1. Nótese que para navegar entre las actividades deben usarse las flechas que se presentan en la parte de debajo de la tabla y que para todas las conferencias hay tres posibilidades: oírlas, descargar la transcripción y/o descargar las diapositivas. En el caso de las lecturas basta un clic sobre el nombre para acceder a ellas. En lo referente a la evaluación se anexa en formato Word la evaluación propuesta y la lista de cotejo. En el espacio de la evaluación el participante tiene acceso directo al enlace para subir sus resultados y entregárselos a su tutor. Esta será la única vía.

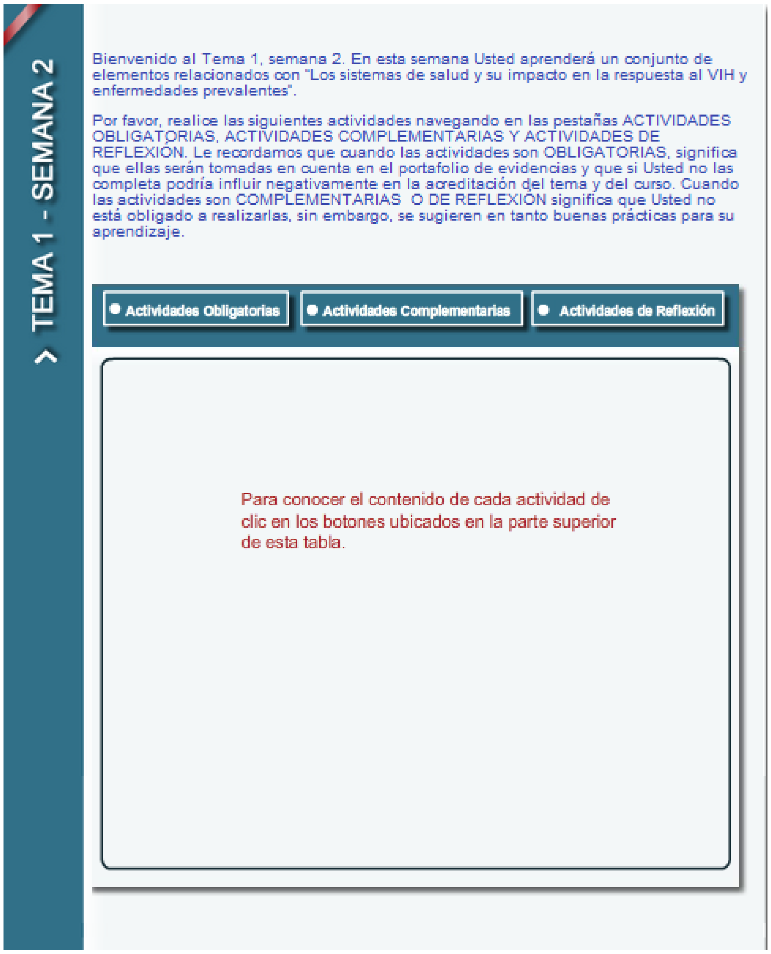

CADA CONFERENCIA PUEDE SER ESCUCHADA O SE PUEDEN DESCARGAR SU TRANSCRIPCIÓN Y SUS DIAPOSITIVAS EN FORMATO PDF.

Submenú secundario – 1.3 Atención Primaria. TEMA 1 / SEMANA 3.

Se presentan las actividades que deben ser completadas la tercera semana del Tema 1. Nótese que para navegar entre las actividades deben usarse las flechas que se presentan en la parte de debajo de la tabla y que para todas las conferencias hay tres posibilidades: oírlas, descargar la transcripción y/o descargar las diapositivas. En el caso de las lecturas basta un clic sobre el nombre para acceder a ellas. En lo referente a la evaluación se anexa en formato Word la evaluación propuesta y la lista de cotejo. En el espacio de la evaluación el participante tiene acceso directo al enlace para subir sus resultados y entregárselos a su tutor. Esta será la única vía.

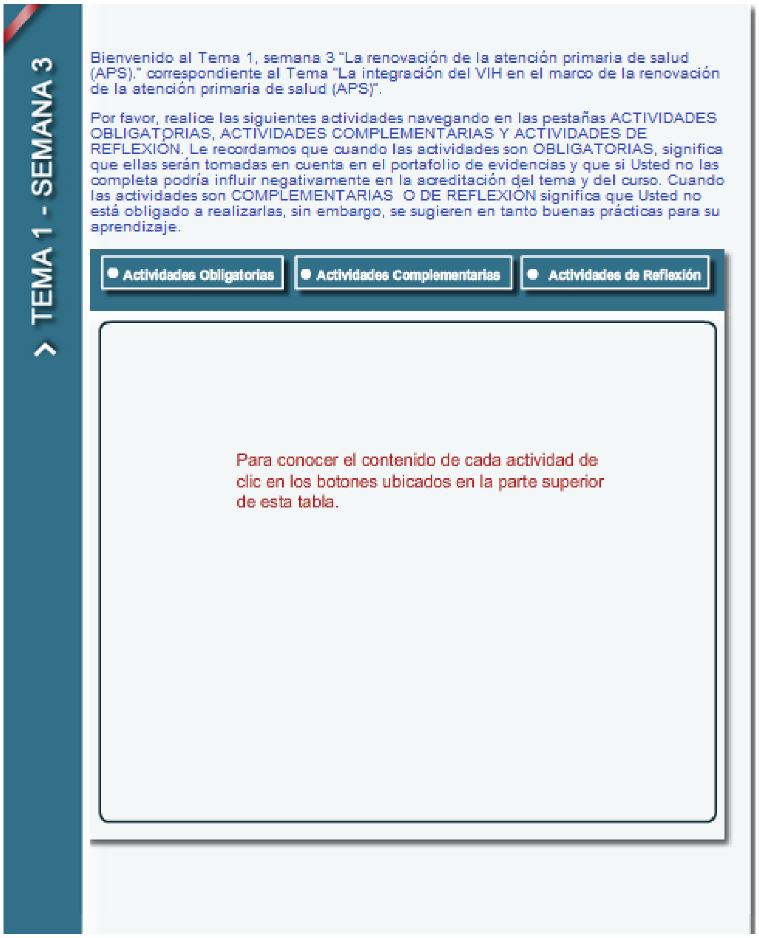

TODAS LAS EVALUACIONES CUALITATIVAS VIENEN ACOMPAÑADAS POR SU LISTA DE COTEJO.

Submenú secundario – 1.4 Modelos de integración. TEMA 1 / SEMANA 4.

Se presentan las actividades que deben ser completadas la cuarta semana del Tema 1. Nótese que para navegar entre las actividades deben usarse las flechas que se presentan en la parte de debajo de la tabla y que para todas las conferencias hay tres posibilidades: oírlas, descargar la transcripción y/o descargar las diapositivas. En el caso de las lecturas basta un clic sobre el nombre para acceder a ellas. En lo referente a la evaluación se anexa en formato Word la evaluación propuesta y la lista de cotejo. En el espacio de la evaluación el participante tiene acceso directo al enlace para subir sus resultados y entregárselos a su tutor. Esta será la única vía.

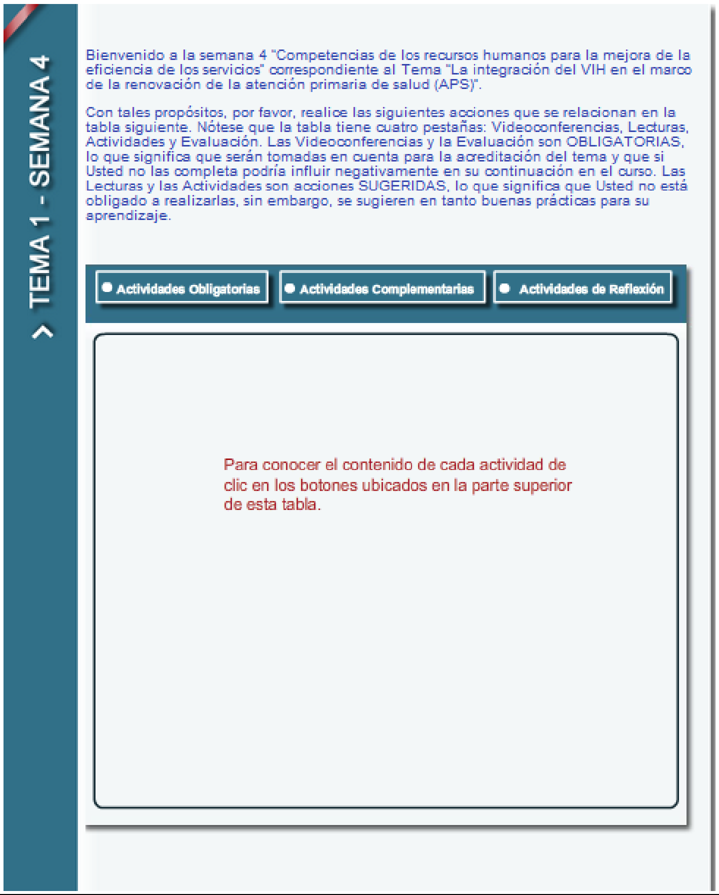

PARA DESCARGAR LAS LECTURAS, BASTA DAR CLIC SOBRE EL NOMBRE DE ELLAS

SUBMENÚ TEMA 2

Con el submenú Tema 2 comienzan las 4 semanas de trabajo con los contenidos proporcionados por la Universidad Johns Hopkins (UJH).

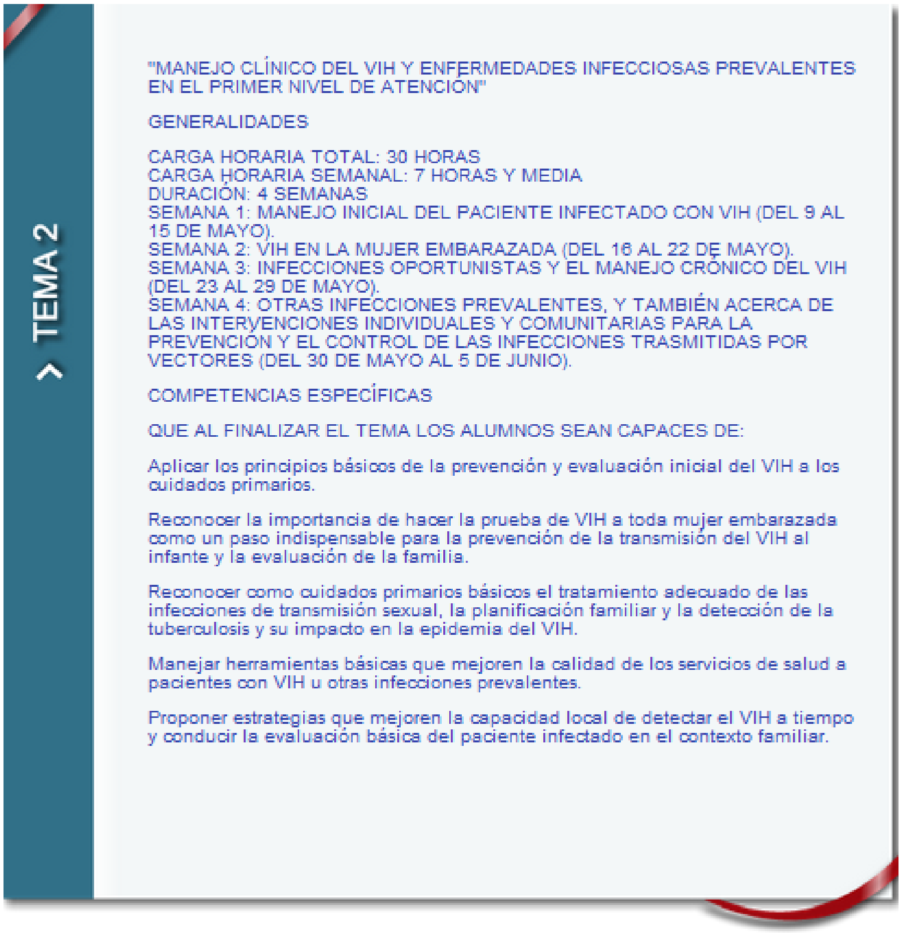

EN TODOS LOS MENÚS DESPLEGADOS, A SU IZQUIERDA, APARECE INDICADO EL TEMA Y LA SEMANA O EL TIPO DE ACTIVIDAD.A QUE SE REFIERE.

Submenú secundario – 2.1 Manejo inicial del VIH. TEMA 2 / SEMANA 1.

Se presentan las actividades que deben ser completadas la primera semana del Tema 2. Nótese que para navegar entre las actividades deben usarse las flechas que se presentan en la parte de debajo de la tabla y que para todas las conferencias hay tres posibilidades: oírlas, descargar la transcripción y/o descargar las diapositivas. En el caso de las lecturas basta un clic sobre el nombre para acceder a ellas. En lo referente a la evaluación la única forma de llenar ambos cuestionarios es en línea.

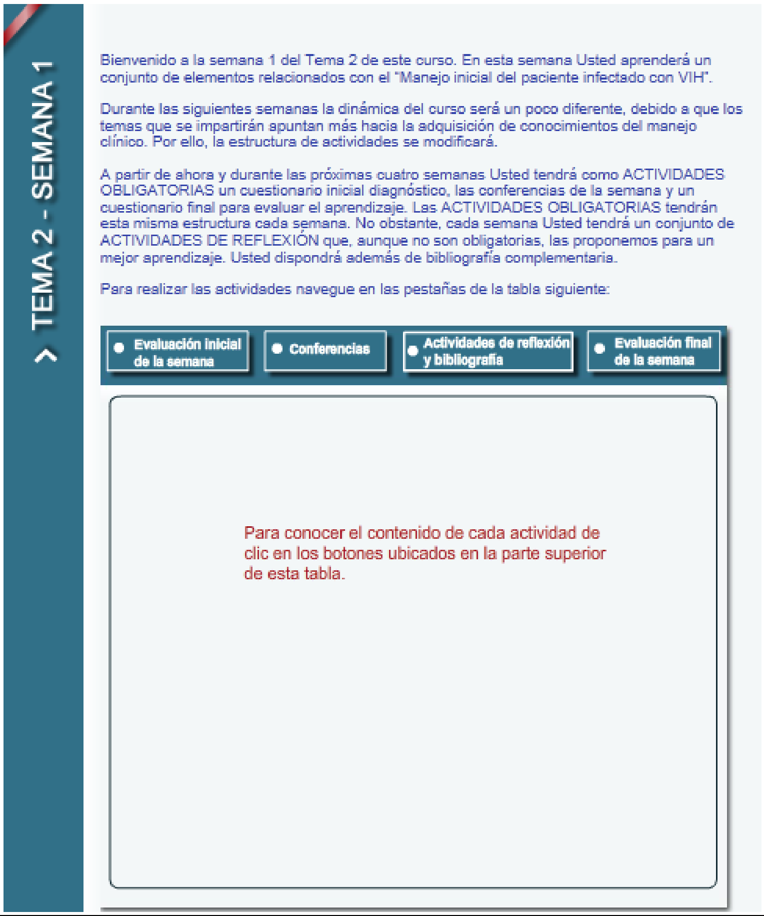

DURANTE EL SEGUNDO TEMA, LAS EVALUACIONES SERÁN A TRAVÉS DE CUESTIONARIOS AUTOMATIZADOS.

NO SE PODRÁ COMPLETAR EL CUESTIONARIO FINAL SIN HABER REALIZADO EL INICIAL.

Submenú secundario – 2.2 – VIH en embarazadas. TEMA 2 / SEMANA 2.

Se presentan las actividades que deben ser completadas la segunda semana del Tema 2. Nótese que para navegar entre las actividades deben usarse las flechas que se presentan en la parte de debajo de la tabla y que para todas las conferencias hay tres posibilidades: oírlas, descargar la transcripción y/o descargar las diapositivas. En el caso de las lecturas basta un clic sobre el nombre para acceder a ellas. En lo referente a la evaluación la única forma de llenar ambos cuestionarios es en línea.

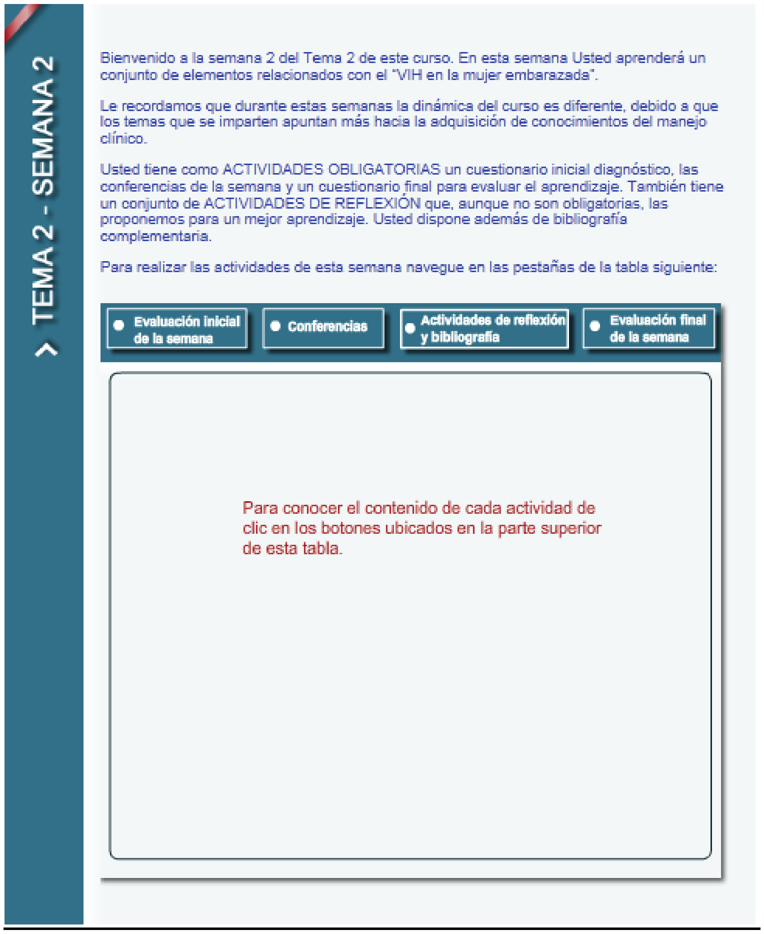

LA ÚNICA FORMA DE REALIZAR LAS EVALUACIONES DEL TEMA 2 ES EN LÍNEA.

Submenú secundario – 2.3 – Manejo crónico del VIH. TEMA 2 / SEMANA 3.

Se presentan las actividades que deben ser completadas la tercera semana del Tema 2. Nótese que para navegar entre las actividades deben usarse las flechas que se presentan en la parte de debajo de la tabla y que para todas las conferencias hay tres posibilidades: oírlas, descargar la transcripción y/o descargar las diapositivas. En el caso de las lecturas basta un clic sobre el nombre para acceder a ellas. En lo referente a la evaluación la única forma de llenar ambos cuestionarios es en línea.

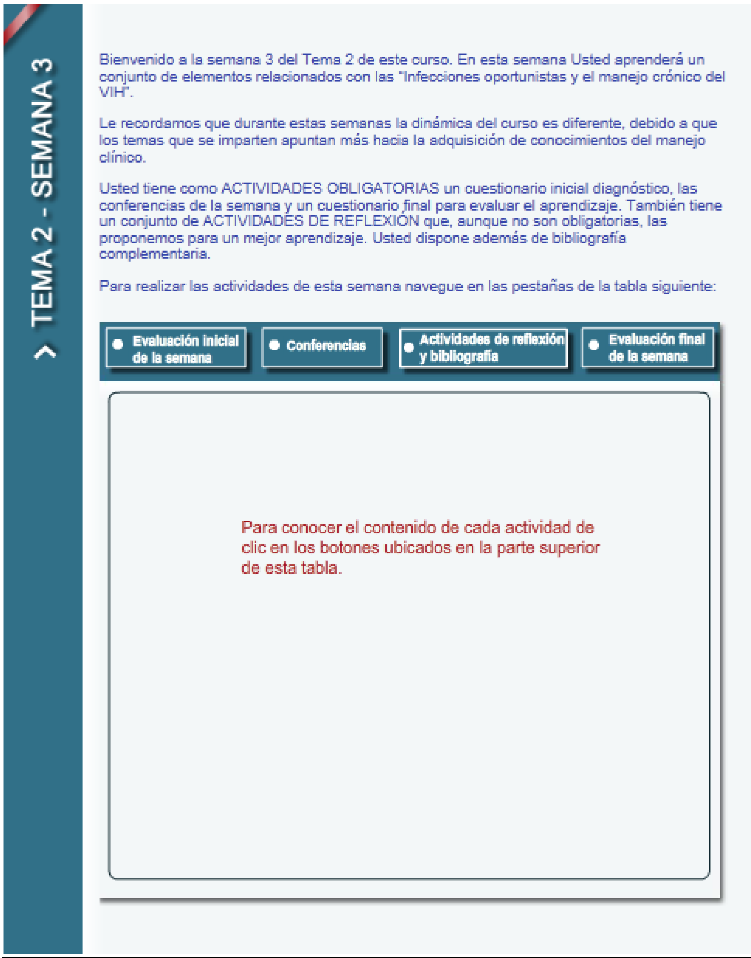

ES IMPORTANTE VISITAR DIARIAMENTE EL FORO Y PARTICIPAR EN LAS REFLEXIONES.

Submenú secundario – 2.4 – Otras infecciones. TEMA 2 / SEMANA 4.

Se presentan las actividades que deben ser completadas la cuarta semana del Tema 2. Nótese que para navegar entre las actividades deben usarse las flechas que se presentan en la parte de debajo de la tabla y que para todas las conferencias hay tres posibilidades: oírlas, descargar la transcripción y/o descargar las diapositivas. En el caso de las lecturas basta un clic sobre el nombre para acceder a ellas. En lo referente a la evaluación la única forma de llenar ambos cuestionarios es en línea.

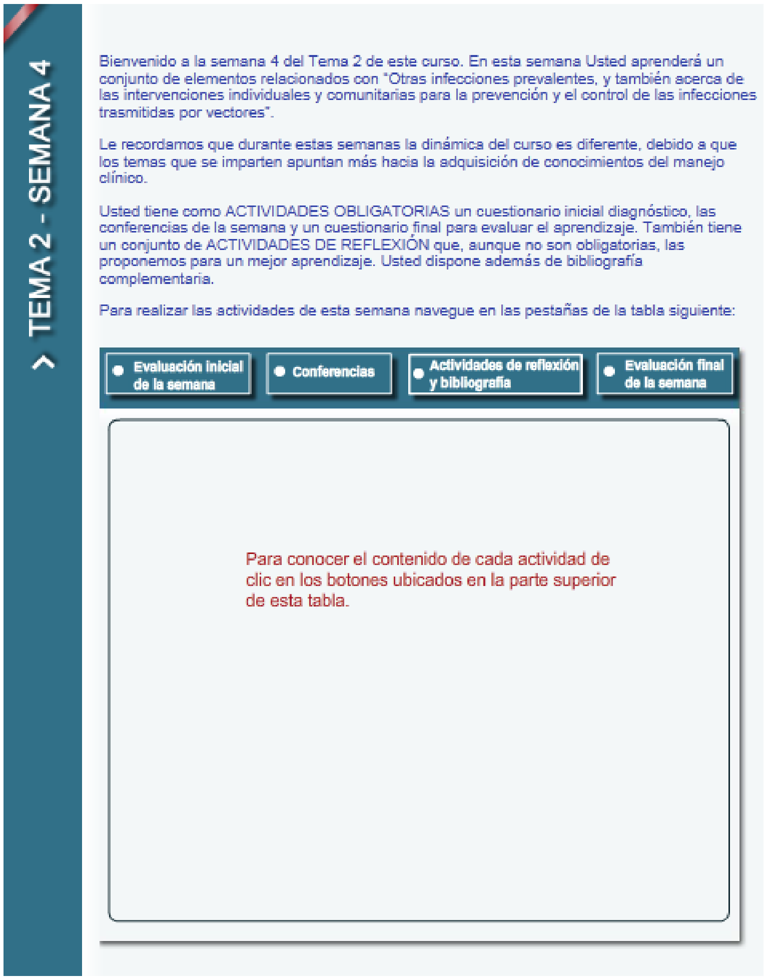

SE ADJUNTAN VALIOSAS BIBLIOGRAFÍAS PRODUCIDAS POR EXPERTOS REGIONALES.

SUBMENÚ ACTIVIDADES FINALES

Con el submenú actividades finales termina la Fase Virtual del curso. Se presentan un conjunto de actividades que deben ser completadas antes de finalizar. Nótese que para navegar entre las actividades deben usarse las flechas que se presentan en la parte de abajo de la tabla.

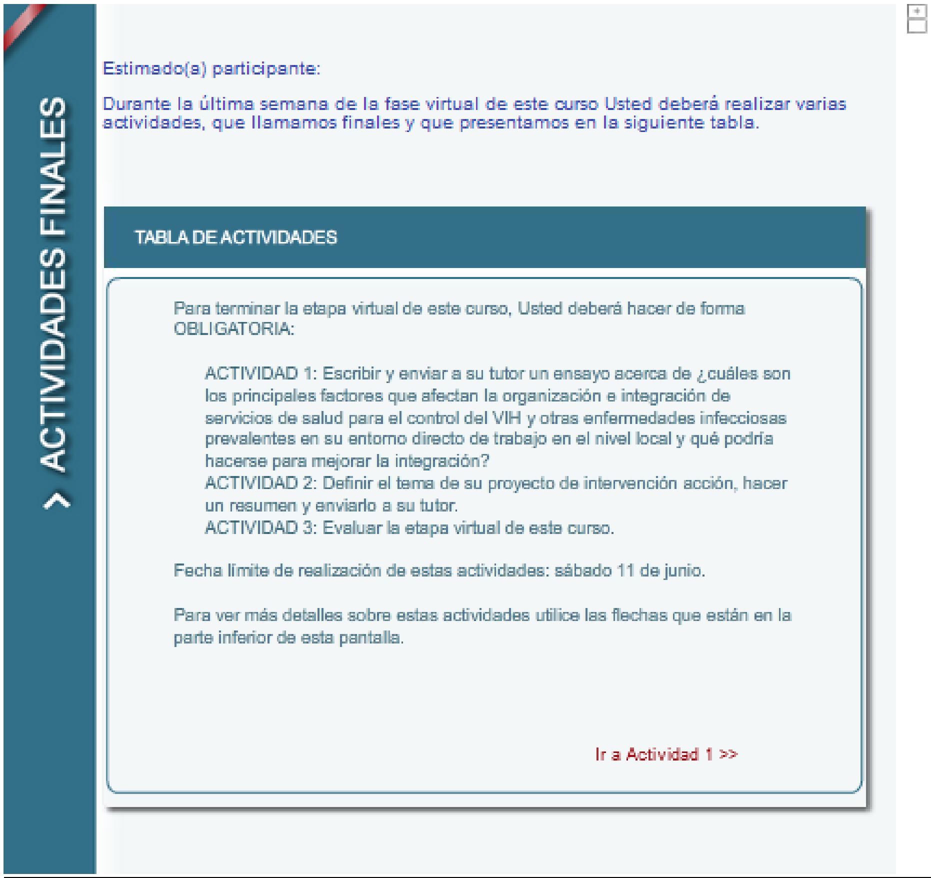

TODAS LAS ACTIVIDADES FINALES SON OBLIGATORIAS.

MENÚ ETAPA PRESENCIAL

En este Menú se despliegan dos submenús que competen a TODA la actividad presencial del curso. El orden lógico de navegación es de arriba hacia abajo.

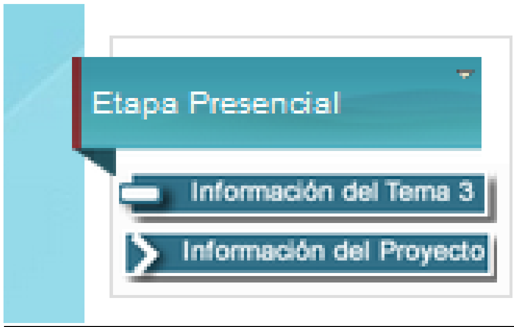

EL MENÚ DE LA ETAPA PRESENCIAL ES SOLO INFORMATIVO.

SUBMENÚ INFORMACIÓN DEL TEMA 3

En el submenú Información del Tema 3 se ofrece información acerca de la parte de este curso que consiste en 40 horas presenciales en el Instituto Conmemorativo Gorgas de Estudios de Salud de Panamá.

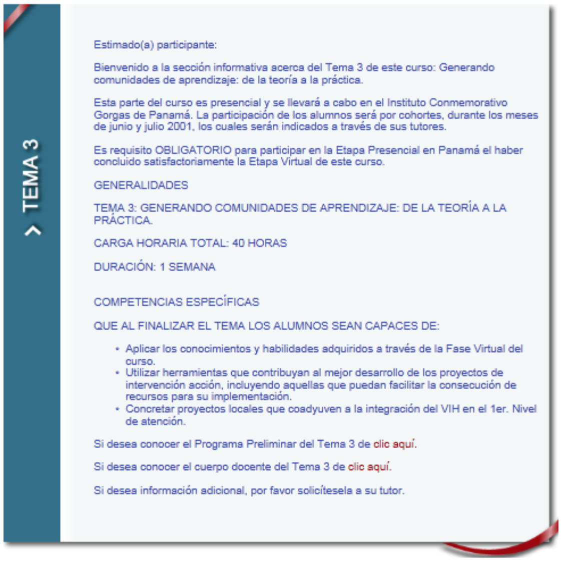

SUBMENÚ INFORMACIÓN DEL PROYECTO

En el submenú Información del Proyecto se ofrecen elementos acerca de cómo elaborar los Proyectos de Intervención acción.

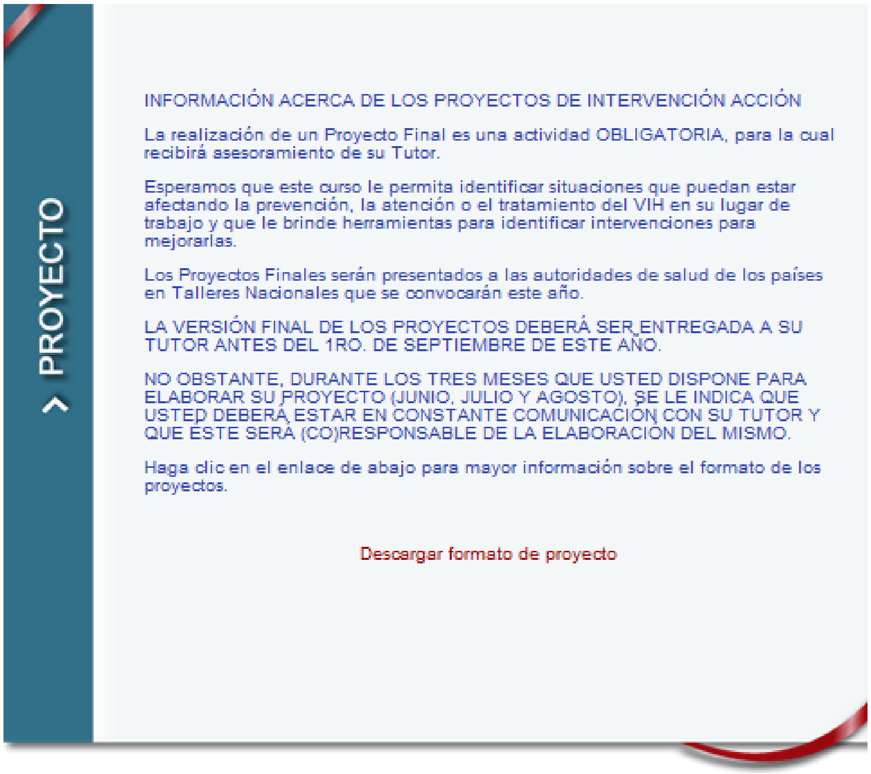

LOS PROYECTOS DE INTERVENCIÓN ACCIÓN DEBEN COMENZAR A GESTARSE DURANTE LA FASE VIRTUAL DEL CURSO.

MENÚ HERRAMIENTAS

En el Menú Herramientas se muestran un conjunto de utilidades a disposición de los participantes, docentes y tutores. Estas son:

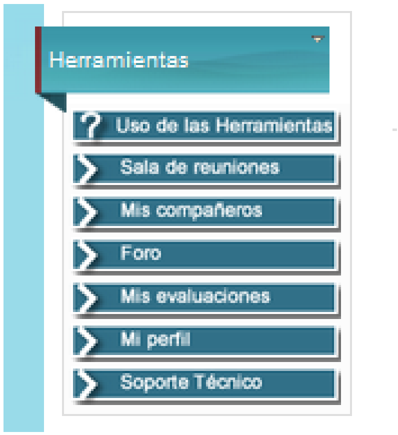

SUBMENÚ USO DE LAS HERRAMIENTAS

En este submenú hay una explicación detallada acerca de para qué sirve cada herramienta propuesta.

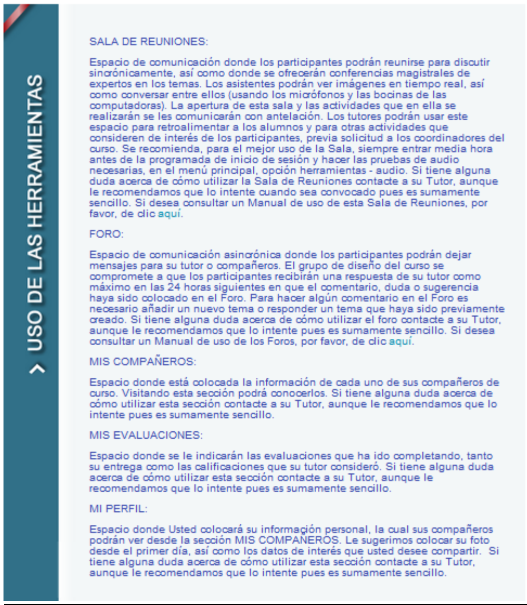

SALA DE REUNIONES:

Espacio de comunicación donde los participantes podrán reunirse para discutir sincrónicamente, así como donde se ofrecerán conferencias magistrales de expertos en los temas. Los asistentes podrán ver imágenes en tiempo real, así como conversar entre ellos (usando los micrófonos y las bocinas de las computadoras). La apertura de esta sala y las actividades que en ella se realizarán se les comunicarán con antelación. Los tutores podrán usar este espacio para retroalimentar a los alumnos y para otras actividades que consideren de interés de los participantes, previa solicitud a los coordinadores del curso. Se recomienda, para el mejor uso de la Sala, siempre entrar media hora antes de la programada de inicio de sesión y hacer las pruebas de audio necesarias, en el menú principal, opción herramientas - audio. Si tiene alguna duda acerca de cómo utilizar la Sala de Reuniones contacte a su Tutor, aunque le recomendamos que lo intente cuando sea convocado pues es sumamente sencillo. Si desea consultar un Manual de uso de esta Sala de Reuniones, por favor, de clic aquí.

FORO:

Espacio de comunicación asincrónica donde los participantes podrán dejar mensajes para su tutor o compañeros. El grupo de diseño del curso se compromete a que los participantes recibirán una respuesta de su tutor como máximo en las 24 horas siguientes en que el comentario, duda o sugerencia haya sido colocado en el Foro. Para hacer algún comentario en el Foro es necesario añadir un nuevo tema o responder un tema que haya sido previamente creado. Si tiene alguna duda acerca de cómo utilizar el foro contacte a su Tutor, aunque le recomendamos que lo intente pues es sumamente sencillo. Si desea consultar un Manual de uso de los Foros, por favor, de clic aquí.

MIS COMPAÑEROS:

Espacio donde está colocada la información de cada uno de sus compañeros de curso. Visitando esta sección podrá conocerlos. Si tiene alguna duda acerca de cómo utilizar esta sección contacte a su Tutor, aunque le recomendamos que lo intente pues es sumamente sencillo.

MIS EVALUACIONES:

Espacio donde se le indicarán las evaluaciones que ha ido completando, tanto su entrega como las calificaciones que su tutor consideró. Si tiene alguna duda acerca de cómo utilizar esta sección contacte a su Tutor, aunque le recomendamos que lo intente pues es sumamente sencillo.

MI PERFIL:

Espacio donde Usted colocará su información personal, la cual sus compañeros podrán ver desde la sección MIS COMPAÑEROS. Le sugerimos colocar su foto desde el primer día, así como los datos de interés que usted desee compartir. Si tiene alguna duda acerca de cómo utilizar esta sección contacte a su Tutor, aunque le recomendamos que lo intente pues es sumamente sencillo.

DURANTE TODA LA FASE VIRTUAL DEL CURSO LOS ALUMNOS Y TUTORES CONTARÁN CON UN CONJUNTO DE HERRAMIENTAS PARA FACILITAR EL PROCESO DE ENSEÑANZA APRENDIZAJE.

SUBMENÚ SALA DE REUNIONES

Este submenú enlaza la Plataforma del curso con otra llamada Elluminate, la cual consiste en un espacio de comunicación sincrónico que ponemos a disposición de tutores y alumnos y que se recomienda se utilice en ediciones posteriores de este curso. Esta Plataforma puede ser utilizada para reuniones, aclaraciones de dudas y otros asuntos de interés entre participantes y tutores, para reforzar algún tema a través de Conferencias Magistrales, para reuniones de grupo, etc.

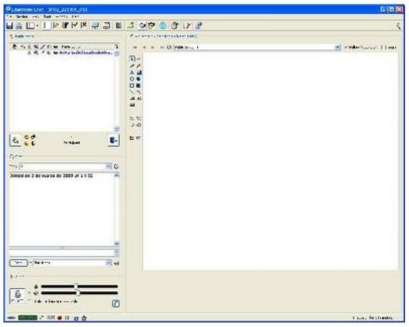

ENTRE LAS HERRAMIENTAS DISPONIBLES HAY UNA SALA DE REUNIONES PARA LA COMUNICACIÓN SINCRÓNICA.

SUBMENÚ MIS COMPAÑEROS

En este submenú los alumnos y tutores podrán acceder al perfil de los restantes participantes del curso.

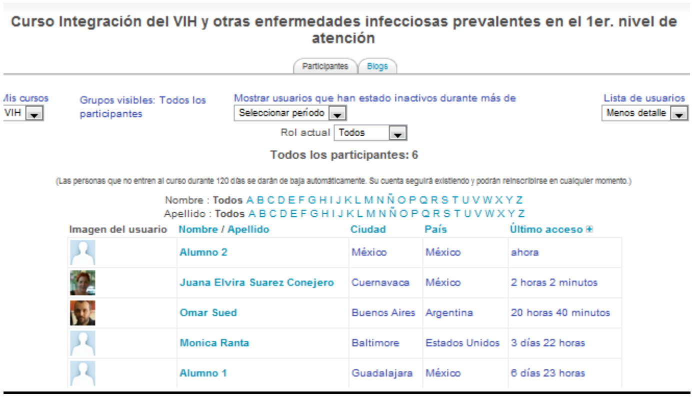

Curso Integración del VIH y otras enfermedades infecciosas prevalentes en el 1er. nivel de atención

Participantes | Blogs

Mis cursos: VIH — Grupos visibles: Todos los participantes — Mostrar usuarios que han estado inactivos durante más de: Seleccionar período — Lista de usuarios: Menos detalle

Rol actual: Todos

Todos los participantes: 6

(Las personas que no entren al curso durante 120 días se darán de baja automáticamente. Su cuenta seguirá existiendo y podrán reinscribirse en cualquier momento.)

Nombre : **Todos** A B C D E F G H I J K L M N Ñ O P Q R S T U V W X Y Z
Apellido : **Todos** A B C D E F G H I J K L M N Ñ O P Q R S T U V W X Y Z

Imagen del usuario	Nombre / Apellido	Ciudad	País	Último acceso
	Alumno 2	México	México	ahora
	Juana Elvira Suarez Conejero	Cuernavaca	México	2 horas 2 minutos
	Omar Sued	Buenos Aires	Argentina	20 horas 40 minutos
	Monica Ranta	Baltimore	Estados Unidos	3 días 22 horas
	Alumno 1	Guadalajara	México	6 días 23 horas

SUBMENÚ FORO

A través de este Submenú alumnos, tutores y docentes podrán tener comunicación asincrónica y reforzar las actividades de reflexión de la parte virtual del curso.

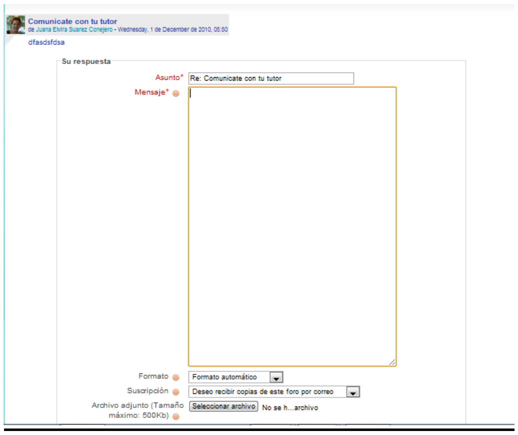

SUBMENÚ MIS EVALUACIONES

En este espacio los alumnos podrán revisar si sus evaluaciones aparecen como entregadas y cuáles fueron las calificaciones obtenidas.

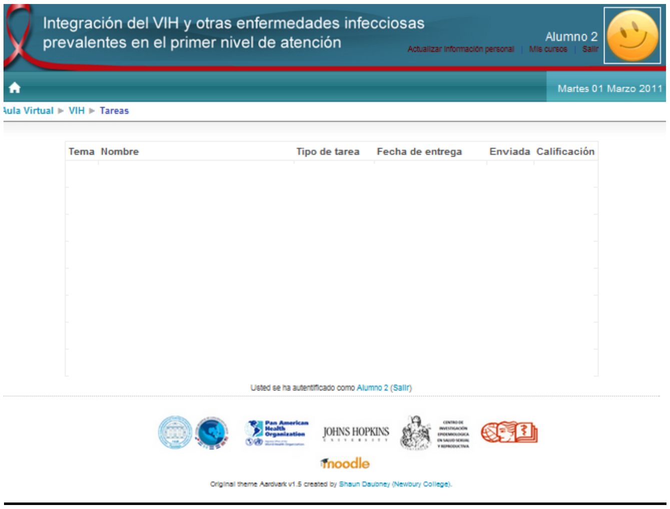

ENTRE LAS HERRAMIENTAS DISPONIBLES HAY UN FORO PARA LA COMUNICACIÓN ASINCRÓNICA.

SUBMENÚ MI PERFIL

En este espacio los participantes podrán colocar sus datos personales y laborales, subir su foto, cambiar su información, administrar su contraseña del curso y disponen de un blog.

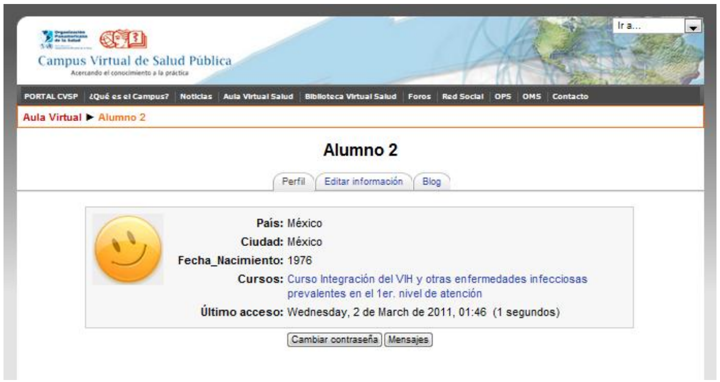

SUBMENÚ SOPORTE TÉCNICO

En este menú se encuentra la información acerca de qué hacer en caso de que un participante necesite soporte técnico de la Plataforma.

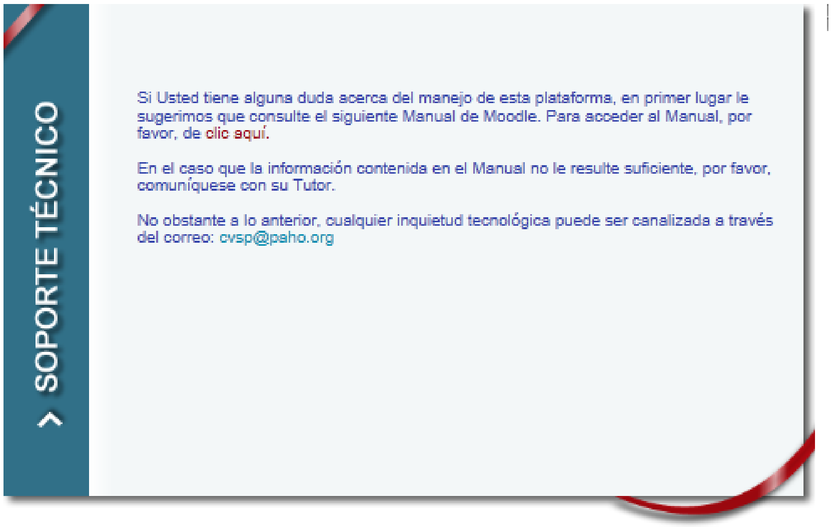

TODO EL TIEMPO ESTARÁ DISPONIBLE EL SOPORTE TÉCNICO PARA ALUMNOS Y TUTORES.

MENÚ ADMINISTRACIÓN

Finalmente está el Menú administración. Este solo será visible para los tutores. Desde él, los tutores podrán calificar las evidencias de aprendizaje de sus alumnos, a través del SUBMENÚ Calificador.

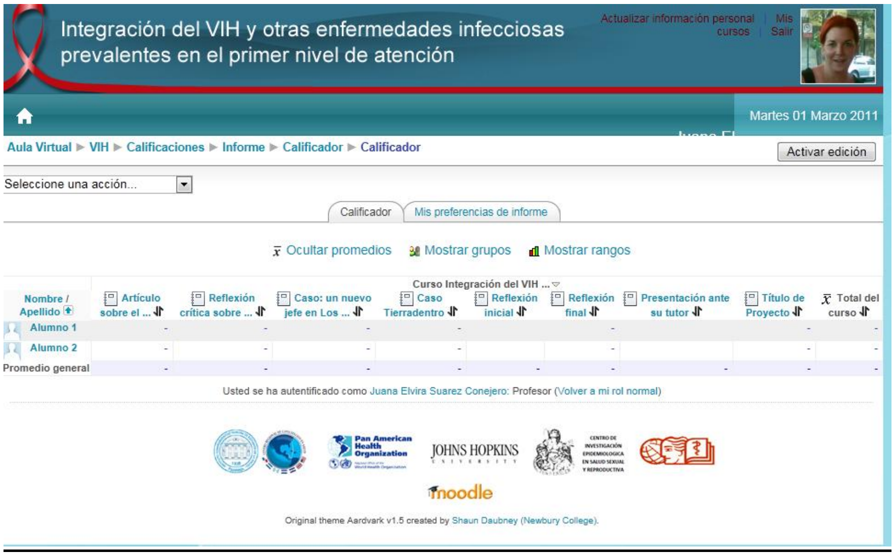

EL TUTOR DEBE COLOCAR EN LA PLATAFORMA LAS CALIFICACIONES DE SUS ALUMNOS Y SEGUIR TODO EL PROCESO EVALUATIVO.

CAPÍTULO V. EL ROL DEL TUTOR EN EL CURSO.

5.1 EL ROL DEL TUTOR DURANTE LA FASE VIRTUAL.

Como comentamos anteriormente, la fase virtual de este curso está programada para 10 semanas, bajo el supuesto de que los alumnos se inscribieron previamente en la Plataforma Moodle. El Campus Virtual de Salud Pública de la OPS brinda un programa de familiarización, enlazado a la plataforma, que estará disponible durante el proceso de inscripciones.

Como hemos dicho anteriormente, a partir de inicio del curso virtual:

- La semana 1 está prevista para actividades iniciales.
- Las semanas 2, 3, 4 y 5 concentran el Tema 1, impartido por la OPS.
- Las semanas 6, 7, 8 y 9 concentran el Tema 2, impartido por la Universidad Johns Hopkins.
- La semana 10 está prevista para las actividades finales.

Durante las 10 semanas virtuales de este curso, el Tutor tendrá las siguientes funciones:

- Animación: El tutor debe contactar a los estudiantes, sobre todo a los que no tengan participación e instarlos a participar en el foro y a realizar todas las actividades.

EL TUTOR ES ANTE TODO UN ANIMADOR.

DEBE CONTACTAR A LOS ESTUDIANTES, SOBRE TODO A LOS QUE NO TENGAN PARTICIPACIÓN, E INSTARLOS A REALIZAR TODAS LAS ACTIVIDADES.

- Aclaración de dudas: El tutor debe apoyar a los estudiantes aclarándoles dudas tanto de contenidos como tecnológicas. Se anexa a este manual un listado de expertos a contactar para el caso de dudas que superen a los tutores.

- Seguimiento: El tutor debe darle a cada alumno un estricto seguimiento con dos propósitos. Primero, para que mantenga el ritmo de trabajo y termine las actividades cada semana. Segundo, para garantizar que acredite el curso y adquiera las competencias esperadas. El tutor deberá responder las dudas en las siguientes 24 horas después de que fueron planteadas por el participante.

- Evaluación: El tutor será el encargado de evaluar las evidencias de aprendizaje cualitativas. Para ello dispondrá de listas de cotejo que le permitirán utilizar criterios estándares y que serán de conocimiento de los estudiantes. Cada semana que corresponda el tutor deberá revisar las evidencias de aprendizaje y comunicar a los alumnos, junto con la lista de cotejo debidamente completada, los comentarios en las siguientes 72 horas después de haber sido entregado el trabajo por parte del participante. Se le recuerda a los tutores que este modelo pedagógico permite que el estudiante tenga varias oportunidades, en el caso de las evidencias cualitativas, de revisar su trabajo, por lo que la nota final corresponderá con la versión final entregada. El tutor deberá colocar en la plataforma Moodle la calificación correspondiente.

EL TUTOR DEBERÁ RESPONDER LAS DUDAS EN LAS SIGUIENTES 24 HORAS DESPUÉS QUE HAYAN SIDO PLANTEADAS.

EL TUTOR DEBERÁ CALIFICAR LAS EVALUACIONES EN LAS SIGUIENTES 72 HORAS DESPUÉS DE SU ENTREGA POR PARTE DEL ALUMNO.

5.2 EL CALENDARIO DEL TUTOR DURANTE LA FASE VIRTUAL.

ACTIVIDADES INICIALES - SEMANA 1	
LO QUE DEBE HACER EL TUTOR	DÍA / TIEMPO DE RESPUESTA
Aclarar todas las dudas que los alumnos tengan. Para ello deberá entrar diariamente a la plataforma y explorar el Foro. También deberá revisar su correo electrónico, incluyendo la bandeja de no deseados.	Todos los días
Verificar que todos sus alumnos hayan llenado el cuestionario de datos iniciales.	Jueves
Contactar a quienes no lo hayan hecho y solicitarles que lo hagan. Darle seguimiento.	De jueves a sábado
Verificar que todos sus alumnos hayan entrado a su perfil y colocado sus datos.	Jueves
Contactar a quienes no lo hayan hecho y solicitarles que lo hagan. Darle seguimiento.	De jueves a sábado
Verificar que todos sus alumnos le hayan escrito un correo presentándose.	Jueves
Responder amablemente los correos a todos los alumnos y presentarse con ellos.	En las 24 horas siguientes a que el alumno envió el correo
Enviar un correo a todos sus alumnos recordando que el sábado es la fecha de entrega de la REFLEXIÓN INICIAL (evidencia de aprendizaje) y ofreciéndose para aclarar dudas. Recordarles que la única forma de entregar esta evidencia es a través de la plataforma del curso.	Jueves
Verificar que todos sus alumnos hayan enviado la evidencia de aprendizaje a través de la plataforma.	Sábado
Contactar a quienes no lo hayan hecho y solicitarles que lo hagan. Darle seguimiento.	Sábado y domingo
Calificar, según la lista de cotejo, las evidencias de aprendizaje.	Sábado y domingo
Enviar un correo a cada alumno con la retroalimentación de la evidencia de aprendizaje.	Sábado y domingo
Subir a plataforma las calificaciones de los alumnos.	Domingo

EL TUTOR DEBERÁ RESPONDER AMABLEMENTE TODOS LOS CORREOS DE TODOS LOS ALUMNOS.

TEMA 1 - LA INTEGRACIÓN DEL VIH EN EL MARCO DE LA RENOVACIÓN DE LA ATENCIÓN PRIMARIA DE SALUD (APS). SEMANAS 2 A LA 5.	
LO QUE DEBE HACER EL TUTOR	DÍA / TIEMPO DE RESPUESTA
Aclarar todas las dudas que los alumnos tengan. Para ello deberá entrar diariamente a la plataforma y explorar el Foro. También deberá revisar su correo electrónico, incluyendo la bandeja de no deseados.	Todos los días
Animar el foro. Instar a los alumnos a participar en el caso que no lo hayan hecho. Utilizar como motivación las preguntas de reflexión de la semana.	Todos los días
Verificar que todos sus alumnos hayan realizado las actividades obligatorias. Para ello, puede enviarles un correo preguntando si han podido completar las actividades de la semana o si necesitan algún apoyo. Darle seguimiento a los casos que corresponda.	Todos los jueves
Responder amablemente los correos que reciba de los alumnos.	En las 24 horas siguientes a que el alumno envió el correo
Enviar un correo a todos sus alumnos recordando que el sábado es la fecha de entrega de la evidencia de aprendizaje y ofreciéndose para aclarar dudas. Recordarles que la única forma de entregar esta evidencia es a través de la plataforma del curso.	Todos los jueves
Verificar que todos sus alumnos hayan enviado la evidencia de aprendizaje a través de la plataforma.	Todos los sábados
Contactar a quienes no lo hayan hecho y solicitarles que lo hagan. Darle seguimiento.	Todos los sábados y domingos
Calificar, según las listas de cotejo, las evidencias de aprendizaje.	Todos los sábados y domingos
Enviar un correo a cada alumno con la retroalimentación de la evidencia de aprendizaje.	Todos los sábados y domingos
Subir a plataforma las calificaciones de los alumnos.	Todos los domingos

EL TUTOR VELAR PORQUE TODOS LOS ALUMNOS CONSERVEN EL RITMO SEMANAL DEL CURSO.

TEMA 2 – MANEJO CLÍNICO DEL VIH. SEMANAS 6 A LA 9.	
LO QUE DEBE HACER EL TUTOR	DÍA / TIEMPO DE RESPUESTA
Aclarar todas las dudas que los alumnos tengan. Para ello deberá entrar diariamente a la plataforma y explorar el Foro. También deberá revisar su correo electrónico, incluyendo la bandeja de no deseados.	Todos los días
Animar el foro. Instar a los alumnos a participar en el caso que no lo hayan hecho. Utilizar como motivación el tema de la semana.	Todos los días
Verificar que todos sus alumnos hayan completado la evaluación inicial en plataforma.	Todos los martes
Contactar a quienes no lo hayan hecho y solicitarles que lo hagan. Darle seguimiento.	Todos los martes
Verificar que todos sus alumnos hayan visto las conferencias. Para ello, puede enviarles un correo preguntando si han podido ver todas las conferencias o si necesitan algún apoyo. Darle seguimiento a los casos que corresponda.	Todos los viernes
Responder amablemente los correos que reciba de los alumnos.	En las 24 horas siguientes a que el alumno envió el correo
Enviar un correo a todos sus alumnos recordando que el sábado es la fecha en que deben haber culminado las actividades y respondido la evaluación final de la semana. Ofrecerse para aclarar dudas.	Todos los jueves
Verificar que todos sus alumnos hayan completado la evaluación final en plataforma.	Todos los sábados
Contactar a quienes no lo hayan hecho y solicitarles que lo hagan. Se recuerda que en el caso del Tema 2 se permite hasta el miércoles de la semana siguiente completar este cuestionario, Darle seguimiento.	Todos los sábados y domingos
Verificar que están correctamente colocadas en plataforma las calificaciones de los alumnos correspondientes a la semana.	Todos los domingos

EL TUTOR DEBERÁ ACLARAR EFICIENTEMENTE TODAS LAS DUDAS O CANALIZARLAS DE INMEDIATO.

ACTIVIDADES FINALES - SEMANA 10	
LO QUE DEBE HACER EL TUTOR	DÍA / TIEMPO DE RESPUESTA
Aclarar todas las dudas que los alumnos tengan. Para ello deberá entrar diariamente a la plataforma y explorar el Foro. También deberá revisar su correo electrónico, incluyendo la bandeja de no deseados.	Todos los días
Enviar un correo a todos sus alumnos recordando que el sábado es la fecha de entrega de todas las actividades de esta semana (REFLEXIÓN FINAL, RESUMEN DEL PROYECTO Y CUESTIONARIO DE EVALUACIÓN DEL CURSO) y ofreciéndose para aclarar dudas. Recordarles que la única forma de entregar esta evidencia es a través de la plataforma del curso.	Jueves
Verificar que todos sus alumnos hayan enviado la REFLEXIÓN FINAL Y EL RESUMEN DEL PROYECTO a través de la plataforma.	Sábado
Contactar a quienes no lo hayan hecho y solicitarles que lo hagan. Darle seguimiento.	Sábado y domingo
Calificar, según la lista de cotejo, las dos evidencias de aprendizaje.	Sábado y domingo
Enviar un correo a cada alumno con la retroalimentación de la evidencia de aprendizaje.	Sábado y domingo
Subir a plataforma las calificaciones de los alumnos.	Domingo
Verificar que todos sus alumnos completen el cuestionario de evaluación del curso a través de la plataforma.	Sábado
Contactar a quienes no lo hayan hecho y solicitarles que lo hagan. Darle seguimiento.	Sábado y domingo
Enviar un correo electrónico a los alumnos con la calificación final de toda la fase online según la compilación de la plataforma.	Domingo

EL TUTOR DEBERÁ RETROALIMENTAR LAS EVIDENCIAS DE APRENDIZAJE CUALITATIVAS.

5.3 EL ROL DEL TUTOR DURANTE LA FASE PRESENCIAL DEL CURSO.

En la segunda Fase de este curso, la Presencial, un primer momento será la participación de los alumnos que hayan completado la Fase Virtual en un intensivo de 40 horas que se ofrecerá en el Instituto Conmemorativo Gorgas de Estudios de Salud de Panamá durante una semana.

Los tutores participarán en este intensivo presencial y su rol principal será monitorear el trabajo de los alumnos durante toda la semana y evaluarlos. El instrumento de evaluación de estas 40 horas será un Diario de Aprendizaje, donde los alumnos describirán los conocimientos adquiridos cada día. Además, los tutores durante esa semana deberán trabajar con los alumnos en la pregunta punto de partida de los Proyectos de Intervención Acción, las cuales se discutirán el último día en un Taller de metodología de la investigación.

LOS TUTORES PARTICIPARÁN EN LA FASE PRESENCIAL DE ESTE CURSO EN EL INSTITUTO GORGAS PARA EVALUAR A SUS ALUMNOS.

5.4 EL ROL DEL TUTOR EN LOS PROYECTOS DE INTERVENCIÓN ACCIÓN. LOS TALLERES NACIONALES.

El tutor será el Mentor de los Proyectos de Intervención- Acción, en tanto co-partícipe y colaborador de los proyectos de sus alumnos.

Los tutores deberán:

1. Ser co-responsables de la elaboración de los proyectos, de su calidad y factibilidad.
2. Velar porque sean entregados a tiempo, tanto los resultados finales como los parciales.
3. Apoyar a los alumnos en sus presentaciones en los Talleres nacionales antes las autoridades de salud del país.

Para facilitar este rol, es importante señalar que los mejores mentores son aquellos entusiastas e inspiradores. Para poder inspirar a otros tienen que emanar confianza, confiabilidad y credibilidad.

Entre las cualidades principales de un buen mentor están el ser un buen oyente, persistente, comprometido y paciente. Sin embargo, el mentor es un agente de recursos y no un proveedor de recursos. Es decir, no proporciona los recursos, sino que indica dónde y cómo acceder a éstos.

Podemos señalar las siguientes características de un mentor "ideal":

- Modelo a seguir: los alumnos quieren imitar sus actitudes, lo admiran y quieren ser como él (ella).
- Excelente maestro: ayuda a los alumnos a adquirir nuevos conocimientos, informaciones o destrezas.
- Buen compañero: comparte con sus alumnos intereses y experiencias.
- Firme apoyo: apoya los esfuerzos de sus estudiantes, los motiva y ayuda.

EL TUTOR SERÁ EL MENTOR DE LOS PROYECTOS DE INTERVENCIÓN ACCIÓN.

POR ELLO, DEBE SER UN BUEN OYENTE, PERSISTENTE, COMPROMETIDO Y PACIENTE.

DEBE SER TAMBIIÉN UN AGENTE DE RECURSOS.

Pan American Health Organization

- Proveedor de recursos: pone en contacto a sus alumnos con personas, ideas o lugares que le provean oportunidades de desarrollo.

FORMA DE TRABAJO EN LA ETAPA DE PROYECTOS

Los alumnos serán enteramente responsables de realizar los proyectos de intervención acción. Si un alumno no culmina el Proyecto no podrá acreditar el curso.

Los tutores serán corresponsables de los proyectos, en tal sentido, están en la obligación de apoyar metodológicamente a los alumnos para su buen desarrollo.

Como herramientas de trabajo se ponen a disposición Salas de Elluminate, cuyo horario de uso debe ser comunicado por parte de los tutores a los alumnos. Se consideran OBLIGATORIAS al menos tres sesiones de Elluminate entre tutores y alumnos antes del 1ro. de septiembre. Los tutores registrarán la asistencia de los alumnos y lo tomarán en cuenta para su calificación final.

Otra herramienta de trabajo será el correo electrónico y se usará principalmente para la aclaración de dudas, corrección de proyectos y apoyo directo al estudiante. Los tutores tienen el compromiso de responder a los alumnos en 24 horas como máximo. Los alumnos adquieren el compromiso de responder los correos de los tutores en 72 horas como máximo. Los tutores registrarán las respuestas tardías de correo y lo tomarán en cuenta en la calificación final de los alumnos.

PRODUCTOS PARCIALES Y FINALES QUE DEBEN ENTREGAR LOS ALUMNOS

Hay que entregar 2 productos parciales y 3 productos finales. Ellos son:

PRODUCTOS PARCIALES:

- 1ra. Propuesta de proyecto
  Fecha de entrega: 1ro de agosto
  Tipo de actividad: OBLIGATORIA
  Características: 2 páginas en Word como máximo, letra Arial 12, a espacio y medio.
  Forma de entrega: Por correo electrónico a su tutor en versión Word.
  Puntaje: No acumula puntos.
  Debe contener:
  1. Título.
  2. Objetivos.

- 2da. Propuesta de proyecto
  Fecha de entrega: 15 de agosto
  Tipo de actividad: OBLIGATORIA
  Características: 10 páginas en Word como máximo, letra Arial 12, a espacio y medio.
  Forma de entrega: Por correo electrónico a su tutor en versión Word.
  Puntaje: No acumula puntos.
  Debe contener:
  1. Título.
  2. Objetivos.

3. Descripción ampliada de la propuesta (exploración en el entorno y exploración bibliográfica).
4. Resultados esperados.

PRODUCTOS FINALES:

- Propuesta final de proyecto
  Fecha de entrega: 1ro de septiembre
  Tipo de actividad: OBLIGATORIA
  Características: 20 páginas en Word como máximo, letra Arial 12, a espacio y medio.
  Forma de entrega: Por correo electrónico a su tutor en versión Word.
  Debe contener:
  1. Título.
  2. Objetivos.
  3. Descripción ampliada de la propuesta (exploración en el entorno y exploración bibliográfica).
  4. Resultados esperados.
  5. Diseño metodológico para medir los resultados esperados.
  6. Cronograma
  7. Presupuesto.

- Resumen (Abstract) del proyecto.
  Fecha de entrega: 1ro de septiembre
  Tipo de actividad: OBLIGATORIA
  Características: 1 página en Word como máximo, letra Arial 12, a espacio y medio.
  Forma de entrega: Por correo electrónico a su tutor en versión Word.

- Presentación en Power Point del proyecto para su defensa en los Talleres nacionales.
  Fecha de entrega: 1ro de septiembre
  Tipo de actividad: OBLIGATORIA
  Características: 5 láminas de Power Point como máximo.
  Forma de entrega: Por correo electrónico a su tutor en versión Power Point.

EVALUACIÓN DE LOS PROYECTOS DE INTERVENCIÓN ACCIÓN

La fase de proyectos tiene un valor total de 35 puntos.

Actividad	Puntaje
Participación sesiones Elluminate	3 puntos (1 punto por cada sesión)
Respuesta a tiempo de los correos electrónicos de su tutor	1 punto
Entrega en fecha de la 1ra. Propuesta de proyecto	1 punto
Entrega en fecha de la 2da. Propuesta de proyecto	1 punto
Entrega en fecha de la versión final del Proyecto	2 puntos
Entrega en fecha del Resumen (abstract) del Proyecto	2 puntos
Contenido del proyecto (según rúbrica que se adjunta)	14 puntos
Contenido del resumen del proyecto (según rúbrica que se adjunta)	6 puntos
Defensa del proyecto	5 puntos
Total	35 puntos

5.5 LA MEDICIÓN DEL DESEMPEÑO DE LOS TUTORES

Durante todo el curso los tutores serán evaluados por la Coordinación Académica. En la Fase virtual los alumnos deberán contestar un cuestionario donde algunas preguntas buscan directamente conocer el desempeño de los tutores y la imagen que tienen los alumnos acerca de ellos. En la fase presencial se realizarán Focus Group al respecto.

LOS TUTORES SERÁN EVALUADOS POR SUS ALUMNOS Y POR EL GRUPO DE DESARROLLO CURRICULAR.

Se apreciará en los tutores, entre otras características:

1. Cortesía y expresión adecuada.
2. Excelente ortografía y dominio de la gramática española.
3. Rapidez en contestar en los foros, sala de reuniones, correos electrónicos, etc. Se recuerda el compromiso de las respuestas a foros y a correos en menos de 24 horas.
4. Flexibilidad y seriedad en el proceso evaluativo. Apego a las listas de cotejo.
5. Puntualidad y asistencia a todas las actividades.
6. Cumplimiento estricto de los calendarios propuestos.
7. Buenas relaciones humanas con los participantes del curso y otros tutores.
8. Adhesión a las normas que este Manual expone.

CAPÍTULO VI. EL FUTURO: IMPLEMENTAR ACCIONES Y CREAR COMUNIDADES DEL CONOCIMIENTO.

6.1 LAS LICENCIAS CREATIVE COMMONS. EL ACCESO LIBRE A LA INFORMACIÓN

El equipo de Desarrollo Curricular, la Coordinación Académica de este curso y su financista, como promotores del libre conocimiento y su acceso para todos, ponen este curso a disposición de los interesados en replicar ediciones posteriores.

Para ello, el curso se oferta liberado bajo una Licencia

creative commons

¿Qué son las Licencias Creative Commons?

Las licencias Creative Commons o CC están inspiradas en la licencia GPL (General Public License). La idea principal es posibilitar un modelo legal ayudado por herramientas informáticas para facilitar la distribución y el uso de contenidos.

Existen diversas licencias Creative Commons. Cada una de ellas tiene diferentes configuraciones o principios, como el derecho del autor original a dar libertad para citar su obra, reproducirla, crear obras derivadas, ofrecerlas públicamente, etc.

LA MEJOR FORMA DE PROMOVER EL CONOCIMIENTO ES LIBERARLO Y HACERLO ACCESIBLE A TODOS.

También tienen diferentes restricciones, como no permitir el uso comercial o respetar la autoría original. Para más información, visitar el sitio http://creativecommons.org/

¿Qué significa que este curso esté bajo una Licencia CC?

Este curso queda bajo una Licencia Creative Commons con las siguientes características:

Ello implica que los derechos de autoría deben ser respetados y no pueden ser obviados en ediciones posteriores de este curso, pero no hay que pedirle permiso a sus autores para reproducirlo. Es decir, si una institución desea reutilizar este curso y hacer una edición de él, puede tomarlo sin solicitar permiso alguno, pero está obligado a mencionar y/o citar a los autores de la versión inicial.

La institución que desee reutilizar este curso puede modificarlo. Sin embargo, debe señalar cuáles modificaciones son a su cargo y qué partes se conservan de los autores originales. Finalmente, se señala enfáticamente que este curso NO puede ser utilizado con propósitos lucrativos ni puede ser comercializado de ninguna manera.

Todo lo anterior posibilita la difusión de manera sencilla de los conocimientos de este curso. Todas aquellas instituciones interesadas en replicarlo son libres de hacerlo bajo las reglas de esta Licencia Creative Commons. Los autores de este curso, cuyos contactos están en los Anexos de este Manual, están en la mejor disposición de asesorar a las instituciones interesadas en hacer ediciones futuras de este proyecto.

ESTE CURSO PODRÁ SER MODIFICADO, REUTILIZADO Y REPLICADO EN TODAS LAS EDICIONES POSTERIORES QUE SE DESEE, SIEMPRE Y CUANDO SE RECONOZCA EL TRABAJO DE SUS AUTORES Y NO SE TENGA UN PROPÓSITO COMERCIAL O DE LUCRO.

6.2 LOS TUTORES COMO PROMOTORES DE CAPACITACIÓN EN LA REGIÓN: CONSTRUYENDO UNA RED Y UNA CULTURA HACIA LA INTEGRACIÓN DEL VIH Y OTRAS ENFERMEDADES INFECCIOSAS PREVALENTES EN EL 1ER. NIVEL DE ATENCIÓN

El seguimiento de la implementación de los proyectos de intervención acción.

Una pregunta obligada es ¿y después del curso qué sigue? Un esfuerzo de tal importancia en nuestra región debe estar acompañado por una responsabilidad conjunta de difusión.

En este aspecto el papel de los tutores y alumnos del curso se convierte en algo esencial.

Tanto los tutores como los participantes, en su rol de co-responsables de la elaboración de los proyectos de intervención acción, deberán velar e impulsar acciones en los países que apoyen a las autoridades de salud y que promuevan la implementación de los proyectos propuestos en los Talleres Nacionales.

Otra forma de impulsar la Integración del VIH y otras enfermedades infecciosas prevalentes en el 1er. Nivel de Atención es promover los proyectos, cuya implementación sea a escala local, dentro de los espacios pertinentes.

Una buena idea para la impulsión de los proyectos puede ser el enfoque de Promoción de la Salud, el cual cuenta con varias estrategias centrales para alcanzar sus objetivos. Entre ellas se destacan:

LOS TUTORES DE ESTE CURSO TIENEN EL COMPROMISO MORAL POSTERIOR DE CONVERTIRSE EN AGENTES Y PROMOTORES DE LA CAPACITACIÓN EN LA REGIÓN.

- Abordaje por Contextos.
- Comunidad y Desarrollo Comunitario.
- Trabajo Interdisciplinario e Intersectorial.
- Educación en Promoción de Salud.
- Comunicación en Promoción de la Salud.
- Políticas Públicas Saludables e Inclusión Social.

Los tutores y participantes de este curso podrán, a través de estrategias como las señaladas anteriormente, promocionar y eventualmente implementar los proyectos o las acciones que se derivan de éstos.

El Portal del Curso.

Otro espacio que proponemos, como lugar de excelencia de encuentro de la comunidad resultante de la 1ra. Edición de este curso, es el Portal del Curso Integración del VIH.

Este Portal tiene varios objetivos:

1. Servir de espacio de comunicación entre el Grupo de Desarrollo Curricular, su Coordinación Académica y los tutores durante la operación de la 1ra. Edición del curso.
2. Servir de espacio de comunicación entre tutores y alumnos en la fase de proyectos.
3. Disponer de un espacio virtual único para la operación del curso (reuniones, actualización de información, intercambio de experiencias, mantenimiento del ritmo de trabajo, entre otros).
4. Aprovechar los recursos de aprendizaje que serán producidos en el marco del curso y la formación de sus tutores, así como

LOS TUTORES Y PARTICIPANTES DE ESTE CURSO PODRÁN, A TRAVÉS DE DIFERENTES ESTRATEGIAS, PROMOCIONAR Y EVENTUALMENTE IMPLEMENTAR LOS PROYECTOS DE INTERVENCIÓN PROPUESTOS O LAS ACCIONES QUE SE DERIVEN DE ÉSTOS.

otros que se propongan, y sistematizarlos para su uso durante la operación del curso y ediciones posteriores.

5. Servir de referente en materia de documentos pedagógicos y objetos de aprendizaje que sean producto de los esfuerzos de este curso y de otros.
6. Sistematizar las experiencias, lecciones aprendidas e información derivada de este curso.
7. Convertirse en un Foro de debate y discusión acerca de la integración del VIH, la educación multimodal, el rol de los tutores, las modalidades educativas, la educación virtual y, en general, de todo lo referente al proceso de enseñanza - aprendizaje en salud.
8. Convertirse en el germen de una comunidad regional de aprendizaje, en tanto red colaborativa que posibilite ediciones posteriores de este esfuerzo, así como se convierta en un instrumento para la capacitación en salud de la Región.

EL PORTAL DEL CURSO BUSCA CONVERTIRSE EN EL GERMEN DE UNA COMUNIDAD REGIONAL EN TANTO RED COLABORATIVA QUE POSIBILITE EDICIONES POSTERIORES DE ESTE ESFUERZO ASÍ COMO SE CONVIERTA EN INSTRUMENTO PARA LA CAPACITACIÓN EN SALUD EN LA REGIÓN.

ANEXOS

ANEXO 1. ACTIVIDADES INICIALES. CUESTIONARIO DE DATOS INICIALES, REFLEXIÓN INICIAL Y LISTA DE COTEJO.

<u>CUESTIONARIO DE DATOS INICIALES</u>

Nombre:

Correo electrónico:

Por favor, diga el nombre del centro donde fue seleccionado para este curso: ____

¿Cuál es su profesión? (marque todas las que correspondan)

- o Médico/a general
- o Médico/a obstetra
- o Médico/a pediatra
- o Coordinador/a del centro
- o Enfermero/a
- o Trabajador/a social
- o Partero/a
- o Otro (por favor, especifique)

Número de años practicando su profesión :

- ○ <1 año
- ○ 1-5 años
- ○ 6-10 años
- ○ 11-15 años
- ○ >15 años

A N E X O S

¿Se especializa en una práctica médica determinada? (marque todas las que correspondan)

- Medicina General
- Medicina Familiar (niños y adultos)
- Investigaciones sobre el VIH/SIDA
- Infectología
- Obstetrica y ginecología
- Pediatría
- Otro (por favor, especifique)

Por favor, seleccione su edad.

- 18-25
- 26-35
- 36-45
- 46-55
- 56+

Su sexo:

- Masculino
- Femenino

¿En cuál sector practica?

- Público
- Seguro Social
- Privado

Promedio de número de pacientes atendidos en su clínica por mes. (Incluyendo a pacientes vistos por usted y otros trabajadores de salud) _____

¿Qué porcentaje de sus pacientes están infectados por el VIH? _____

¿Qué porcentaje de sus pacientes son mujeres embarazadas? _____

Información Demográfica de los pacientes

Edad de los pacientes en su práctica (marque todas las que correspondan):

- Sólo infantes (edad < 1 año)
- Infantes y niños
- Sólo niños (edad > 1 año)
- Sólo adultos (edad > 18 años)
- Mujeres en edad reproductiva (14-45 años)
- Yo no atiendo pacientes.

Acceso a la computadora el internet

¿Dónde tiene acceso a una computadora con conexión a internet? (marque todas las que correspondan)

- Casa
- Trabajo
- Biblioteca
- Café internet
- Otro (por favor, especifique)

¿Con qué frecuencia tiene usted acceso a los siguientes servicios informáticos? (Clasificar como “todo el tiempo”, “diario”, “varias veces a la semana”, “una vez a la semana”, “con menos frecuencia de una vez por semana”)

- Una computadora
- Internet
- Correo electrónico

A N E X O S

REFLEXIÓN INICIAL

Escriba un ensayo corto, entre 2 y 3 páginas, formato Word, y tipo de letra Arial 11 puntos a doble espacio, para identificar los principales factores que afectan la organización e integración de servicios de salud para el control del VIH y otras enfermedades infecciosas prevalentes en su entorno directo de trabajo en el nivel local.

Se valorará la inclusión de contenidos en las siguientes áreas:

- ¿Cuáles son los principales factores del entorno social, político y económico que determinan la situación del VIH y otras enfermedades infecciosas prevalentes?

- ¿Cómo se organiza la atención de salud para el VIH en mi nivel local?

- ¿Cómo se integra en el nivel local la atención del VIH con las estrategias de prevención y promoción en el área materno infantil y de otras enfermedades infecciosas relacionadas?

- ¿Con qué estrategias y herramientas clínicas para el diagnóstico, tratamiento y seguimiento de enfermos de VIH se cuenta en el nivel local y cómo se aplican?

- ¿Qué propuestas básicas de intervención y mejoría sostenible de estos componentes podría plantear al inicio del curso?

Se adjunta lista de cotejo con el detalle de los elementos de contenidos ya señalados y de presentación formal para que sea utilizada como guía durante la redacción del ensayo.

Pan American Health Organization

Se le recuerda al participante que la ÚNICA FORMA DE ENTREGAR esta actividad evaluativa será a través de la plataforma del curso y que su cumplimiento es OBLIGATORIO.

¡Buena suerte!

A N E X O S

LISTA DE COTEJO DE LA REFLEXIÓN INICIAL

Factor	SI	NO	Observaciones
**Área de Contenidos (85 puntos)**			
Se identifican factores del entorno que puedan afectar la situación de salud. (20 puntos).			
Se identifica la organización existente para la entrega de servicios de salud. (20 puntos).			
Se identifica la existencia o no existencia de elementos de integración de servicios. (15 puntos)			
Se describe las acciones clínicas aplicadas en el nivel local para el control y tratamiento del VIH y otras enfermedades infecciosas prevalentes. (15 puntos).			
Se proponen ideas de mejoría. (15 puntos).			
**Área de Presentación (15 puntos)**			
Redacción Clara (5 puntos)			
Uso correcto de la ortografía y la gramática (7 puntos)			
Extensión adecuada (3 puntos)			
**TOTAL**			

ANEXO 2. EVALUACIONES Y LISTAS DE COTEJO DEL TEMA 1.

EVALUACIÓN SEMANA 1

CASO TIERRADENTRO

Elabore un ensayo entre 2 y 3 páginas, formato Word, tipo de letra Arial 11 puntos a doble espacio, para responder la siguiente situación hipotética.

Se adjunta lista de cotejo con el detalle de los elementos de contenidos ya señalados y de presentación formal para que sea utilizada como guía durante la redacción del ensayo.

Se le recuerda al participante que la ÚNICA FORMA DE ENTREGAR esta actividad evaluativa será a través de la plataforma del curso y que su cumplimiento es OBLIGATORIO.

¡Buena suerte!

Antecedentes.

Usted está a cargo del centro de salud de "Tierradentro". Tierradentro es una zona geográfica situada en Centroamérica, que tiene aproximadamente 25.000 personas en la zona de cobertura. Su tarea es planificar las actividades de promoción, prevención, diagnóstico y referencia de las personas viviendo con VIH en su zona de cobertura ahora que existe una política de integración de VIH en todos los niveles de atención.

La zona cuenta además con un centro de atención integral donde habitualmente se realizan estudios de diagnóstico de VIH, atención y tratamiento específico para los afectados de la zona. El centro trabaja desde hace varios años con fondos externos y tiene acuerdos con algunas ONG locales, aunque tiene relativamente pocos pacientes, actuando como centro de referencia y apoyo hacia el suyo en lo relacionado a atención de VIH.

Algunos hechos documentados respecto al Centro de Salud Tierradentro.

En el último año se han diagnosticado 6 niños con infección por VIH en la zona, incluso 1 de ellos falleció. En 4 de estos casos, las mujeres no se habían realizado las pruebas antes del parto. En otro de los casos la madre se había realizado un control prenatal, pero nunca volvió a buscar el resultado y tuvo su bebe en casa. Un año después su hijo presentó una neumonía grave, se diagnostico VIH y falleció a los pocos días. En el último caso, la madre provenía de otra zona cercana a Tierradentro que no tiene implementado programas de control de personas viviendo con VIH.

En el centro de salud de Tierradentro no se hacen pruebas de VIH y solo se remiten al centro de atención integral aquellos casos con sospecha clínica o en los que la madre lo solicita. Aunque la prueba es gratis, las mujeres tienen que llevar su jeringa estéril.

El año pasado se calcula que solo se hizo el HIV a menos del 30% de las mujeres embarazadas de la zona de cobertura.

Pan American Health Organization

También el año pasado se identificaron 16 mujeres con VIH antes del parto, de estas 13 tuvieron algún control. De las 3 que no tuvieron control 2 tuvieron hijos sanos. La mayoría de las que se controlaron vivían en la zona urbana.

En 4 de los 6 casos de hijos HIV positivos las madres vivían en la zona norte, que concentra una gran población indígena, la mayoría de los cuales incluso no habla castellano.

En 5 de los 6 casos las madres tenían menos de 20 años.

En 3 de los 6 no tenían pareja fija.

Todas las madres habían cursado al menos tercer grado de primaria, pero solo 1 tenía algún estudio secundario.

En la población del norte gran parte de las mujeres no realiza control prenatal. Entre las causas referidas figuran la distancia del centro, la negación del marido, la necesidad de quedarse a cuidar al resto de la familia, la falta de conocimiento de la necesidad de hacer los controles, el miedo a tener que tener trabajo de parto acostada ya que es costumbre ancestral que sea en posición vertical, el miedo al maltrato por los médicos, la falta de dinero para pagar los remedios o estudios si se le indican. Una proporción considerable de estas personas no tienen documentos, y un gran porcentaje nacieron en el otro lado de la frontera, aunque viven aquí desde hace muchos años.

Antes de decidir cuáles son las intervenciones más importantes le solicitan a Ud. que identifique a los grupos más vulnerables para VIH en la comunidad y que caracterice los determinantes sociales más importantes que pueden dificultar el acceso temprano al diagnostico de VIH, en particular en mujeres embarazadas.

Elementos a desarrollar en su informe.

1. ¿Es el VIH es un problema importante en esta población? ¿Por qué?
2. ¿Cuáles son los determinantes sociales que están afectando la transmisión materno infantil del VIH en esta localidad?
3. ¿Cuál ha sido la responsabilidad del sistema de salud en estos casos?
4. ¿En esta localidad cuáles son las poblaciones más vulnerables para la transmisión materno infantil?
5. ¿Las mujeres embarazadas indocumentadas o extranjeras tienen derecho a realizar consultas prenatales?

LISTA DE COTEJO SEMANA 1

Factor	SI	NO	Observaciones
**Área de Contenidos (85 puntos)**			
Se describe la situación epidemiológica básica de la población. (10 puntos).			
En el análisis epidemiológico se identifican los grupos de riesgo más vulnerables. (15 puntos).			
Se identifican los determinantes sociales principales que influyen en la situación descrita. (15 puntos).			
Se caracteriza la importancia del VIH para la población afectada. (10 puntos).			
Se identifican propuestas de intervención y mejoría en las áreas anteriores. (10 puntos).			
Se identifican las principales estrategias y herramientas clínicas básicas para el control del VIH y su aplicación en el nivel local. (10 puntos).			

Se especifican los elementos de integración de estrategias de prevención y promoción de VIH con el área materno infantil y de otras enfermedades infecciosas relacionadas. (15 puntos).			
**Área de Presentación (15 puntos)**			
Redacción Clara (5 puntos)			
Uso correcto de la ortografía y la gramática (7 puntos)			
Extensión adecuada (3 puntos)			
**TOTAL**			

EVALUACIÓN SEMANA 2

Lea y analice cuidadosamente el documento de Perfil de Sistema de Salud Correspondiente a su país.

Luego de analizar el documento escriba una reflexión crítica acerca de cómo la situación de salud ahí descrita impacta en el control e integración del VIH y enfermedades infecciosas prevalentes en las acciones y estrategias de salud del país.

El documento debe tener una extensión entre 2 y 3 páginas, tipo de letra Arial de 11 puntos a dos espacios en formato Word, con una redacción clara y revisión ortográfica.

Se adjunta lista de cotejo con el detalle de los elementos de contenidos ya señalados y de presentación formal para que sea utilizada como guía durante la redacción del ensayo.

Se le recuerda al participante que la ÚNICA FORMA DE ENTREGAR esta actividad evaluativa será a través de la plataforma del curso y que su cumplimiento es OBLIGATORIO.

¡Buena suerte!

Pan American Health Organization

LISTA DE COTEJO SEMANA 2

Factor	SI	NO	Observaciones
**Área de Contenidos (85 puntos)**			
Se analiza la estructura y diseño del sistema de salud desde el punto de vista de enfoque de Atención Primaria. (20 puntos).			
Se identifica la existencia o no existencia de elementos de integración para la entrega de servicios de salud (20 puntos).			
Se analiza la presencia o ausencia de diseño de políticas de salud en el marco de una estrategia de protección social. (20 puntos).			
Se proponen ideas de mejoría para el diseño global basadas en el enfoque de APS, integración y estrategia de protección social. 25 puntos).			
**Área de Presentación (15 puntos)**			
Redacción Clara (5 puntos)			
Uso correcto de la ortografía y la gramática (7 puntos)			
Extensión adecuada (3 puntos)			
**TOTAL**			

EVALUACIÓN SEMANA 3

CASO ESTRATEGIAS DE GESTIÓN E INTEGRACION DE PROGRAMAS DE ATENCION DE SALUD. UNA NUEVA ORIENTACION DE LA ATENCION DE SALUD PARA EL CENTRO DE ATENCIÓN PRIMARIA DE SALUD DE LOS LAPACHOS

Elabore un ensayo entre 2 y 3 páginas, formato Word, y tipo de letra Arial 11 puntos a doble espacio, para responder la siguiente situación hipotética.

Se adjunta lista de cotejo con el detalle de los elementos de contenidos ya señalados y de presentación formal para que sea utilizada como guía durante la redacción del ensayo.

Se le recuerda al participante que la ÚNICA FORMA DE ENTREGAR esta actividad evaluativa será a través de la plataforma del curso y que su cumplimiento es OBLIGATORIO.

¡Buena suerte!

Descripción del contexto y de la situación problema

El barrio Los Mangos se encuentra ubicado en la periferia de una gran ciudad. Cuenta con el Centro de Atención Primaria de Salud de Los Lapachos que debiera dar cobertura a 25.000 personas residentes y no residentes del área.

Los indicadores de salud, socioeconómicos y ambientales están marcando la necesidad de intervenciones sanitarias contundentes, a fin de facilitar el acceso de la población a actividades tanto preventivas como curativas de la salud.

El Centro tiene un nuevo jefe, el Dr. Diéguez, que ha interiorizado la situación epidemiológica y ha evaluado la cobertura, recabando la opinión de la comunidad y de otros actores sociales.

Las personas que concurren al Centro se quejan de que deben realizar colas para la atención desde muy temprano, que concurren con niños y que no consiguen citas de atención. Las citas sólo se dan por la mañana y una sola vez en el día, y los profesionales cubren horarios muy restringidos, ya que no existe un número suficiente de profesionales contratados en el centro. En parte por esta dificultad, la comunidad sólo concurre al Centro ante la presencia de enfermedades agudas.

A su vez el personal administrativo se queja de las continuas presiones a las que se ve sometido cuando se acaba la disponibilidad de horas para citas de atención y por su angustia ante la falta de respuesta desde los ámbitos directivos del centro. Recientemente se han suscitado algunas situaciones de violencia que incluso motivaron que un funcionario administrativo pidiera su transferencia a otra dependencia del centro sin contacto directo con la comunidad.

Luego de evaluar estas situaciones y de constatar que la demanda efectiva de atención por parte de la comunidad solo alcanza a un 10% de la población de su área a cargo, y que entre todos los grupos etáreos el de los menores de 6 años, y los adultos entre 18 y 45 años son los que menos consultan, el Dr. Diéguez decide introducir algunos cambios, con la idea de que se estructure un equipo permanente de admisión de personas que pueda facilitar la atención a la comunidad, así como priorizar y aplicar criterios de equidad y calidad de atención en la asignación de citas de atención, más allá del orden de llegada. Al mismo tiempo, se ha considerado que los

profesionales que integrarán este nuevo equipo deben contar con la formación adecuada para responder los reclamos de los vecinos y dar las explicaciones adecuadas.

El equipo, que ha recibido el nombre de Servicio de Orientación de la Demanda de Salud (SODES), estará conformado por trabajadores del centro de diferentes disciplinas que se ocuparán diariamente, de 6.00 a 17.00 horas, de recibir, escuchar y calificar las solicitudes de demandas de atención y citas de las personas y de orientarlas de inmediato a la consulta, o de remitirlas a los diferentes programas para una solución puntual o con una cita diferida próxima.

También deberán hacer el seguimiento semanal de las derivaciones y consultas e informar de las estadísticas de atención. A su vez, se ha decidido extender el horario de atención más allá de las 17:00 y hasta las 20:00 tres veces a la semana para facilitar el acceso de las personas trabajadoras, conformando un sistema de turnos rotativos con un equipo de médico, enfermera y personal técnico de apoyo.

Finalmente, se está desarrollando un modelo de trabajo liderado por la enfermera más experimentada del centro, para organizar la realización de visitas domiciliarias, que se espera una vez definido, permita que personal técnico paramédico viste a las familias del centro para evaluar su grado de necesidad de atención y vulnerabilidad.

Visiones del problema por parte de los actores involucrados.

Los vecinos concuerdan con el Dr. Diéguez en la necesidad de un cambio en el modelo de atención, pero manifiestan su temor ante la aparición de nuevas normas y modalidades de gestión de la atención que terminen en nuevas exigencias y requisitos para ellos como pacientes.

Estas medidas han provocado una reacción negativa por parte de los profesionales y en particular por los médicos, los cuales se resisten a una readecuación de los horarios y han decidido no prestar colaboración no aceptando la modificación y extensión de sus agendas semanales ni la inclusión de pacientes una vez que han iniciado la consulta diaria.

Un grupo mayoritario de profesionales del centro piensan que se trata de una medida autoritaria porque no se les consultó ni sobre el problema ni sobre la solución. Sostienen que hay muchas personas que concurren temprano al centro todos los días porque no entienden la información que se les entrega y no cumplen con las normas ni con las indicaciones recibidas. Por otra parte, postulan que "las demandas médicas requieren médicos y las de salud mental, psicólogos y que un enfermero no puede dar cuenta de estas situaciones, ni le corresponde, de acuerdo al manual de procedimientos".

A su vez un grupo más pequeño y minoritario de profesionales entienden que algo hay que hacer con la gente que no demanda atención y no llega siquiera a traspasar la puerta del Centro, aunque también reclaman por la carencia de entrenamiento práctico para el desarrollo de sus tareas y manifiestan dificultades para cubrir toda la extensión horaria que requiere la nueva modalidad de atención. Los enfermeros se han ofrecido a cubrir los primeros horarios de la mañana para evitar las largas colas. "Nos tendríamos que reunir a planificar esta nueva manera de hacer las cosas".

En términos de recursos, la iniciativa ha recibido discreto apoyo del nivel distrital, que ha manifestado la posibilidad de asignar algunas horas de nuevos profesionales, en la medida que la iniciativa muestre resultados, así como la posibilidad de hacer llegar algunos recursos técnicos en el área de la informática que permitan organizar la entrega de citas de atención a través de un sistema de agenda medica informatizada.

El Dr. Diéguez, piensa que los profesionales deben asumir responsabilidades hacia la comunidad y las personas a su cargo, así como desarrollar sus competencias en forma polivalente, conservando la especificidad de su práctica pero ampliando la esfera de actividades compartidas e interrelacionadas con el resto de los trabajadores del centro. Ante la resistencia que le ofrecen los profesionales, duda sobre si conseguirá aliados o sólo enemigos. Está convencido de que deberá lidiar con el núcleo duro del imaginario profesional, basado en la escasez, "escasos recursos", "escaso tiempo", "escasa capacitación".

LISTA DE COTEJO SEMANA 3

Factor	SI	NO	Observaciones
**Área de Contenidos (85 puntos)**			
Se describe adecuadamente la situación y se identifica nudos críticos. (15 puntos).			
Se aplica el enfoque de ciclo de vida para el diseño de actividades. (15 puntos).			
Se aplican herramientas que fortalezcan los recursos humanos en la ejecución de estrategias de Atención Primaria. (20 puntos).			
Se establecen modelos de participación comunitaria activa en el desarrollo y ejecución de estrategias de Atención Primaria. (15 puntos).			
Se propone una visión de gestión del cambio en las estrategias propuestas. (10 puntos).			
Se incorpora una visión de integración de recursos y servicios para la ejecución de la estrategia propuesta. (10 puntos).			
**Área de Presentación (15 puntos)**			
Redacción Clara (5 puntos)			
Uso correcto de la ortografía y la gramática (7 puntos)			
Extensión adecuada (3 puntos)			
**TOTAL**			

Pan American Health Organization

EVALUACIÓN SEMANA 4

CASO: OPORTUNIDADES PERDIDAS Y ESTRATEGIAS DE INTERVENCION PARA LA PREVENCION DEL VIH EN ADOLESCENTES

Elabore un ensayo entre 2 y 3 páginas, formato Word, y tipo de letra Arial 11 puntos a doble espacio, para responder la siguiente situación hipotética.

Se adjunta lista de cotejo con el detalle de los elementos de contenidos ya señalados y de presentación formal para que sea utilizada como guía durante la redacción del ensayo.

Se le recuerda al participante que la ÚNICA FORMA DE ENTREGAR esta actividad evaluativa será a través de la plataforma del curso y que su cumplimiento es OBLIGATORIO.

¡Buena suerte!

Descripción del contexto y de la situación problema

El Municipio de Loma Alta se encuentra ubicado al sudoeste de una gran ciudad de Latinoamérica y consta de una población de más de 600.000 habitantes, distribuidos en diferentes barrios. Algunos de estos barrios, sujetos de reciente expansión urbana han acogido en los últimos diez años a numerosas familias de trabajadores y empleados, una nueva clase media emergente que busca nuevas oportunidades en base al crédito y al acceso a viviendas subsidiadas por el gobierno.

Otro sector, de habitantes de más antigüedad en el Municipio, consta de población de mayor edad, con una alta proporción de adultos mayores, jubilados de sus empleos que habitan viejas urbanizaciones de más de treinta años de antigüedad.

Finalmente el sector de Canal Bajo es el más pobre, caracterizándose por su marginalidad y aislamiento al estar situado a la orilla de un canal de regadío que cruza el sector sur del Municipio, terrenos estatales con prohibición de ser ocupados, pero que ha ido siendo habitado por numerosas familias de personas sin casa provenientes de toda la ciudad.

Este último sector ha resultado un quebradero de cabeza para la administración de salud del Centro de Atención Primaria (CAP) de Padre Mariano, de reciente creación y a cargo de gran parte de la población del sector del Canal Bajo. El nivel de recursos humanos y financieros con que cuenta el CAP Padre Mariano es similar a los otros centros de salud del Municipio, pudiendo observarse una mayor rotación de personal de salud fundamentalmente médicos. El resto de los recursos humanos suele ser más estable, aunque se quejan del bajo nivel de incentivos y la variabilidad de los medicamentos e insumos que recibe el Centro.

La población a su cargo suele consultar solo ante situaciones de enfermedad aguda, no tiene mayor interés en las actividades preventivas desarrolladas en el (CAP) y rara vez consulta para recibir las consultas o vacunas que el programa nacional de salud infantil tiene a su disposición.

Uno de los mayores problemas identificados por la Enfermera a Cargo de los programas de control Prenatal es el gran número de jóvenes y adolescentes de la población de Canal Bajo, entre los que las dinámicas de consulta ya descritas son aún peores. Esto se ve agravado por el gran número de embarazos de jóvenes y adolescentes que no reciben ningún control y de los cuales el personal de salud solo se entera cuando concurren para atención de urgencia ante una amenaza de parto prematuro o un trabajo de parto ya desencadenado. El personal de salud sabe que además la prevalencia de enfermedades de transmisión sexual, incluyendo la Sífilis y el VIH son de alta prevalencia entre la población de Canal bajo, producto de la información que se produce en el Hospital Distrital.

Un problema que da aún mayor complejidad es la alta tasa de delincuencia entre los habitantes de Canal Bajo. Una parte de su población vive del microtráfico de drogas, actuando como abastecedores de la población con más ingresos del Municipio de Loma Alta, e incluso de los habitantes de la ciudad que concurren a puntos de venta de drogas conocidos. No son infrecuentes las redadas policiales y la incautación de droga, a pesar de lo cual el tráfico parece continuar sin mayores sobresaltos.

Los trabajadores de salud del CAP de Padre Mariano no tienen consenso acerca de las acciones a desarrollar hacia la población de Canal Bajo. En particular los médicos atienden con reticencia a los pacientes provenientes del sector, estigmatizados por su bajo nivel cultural y su pobreza, y por ser en muchas ocasiones agresivos y amenazantes con el personal de salud. Varias veces médicos del CAP se han negado a atender pacientes potencialmente peligrosos y no ha sido rara la necesidad de llamar a la policía para detener a pacientes agresivos al interior del centro de salud.

Las Enfermeras y Matronas a cargo de los programas infantil y de la mujer tienen una actitud y experiencia más positiva, pero también llena de contrastes. Hace dos años atrás, una Enfermera de Sector logro por algunos meses que las consultas al programa de vacunación y control infantil se incrementaran sustancialmente, aparentemente por su buen contacto y trato con algunas madres que actuaban como líderes de los “comités de gente sin casas” que logro identificar y convencer de los aspectos positivos de estas acciones. Durante algunos meses logró desarrollar talleres de educación en salud y formación de líderes comunitarios en la casa de una de las madres, y en colaboración con una profesora de la Escuela vecina a Canal Bajo que recibe a algunos de los niños que asisten a clases. A los talleres concurrieron varias mujeres mayores de Canal Bajo y un par de jóvenes madres. Sin embargo este periodo terminó cuando la Enfermera decidió renunciar desanimada por el bajo nivel de apoyo y respuesta de los médicos que debían resolver las interconsultas locales de su población de niños a cargo. Otro factor fue que debía trasladarse por sus propios medios varias calles hacia el interior de Canal Bajo lo que la hizo temer por su seguridad personal en varias ocasiones.

Esta semana un incidente causó cierto revuelo entre las Matronas, Enfermeras y Director del centro. Se trataba del caso de una joven de quince años llamada Julieta que había llegado al CAP hace dos meses en trabajo de parto, sin ningún control de su embarazo, siendo derivada de urgencia en un vehículo de transporte al hospital más cercano. Ahora volvía con su niño de dos meses en un lamentable estado de desnutrición y con síntomas de una enfermedad respiratoria baja grave. La enfermera que la recibió fue la misma que atendió su trabajo de parto inicial, pudiendo constatar que el niño no había realizado sus controles, a pesar de que la madre recibió indicaciones de control en su Centro de Atención

primaria al haber sido dada de alta en el hospital. Una situación mucho más compleja aún fue que la madre refirió que el padre del niño se encontraba detenido ya hacía ocho meses y que aparentemente el último mes se le había diagnosticado VIH en la cárcel.

Basándose en el caso ya descrito, comente:

- Principales factores condicionantes y variables que usted identifica en la situación descrita y especialmente en el caso de Julieta.
- Oportunidades perdidas de intervención que identifica en el caso de Julieta.
- ¿Qué medidas clínicas habría que recomendar a la madre y al niño para determinar el riesgo de VIH y sífilis y otras infecciones?
- ¿Qué tipo de seguimiento necesitaría esta madre y su niño?
- ¿Qué cambios en la organización de servicios propondría para que este tipo de situaciones no se repita en este CAP?
- ¿Qué actividades de monitoreo implementaría para estar seguro de que estas situaciones no se están repitiendo?
- ¿Cómo podría trabajar con el hospital distrital para evitar estas situaciones?

LISTA DE COTEJO SEMANA 4

Factor	SI	NO	Observaciones
**Área de Contenidos (85 puntos)**			
Se identifican estrategias de promoción y prevención de salud sexual y reproductiva hacia la población general. (25 puntos).			
Se identifican estrategias y acciones específicas de hacia los adolescentes y grupos de mayor riesgo. (30 puntos).			
Se especifican estrategias y acciones de integración de servicios. (30 puntos).			
**Área de Presentación (15 puntos)**			
Redacción Clara (5 puntos)			
Uso correcto de la ortografía y la gramática (7 puntos)			
Extensión adecuada (3 puntos)			
**TOTAL**			

ANEXO 3. ACTIVIDADES FINALES. REFLEXIÓN FINAL Y LISTA DE COTEJO. CUESTIONARIO DE EVALUACIÓN DE LA FASE VIRTUAL DEL CURSO.

REFLEXIÓN FINAL

Escriba un ensayo corto, entre 2 y 3 páginas, formato Word, y tipo de letra Arial 11 puntos a doble espacio, para que nos comente, después de haber terminado este curso virtual, ¿cuáles son los principales factores que afectan la organización e integración de servicios de salud para el control del VIH y otras enfermedades infecciosas prevalentes en su entorno directo de trabajo en el nivel local y qué podría hacerse para mejorar la integración?

Se valorará la inclusión de contenidos en las siguientes áreas:

- ¿Cuáles son los principales factores del entorno social, político y económico que determinan la situación del VIH y otras enfermedades infecciosas prevalentes?

- ¿Cómo se organiza la atención de salud para el VIH en mi nivel local?

- ¿Cómo se integra en el nivel local la atención del VIH con las estrategias de prevención y promoción en el área materno infantil y de otras enfermedades infecciosas relacionadas?

- ¿Con qué estrategias y herramientas clínicas para el diagnóstico, tratamiento y seguimiento de enfermos de VIH se cuenta en el nivel local y cómo se aplican?

- ¿Qué propuestas básicas de intervención y mejoría sostenible de estos componentes podría plantear al inicio del curso?

Se adjunta lista de cotejo con el detalle de los elementos de contenidos ya señalados y de presentación formal para que sea utilizada como guía durante la redacción del ensayo.

Se le recuerda al participante que la ÚNICA FORMA DE ENTREGAR esta actividad evaluativa será a través de la plataforma del curso y que su cumplimiento es OBLIGATORIO.

¡Buena suerte!

LISTA DE COTEJO

Factor	SI	NO	Observaciones
**Área de Contenidos (85 puntos)**			
Se identifican claramente los principales factores del entorno social, político y económico, en un enfoque de determinantes sociales. (20 puntos)			
Se identifican los elementos básicos de la organización para la atención de salud para el VIH en el nivel local con un enfoque de desarrollo de la Atención Primaria. (20 puntos)			
Se especifican los elementos de integración de estrategias de prevención y promoción de VIH con el área materno infantil y de otras enfermedades infecciosas relacionadas. (15 puntos)			
Se identifican las principales estrategias y herramientas clínicas para el control del VIH y su aplicación en el nivel local. (15 puntos)			
Se identifican claramente propuestas de intervención y mejoría en las áreas anteriores. (15 puntos)			
**Área de Presentación (15 puntos)**			
Redacción Clara (5 puntos)			
Uso correcto de la ortografía y la gramática (7 puntos)			
Extensión adecuada (3 puntos)			
**TOTAL**			

A N E X O S

CUESTIONARIO DE EVALUACIÓN DE LA FASE VIRTUAL DEL CURSO

Su experiencia con este curso virtual

En cuanto a la estructura del curso, . . . (conteste “Totalmente de acuerdo”, “De acuerdo”, “No tengo opinión”, “En desacuerdo”, “Totalmente en desacuerdo”)

- Los objetivos del curso definieron claramente el contenido del curso y las expectativas.
- Los temas de las conferencias progresaron sensiblemente.
- El curso se organizó de una manera que facilitó aprendizaje
- Otros comentarios sobre la estructura del curso (rellenar)

Por favor, evalúe el contenido del curso (conteste “Totalmente de acuerdo”, “De acuerdo”, “No tengo opinión”, “En desacuerdo”, “Totalmente en desacuerdo”, “No se aplica”)

- El contenido del curso fue útil para mi clínica..
- El material del curso mejoró mi capacidad para tratar a los pacientes VIH positivos.
- La duración de las conferencias fue adecuada.
- El número de conferencias fue apropiado.
- Los foros contribuyeron a mi aprendizaje.
- Fue difícil mantenerse al día con el trabajo del curso debido a responsabilidades familiares y laborales.
- Otros comentarios sobre el contenido del curso (rellenar).

Por favor, evalúe el valor del curso (conteste “de gran valor”, “valioso”, “no valioso”, “no lo usé”)

- Las conferencias grabadas.
- Las diapositivas y los archivos pdf para descargar.
- Las transcripciones y los archivos pdf para descargar.
- El material de lectura para descargar.
- El foro de discusión en línea.
- Las actividades de reflexión de los módulos de Tema 1.
- Las pruebas pre y post (actividades evaluativas) de los módulos de Tema 2.
- Ayuda y aportación de los tutores.

Por favor, evalúe su experiencia acerca del sitio web del curso http://cursospaises.campusvirtualsp.org (conteste “Totalmente de acuerdo”, “De acuerdo”, “No tengo opinión”, “En desacuerdo”, “Totalmente en desacuerdo”, “No se aplica”)

- El proceso de registro fue fácil.
- El sitio web está bien organizado.
- El acceso a las videoconferencias fue fácil.
- Las pruebas pre y post contribuyeron a mi aprendizaje.
- La estructura de clasificación fue justa y razonable.
- El foro fue una buena herramienta de comunicación.
- El chat fue una buena herramienta de comunicación.
- Comunicación por correo con los tutores fue fácil y eficiente.
- Recibir un certificado al final del curso es importante para mí.
- Otro (por favor, especifíque)

Por favor, evalúe los foros semanales (conteste “Totalmente de acuerdo”, “De acuerdo”, “No tengo opinión”, “En desacuerdo”, “Totalmente en desacuerdo”, “No se aplica”)

- Fueron fáciles para mí participar en ellos.
- Fueron dinámicos y accesibles a hacer preguntas.
- Recibimos respuestas en un tiempo apropiado.
- Las respuestas eran útiles.
- Se presentaron casos prácticos e instructivos.
- El foro fomentó discusiones interesantes entre los alumnos.
- Otro (por favor, especifíque)

Por favor, evalúe a los tutores (conteste “Totalmente de acuerdo”, “De acuerdo”, “No tengo opinión”, “En desacuerdo”, “Totalmente en desacuerdo”, “No se aplica”)

- Los tutores facilitaron el proceso de aprendizaje.
- El número de alumnos por tutor era adecuado.
- Los tutores respondieron las preguntas y correos de los alumnos a tiempo y con información correcta.
- Los tutores clarificaron los objetivos del curso y responsabilidades de los alumnos.
- Los tutores corrigieron los trabajos imparcialmente y proporcionaron sugerencias instructivas.

Por favor, marque su nivel de acuerdo con las siguientes frases (“Totalmente de acuerdo”, “De acuerdo”, “No tengo opinión”, “En desacuerdo”, “Totalmente en desacuerdo”)

- Confío en mi capacidad para cuidar de los pacientes con VIH.
- Confío en mi capacidad para proporcionar asesoramiento a pacientes sobre la prueba de VIH.

- Confío en mi capacidad para proporcionar resultados de la prueba del VIH (positivo o negativo) a pacientes.
- Confío en mi entendimiento de la transmisión del VIH.
- Confío en mi capacidad para realizar una evaluación integral inicial de un paciente con VIH.
- Confío en mi capacidad para comenzar un régimen antirretroviral inicial en un paciente con VIH.
- Confío en mi comprensión de la toxicidad antirretroviral.
- Confío en mi capacidad para alentar a los pacientes que se adhieran a sus antirretrovirales.
- Confío en mi capacidad de saber qué intervenciones positivas de prevención son necesarias para mis pacientes con el VIH.
- Confío en mi entendimiento de cómo controlar mejor mis pacientes tomando antirretrovirales.
- Confío en mi capacidad para detectar la TB en todos los pacientes con VIH.

Por favor, evalúe cómo va a utilizar este curso en su trabajo (“Totalmente de acuerdo”, “De acuerdo”, “No tengo opinión”, “En desacuerdo”, “Totalmente en desacuerdo”,”No se aplica”)

- Voy a cuidar a mis pacientes infectados por el VIH de manera diferente como resultado de este curso.
- Tengo una comprensión peor de asesoramiento y pruebas del VIH después de este curso.
- Voy a alentar la adherencia a TARV más con mis pacientes como resultado de este curso.
- Voy a vigilar mis pacientes en tratamiento antirretroviral de manera diferente como resultado de este curso.
- Voy a fomentar la prevención más positiva con mis pacientes como resultado de este curso.
- Voy a probar más a las mujeres embarazadas por el VIH y la sífilis.

- Voy a probar todos los pacientes con la TB para el VIH.
- Voy a examinar a todos los pacientes con VIH para los síntomas de la tuberculosis activa en cada visita.

Planificación de cursos en el futuro

Por favor, ayuda a mejorar y planificar los próximos cursos (“Totalmente de acuerdo”, “De acuerdo”, “No tengo opinión”, “En desacuerdo”, “Totalmente en desacuerdo”,”No se aplica”)

- Yo recomendaría este curso a un amigo o colega.
- Tomaría un curso similar de nuevo.
- La sesión/foro de Q&A fue importante para mí
- Los tutores fueron importantes para mí.

Me gustaría tomar otro curso en línea sobre (clasificar como “Alta prioridad”, “Algo interesado”, “No tengo opinión”, “No muy interesado”, “baja prioridad”, “No se aplica”)

- Prevención de la Transmisión Materno-infantil del VIH
- Cuidado de la Mujer Embarazada con VIH
- Estudios de Laboratorio y Otros Diagnósticos en el Cuidado del Paciente con VIH
- Co-infección con VIH y la Tuberculosis
- Otro (por favor, especifíque)

Estamos teniendo dificultades para apoyar estos cursos. Por favor, háganos saber de cualquier fuente de financiamiento que puedan estar interesadas en apoyar más cursos.

ANEXO 4: INSTRUMENTO DE EVALUACIÓN DE LA FASE PRESENCIAL: DIARIO DE APRENDIZAJE

Estimado alumn@ del Curso” Integración del VIH y otras enfermedades prevalentes en el 1er. nivel de atención”:

El siguiente documento en su totalidad constituirá su evidencia de aprendizaje (evaluación) de la fase presencial de este curso. Se calificará sobre 100 puntos y acumulará 15 puntos en puntuación total del curso.

Se llama Diario de Aprendizaje y consistirá en que cada día usted anote los aprendizajes más significativos que considera que tuvo y explique por qué. En caso de considerar que no aprendió nada también deberá justificar detalladamente el por qué.

Como se puede apreciar la actividad a realizar es sencilla, sin embargo, exigirá que usted reflexione, sintetice y concentre sus ideas. Cada día, al terminar las actividades de trabajo, deberá dedicar como mínimo media hora para completar este diario. Esta actividad es obligatoria y su entrega debe ser diaria a su tutor. El no cumplimiento estricto de esto implicará que Usted no pueda continuar en el curso.

Le deseamos éxitos y lo invitamos a continuar con nosotros en esta maravillosa experiencia.

Muchos saludos y bienvenido a Panamá,

Coordinación Académica

CADA DÍA:

(ESTA ACTIVIDAD DEBE SER ENTREGADA A SU TUTOR A MÁS TARDAR EL CADA DÍA A LAS 9 A.M.)

Nombre: ____

País: ____

Tutor: ____

De lo aprendido en el día de hoy, diga las tres cosas que más le impactaron:

____

____

____

Explique por qué lo señalado anteriormente fue lo que más le impactó. En caso de que considere que no aprendió nada nuevo, por favor, justifique ampliamente su respuesta.

____

____

____

Finalmente, por favor, explique en qué medida y por qué lo aprendido en el día de hoy es útil en su trabajo diario y cómo piensa aprovecharlo.

____

____

____

____

ANEXO 5: RÚBRICA PARA LA EVALUACIÓN DE LOS PROYECTOS Y SU RESUMEN

RUBRICA CONTENIDO DEL PROYECTO

	MUY BUENO<br>2 puntos	BUENO<br>1 punto	ACEPTABLE<br>0.5 puntos	DEFICIENTE<br>0.1 puntos
1. Titulo	Claro, explicativo, responde las preguntas del cuándo, cómo, dónde, con quién se realizará la intervención y el proyecto a desarrollar.<br>Entendible, coherente con la orientación del proyecto el trabajo, está bien redactado es conciso y claro.	Responde las tres preguntas pero requiere mejorar.<br>Relacionado con el proyecto y bien redactado	Requiere incorporar alguna de las respuestas clave y/o tiene dificultad con la claridad en su enunciado.	Enunciado con estructura que no reúne las características establecidas.
2. Objetivos	Se desprenden claramente de la situación problemática identificada.<br>Se especifican situaciones susceptibles de ser alcanzadas y logradas con las intervenciones que serán propuestas con el proyecto de forma pertinente.<br>Los objetivos específicos se derivan de los objetivos generales.	Se desprenden claramente de la situación problemática identificada que se pretende modificar.<br>Son medibles y alcanzables. pero carecen de algún elemento requerido.<br>Son pertinentes y suficientes pero no están relacionados.	Se desprenden de la situación problemática pero falta identificar lo que se requiere modificar.<br>Los objetivos específicos no se relacionan al problema.	Los objetivos no se desprenden de la situación problemática o no son pertinentes ni apropiados.

3. Descripción ampliada de la propuesta (exploración en el entorno y exploración bibliográfica).	La intervención se plantea como consecuencia lógica de los objetivos.<br>Muestra que se realizó una exploración exhaustiva.<br>La intervención es clara, específica, concreta y susceptible de medición<br>Tanto la descripción como la fundamentación son suficientes, pertinentes y están relacionadas entre sí.<br>Logra plasmar con claridad en qué consiste su proyecto de intervención-acción, incluyendo elementos del contexto y en coherencia con el título, los objetivos, la fundamentación y metodología.	La intervención carece de algún elemento requerido.<br>Logra plasmar con claridad en qué consiste su proyecto de intervención-acción, incluyendo elementos del contexto o coherencia con los otros elementos de la propuesta (el título, los objetivos, la justificación y metodología)	La intervención carece de varios elementos requeridos.<br>Describe con poca claridad en qué consiste el proyecto de investigación-acción sin elementos del contexto ni coherencia con los otros elementos de la propuesta	La intervención no es clara ni se desprende de los objetivos ni es susceptible de medición.<br>No describe con claridad en qué consiste el proyecto de investigación-acción
4. Resultados esperados	Se expresan completa y claramente los resultados esperados en la intervención y están relacionados con los objetivos propuestos, especificando los grupos beneficiados y el alcance de los resultados (temporalidad y profundidad)	Cumple con la mayor parte de lo expresado en el indicador anterior.	Cumple al menos con la mitad de lo expresado en el indicador máximo.	Cumple con una tercera parte o menos de lo expresado en el indicador máximo.
5. Diseño metodológico para medir los resultados esperados	Se muestra en forma organizada, clara y precisa, cómo se alcanzarán cada uno de los objetivos específicos propuestos con instrumentos adecuados con indicadores específicos y medibles.<br>Deben detallarse los procedimientos, técnicas y demás estrategias metodológicas requeridas para la intervención.	La metodología carece de algunos elementos solicitados o de la medición de resultados.	La metodología carece de varios de los elementos señalados, así como de la medición.	La metodología es vaga o imprecisa y no brinda elementos para evaluar la pertinencia.

6. Cronograma	Señala las etapas y actividades a realizar.<br>Es factible, flexible y garantiza la culminación de las actividades en el tiempo establecido en la intervención.<br>Plantea su fecha de inicio y su fecha de término.<br>Viable, coherente y flexible.	Carece de alguno de los elementos anteriores.	Carece de varios de los elementos anteriores.	No tiene una adecuada señalización de las etapas del proyecto.<br>Presenta de manera incoherente, incompleta. No es viable o coherente.
7. Presupuesto	Es viable, define los rubros de inversión, desglosa los costos e identifica fuentes de financiamiento.<br>Planteamiento de los recursos humanos, económicos, y materiales, de manera realista y factible.<br>Considera la sostenibilidad del proyecto.	El presupuesto carece de alguno de los elementos solicitados.<br>Falta considerar algún elemento en su presupuesto de manera realista o factible.	El presupuesto carece de varios de los elementos solicitados<br>El planteamiento de los recursos humanos, económicos, y materiales no realista o es poco viable.	El presupuesto planteado es irreal.<br>No identifica costos.<br>No identifica fuentes de financiamiento.

RÚBRICA RESUMEN DEL PROYECTO

	MUY BUENO<br>6 puntos	BUENO<br>4 puntos	ACEPTABLE<br>2 puntos	DEFICIENTE<br>1 punto
Resumen del proyecto	Muestra capacidad de abstracción. Resume en no más de 1 página las ideas principales del proyecto. Es sencillo, claro, sintético. Atractivo. Excelente redacción y ortografía.	Carece de alguno de los elementos anteriores.	Carece de varios de los elementos anteriores.	Carece de la mayor parte de los elementos anteriores.

ANEXO 6: CONTACTOS

(por orden de procesos)

Dr. Omar Sued
OPS
suedomar@paho.org
Tema 1 – Operación y Coordinación general. Proyectos de intervención acción.

Dra. Juana E. Suárez Conejero
Consultora OPS
jesconejero@hotmail.com
Tema 1 - Operación y Modelo pedagógico. Montaje del curso en plataforma. Proyectos de intervención acción.

Dr. Roberto Tapia
Consultor OPS
rtapia2005@gmail.com
Tema 1 - Evaluaciones y listas de cotejo. Proyectos de intervención acción.

Dra. Kathleen Page
Universidad Johns Hopkins
kpage2@jhmi.edu
Tema 2 - Operación y Modelo pedagógico.

Dra. Tamara Flys
Universidad Johns Hopkins
tflys1@jhmi.edu
Tema 2 - Operación y Modelo pedagógico.

Dra. Rosalba de Rodríguez
Instituto Gorgas
rosalba.d.rodriguez@gmail.com
Tema 3 - Operación y Modelo pedagógico.. Coordinación general. Proyectos de intervención acción.

Dr. Baudilio Jardines
Campus Virtual OPS
jardinjo@pan.ops-oms.org
Plataforma tecnológica: Campus Virtual de la OPS. Proyección y operación.

Ing. Madgledis Almanza
Campus Virtual OPS
madgledis@hotmail.com
Plataforma tecnológica: Campus Virtual de la OPS. Soporte técnico de las plataformas (todos los temas).

Ing. Jane McKenzie-White
Universidad Johns Hopkins
jmw@jhmi.edu
Plataforma tecnológica. Soporte Tema 2.

Ing. Mónica Ranta
mranta1@jhmi.edu
Universidad Johns Hopkins
Plataforma tecnológica. Soporte Tema 2.

BIBLIOGRAFÍA

1. AAVV (1997). Evaluación de la educación primaria .Informe. Madrid: INCE-MEC.
2. AAVV (1998). Diagnóstico General del Sistema Educativo. Informe. Madrid: INCE-MEC
3. ALKIN, M.C. – R.H. DAlLLACK (1991). Evaluación. Desarrollo de modelos. En Enciclopedia de la Educación. Barcelona: Vicens Vives-MEC.
4. ASCHBACHER D.- R.H.WINTER (1992). A practical guide to alternative assessment. Virginia: Alexandria ASCD.
5. BARRIOS, O. (2000). Estrategia del portafolio del alumnado. En De la Torre y O. Barrios.
6. Estrategias didácticas innovadoras. Barcelona: Octaedro.
7. BECKELEY, W.L. (1997). Creating a classroom portfolio system. A guide to assist classroom teachers in kindergarten through eighth grade. Iowa, Kendall/Hunt Publishing Company.
8. BELL, D. (1976). Vers la societé post-industrielle. París: Lafont.
9. BIGGS, J. (1996). Assessment and evaluation in Higher Education.
10. BORDAS, I. (1998). Evaluación de programas de innovación. En Jiménez, B. (coord) Evaluación de programas de educación, centros y profesores. Madrid: Síntesis.
11. BORDAS, I.- O. BARRIOS (2000). Sistema de evaluación de los aprendizajes En De la Torre, S. y O. Barrios. Estrategias didácticas innovadoras. Barcelona, Octaedro.
12. CABRERA, F. (2000). Evaluación de la formación. Madrid: Síntesis.

13. CABRERA, F. (1996). Precisiones conceptuales sobre la evaluación de la formación en las organizaciones. Barcelona, Documentos de gestión de calidad: Forum de Empresas de Calidad.
14. CAMILLONI, A.R. y otros (1998). La evaluación de los aprendizajes en el debate didáctico contemporáneo. Barcelona: Paidós.
15. CARDINET, J. (1990). Les contraditions de l'evaluation scolaire. Neuchâtel, IRDP.
16. CASANOVA, M.A. (1992). La evaluación, garantía de calidad para el centro educativo. Zaragoza: Edelvives.
17. CONFERENCIA DE MINISTROS DE LA EDUCACIÓN de la OCDE, 1991.
18. CROLL, P. (1995). La observación sistemática en el aula. Madrid: La Muralla.
19. DE FINA, A.A. (1992). Portafolio assessment. Getting started. New York: Scholastics Inc.
20. DE LA ORDEN, A. (1995). Innovación e investigación en el ámbito educativo, Bordón.
21. DE LA ORDEN, A. (1999). Que educación para que sociedad. Un congreso ante el cambio de milenio. Bordón.
22. DELORS, J. (ed) (1996). La educación encierra un tesoro. Madrid: Santillana.
23. ESTEBARANZ, A. (1994). Didáctica e innovación curricular. Sevilla: Publicaciones de la Universidad.
24. EUROPEAN COMMISSION (2000). European Report on Quality of School Education.
25. Sixteen quality Indicators. Luxembourg: Official Publications of the European Communities.

26. FARR, R. – B. TONE (1994). Portafolio. Performance Assessment. Helping students evaluate their progress as readers and writers. For Worth: Harcourt Brace College Publishers.
27. FAVRE, B.- Ph. PERRENAUD (1988) Analyse des practiques et evaluation formative d'un curriculum. Dijon: INRAP.
28. FETTERMANT, D.M. (1996). Empowerment evaluation. An introduction tho theory and Practice. En D. M. Fettermant, S.J. Kaftarian - A. Wandersman (eds). Empowerment Evaluation. London: Sage publ.
29. FISCHER, Ch.F. – R.M. KING (1995). Authentic Assessment. A guide to implementation. Thousand Oaks, CA: Corwin Press, Inc.
30. GALTON – SIMON - CROLL (1980). Inside the Primary Classroom. London: Routledge.
31. HADJI, C. (1991). L'evaluation des actions éducatives. París: PUF.
32. HARGREAVES, A.- L. EARL - J. RYAN (1999). Una educación para el cambio. Barcelona: Octaedro.
33. HERNÁNDEZ PINA, F. (1996). La evaluación de los alumnos en el contexto de la evaluación de la calidad de las universidades Revista de Investigación Educativa, 14, 2.
34. HUTMACHER, W. (1998). L'avaluació en la transformació de les modalitats de govern dels sistemes educatius. Tendències europees. Avaluació i educació. Barcelona, Generalitat de Catalunya.
35. INSPECCIÓ D'ENSENYAMENT (2000). Avaluació externa del centres. Barcelona, Generalitat de Catalunya.
36. JORBA, J. – N. SANMARTÍ (1996). Enseñar, aprender y evaluar: un proceso de relación continuada. Madrid: Publicaciones del MEC.

37. MAJÓ, J. (1997). Chips, cables y poder. Barcelona: Planeta.
38. MARCHESI, A. – E. MARTÍN (1998). Calidad de la enseñanza en tiempos de cambio Madrid: Alianza Edit.
39. MARCELO, C. (1994). Formación del profesorado para el cambio educativo. Barcelona, PPU.
40. MEDINA, A. – J. CARDONA – S. CASTILLO – M.C. DOMINGUEZ (1998). Evaluación de los procesos y resultados del aprendizaje de los estudiantes. Madrid: UNED.
41. MUNTANER, J. (dir) (1998). Resultats acadèmics de l'alumnat de 14 i 16 anys a Catalunya. Barcelona: Generalitat de Catalunya.
42. NOVAK, D.A. – D.B. GOWIN (1988). Aprendiendo a aprender. Barcelona: Martínez Roca.
43. NOVAK, J.D. (1982). Teoría y práctica de la educación. Madrid: Alianza Edit.
44. ONTORIA, A. (1992). Mapas conceptuales: una técnica para aprender. Madrid: Narcea.
45. ONTORIA, A- J.P. GÓMEZ – A. MOLINA (1999). Potenciar la capacidad de aprender a pensar. Madrid: Narcea.
46. PIKE, K. – S.J. SALEND (1995). Authentic Assessment strategies. Alternatives to normreferenced testing. Teaching Exceptional Children. Fall.
47. PRZEMYCKI, H. (1971). Pédagogie Différenciée. Paris: Hachette.
48. RUBIES, M. –I. BORDAS – M. MUNTANER (1991). Avaluar per Innovar. Barcelona: Generalitat de Catalunya.
49. SABIRÓN, F. (dir) (1999). El discurso y la práctica en evaluación. Propuesta para la deconstrucción y reconstrucción de las prácticas evaluadoras. Zaragoza: ICEUniversidad de Zaragoza.

50. SHAKLEE, B.D – N.E. BARBOUR – R. AMBROSE – S. HADFORD (1997). Designing and ising potafolios. Boston: Allyn and Bacon.
51. SANTILLANA (1998). Aprender para el futuro. Madrid: Fundación Santillana.
52. SANTOS GUERRA, M.A. (1990). Hacer visible lo cotidiano. Teoría y práctica de la evaluación cualitativa de centros escolares. Madrid: Akal.
53. SOLER, E. (1995). Control de calidad e innovación educativa. Bordón.
54. TORRE S. de la – O. BARRIOS (2000). Estrategias didácticas innovadoras. Recursos para la formación y el cambio. Barcelona: Octaedro.
55. TORRE, S. de la (1999). Currículum para el cambio. Bordón.
56. TORRE, S. de la (1993). Aprender de los errores. Madrid: Escuela Española.
57. VILLAR ANGULO, L.M. (1994). Manual de entrenamiento: evaluación de procesos y actividades educativas. Barcelona: PPU.
58. VILLARINI, A (1996). 1er. Seminario taller sobre fundamentos y principios de evaluación auténtica. República Dominicana: Facultad Autónoma de Santo Domingo.
59. ZABALZA, M.A. (1998). Los diarios de clase. Barcelona: PPU.
60. ZABALZA, M.A. (1998). Innovación y cambio en los centros educativos. Santiago de Compostela: Micat.
61. ZABALZA, M.A. (1990). Evaluación orientada al perfeccionamiento. Revista Española de Pedagogía. 186.
